# Quantum Chemical Design Guidelines for Absorption and Emission Color Tuning of *fac*-Ir(ppy)_3_ Complexes

**DOI:** 10.3390/molecules23030577

**Published:** 2018-03-05

**Authors:** Yoshiki Natori, Yasutaka Kitagawa, Shogo Aoki, Rena Teramoto, Hayato Tada, Iori Era, Masayoshi Nakano

**Affiliations:** 1 Department of Materials Engineering Science, Graduate School of Engineering Science, Osaka University, Toyonaka, Osaka 560-8531, Japan; yoshiki.natori@cheng.es.osaka-u.ac.jp (Y.N.); shogo.aoki@cheng.es.osaka-u.ac.jp (S.A.); rena.nishikubo@cheng.es.osaka-u.ac.jp (R.T.); hayato.tada@cheng.es.osaka-u.ac.jp (H.T.); iori.era@cheng.es.osaka-u.ac.jp (I.E.); 2 Center for Spintronics Research Network (CSRN), Graduate School of Engineering Science, Osaka University, Toyonaka, Osaka 560-8531, Japan

**Keywords:** phosphorescent light-emitting materials, time-dependent density functional theory (TD-DFT), optical property, *fac*-Ir(ppy)_3_ complex, substituent effect

## Abstract

The *fac*-Ir(ppy)_3_ complex, where ppy denotes 2-phenylpyridine, is one of the well-known luminescent metal complexes having a high quantum yield. However, there have been no specific molecular design guidelines for color tuning. For example, it is still unclear how its optical properties are changed when changing substitution groups of ligands. Therefore, in this study, differences in the electronic structures and optical properties among several substituted *fac*-Ir(ppy)_3_ derivatives are examined in detail by density functional theory (DFT) and time-dependent DFT (TD-DFT) calculations. On the basis of those results, we present rational design guidelines for absorption and emission color tuning by modifying the species of substituents and their substitution positions.

## 1. Introduction

**Figure 1:**
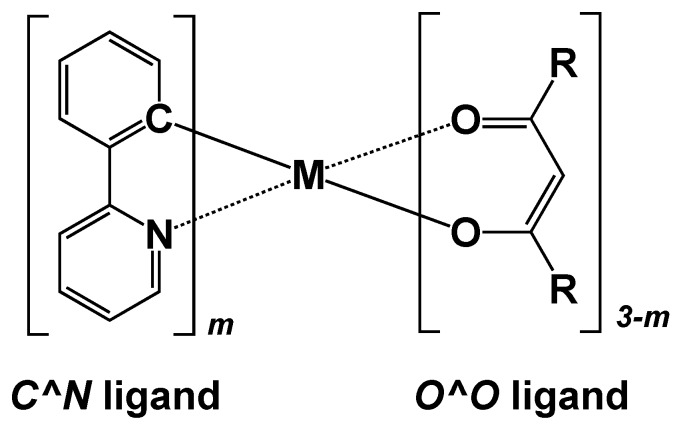
Structural framework of typical phosphorescent complexes, substituted *fac*-M(ppy)*_m_*(0<*m*≤3).

Recently, electroluminescent (EL) materials are often used for devices such as flat panel displays and illuminations. Ir(III) complexes have attracted much attention as important chromophores for such organic EL devices due to their high phosphorescence quantum yields [1,2,3,4]. *fac*-Ir(ppy)_3_ complex—where ppy denotes 2-phenylpyridine—is one of the well-known luminescent metal complexes having a high quantum yield [5,6,7,8,9]. However, there have been no specific molecular design guidelines for absorption and emission color tuning; for example, dependences of the optical properties on the substitution groups of ligands are still veiled. A framework of those phosphorescent complexes often consists of three parts; i.e., 2-phenylpyridine-type (*C^N*) ligand, acetate-type (*O^O*) ligand, and a metal ion as illustrated in Figure 1 [10,11]. The heavy elements such as iridium and platinum ions are usually used for the metal center in the complexes to improve the efficiency of the phosphorescence by a spin-orbit coupling. On the other hand, it has been found that the *C^N* ligand directly contributes to both absorption and emission [12,13], while the *O^O* ligand increases solubility to organic solvents [10,14]. For this reason, if we can understand the contribution of the substituents of the *C^N* ligands, the absorption and emission colors as well as the efficiency of the phosphorescence can be controlled. In this regard, our group has already investigated a relationship between the molecular orbital (MO) of ligands and absorption/emission properties of a series of bis(dipyrrinato) zinc(II) complexes by time-dependent density functional theory (TD-DFT) calculations [15,16,17,18]. Those results have suggested the importance of energy level and distribution of π orbitals on ligands for tuning such optical properties. Therefore, in this paper we investigate relationships in the electronic structures of ligands and optical properties among several substituted *fac*-Ir(ppy)_3_ complexes by density functional theory (DFT) and time-dependent DFT calculations [19]. On the basis of those results, we present rational design guidelines for absorption and emission color tuning by modifying the species of substituents and their substitution positions.

## 2. Computational Details

**Figure 2:**
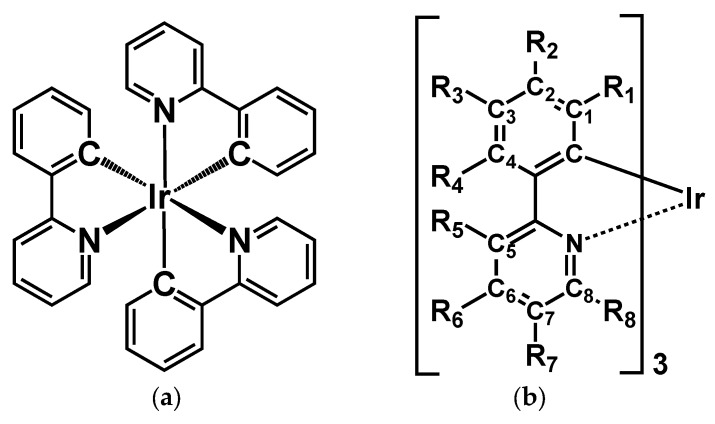
(**a**) Illustration of *fac*-Ir(ppy)_3_ complex (**1**). (**b**) R_2_–R_7_ represents possible substitution positions in complex **1***.* ppy: 2-phenylpyridine.

As illustrated in Figure 2, the *fac*-Ir(ppy)_3_ complex (**1**) has six possible substitution positions (R_2_–R_7_) in the ppy ligand. Note here that it is difficult to introduce substituents into R_1_ and R_8_ positions because of a steric repulsion. A variety of model complexes are constructed by introducing a given substituent chosen from six types of groups (i.e., -NH_2_ (**1**_*n*-**NH_2_**), -Cl (**1**_*n*-**Cl**), -OMe (**1**_*n*-**OMe**), -NO_2_ (**1**_*n*-**NO_2_**), -SO_2_Me (**1**_*n*-**SO_2_Me**), and -CN (**1**_*n*-**CN**) groups) into a given substitution position R*_n_* (*n* = 2–7). Those substituents are classified into electron-donating (-NH_2_, -Cl, and -OMe) and electron-withdrawing (-NO_2_, -SO_2_Me, and -CN) groups. At first, the geometries of each model complex were optimized at the B3LYP/**BS1** level of theory, where the **BS1** represents a set of LANL2DZ and 6-31G* basis sets for Ir atom and others, respectively. Assuming the optimized S_0_ geometry, their low-lying 30 excited states (S_1_–S_30_) are examined at the TD-B3LYP/**BS2** level of theory, where **BS2** represents a set of LANL08(f) and 6-31 + G* basis sets for Ir atom and others, respectively. In addition, their lowest triplet excited states (T_1_) were also optimized at the spin-unrestricted B3LYP/**BS1** level of theory, and the phosphorescence emission energies of the models were estimated by the energy gaps between T_1_ and S_0_ states at the T_1_ geometry. The Hessian of each optimized structure enabled confirmation they are all minima on the potential energy surface. All results were obtained under the assumption of a methanol solvent, which is approximated by the polarizable continuum model (PCM) using the integral equation formalism variant (IEFPCM) [20]. All calculations were performed by using Gaussian 09 program package [21].

Here we note that LANL2DZ and LANL08(f) partially approximates relativistic effect, but not enough for the consideration of the phosphorescence. In order to discuss the mechanism of the phosphorescence quantitatively, one must carry out a calculation including all-electrons explicit relativistic corrections using schemes such as second-order Douglas–Kress–Hall (HK2). However, due to the computational costs, we performed DFT and TD-DFT calculations with LANL2DZ and LANL08(f).

## 3. Results and Discussion

### 3.1. Unsubstituted Complex **1**

**Figure 3:**
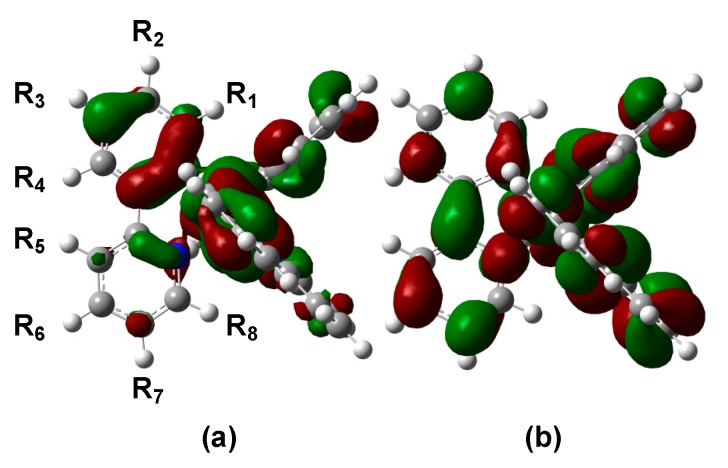
Frontier orbitals of *fac*-Ir(ppy)_3_: (**a**) highest occupied molecular orbital (HOMO) and (**b**) lowest unoccupied MO (LUMO).

The optimized ground state (S_0_) structures of complex **1** and its derivatives are summarized in Table S1 in the Supplementary Materials. Previous to examinations of the derivatives, the calculated results of the unsubstituted complex **1** are discussed in detail here. The highest occupied MO (HOMO) and lowest unoccupied MO (LUMO) of complex **1** are shown in Figure 3. As seen from Figure 2, the HOMO mainly consists of the π orbital of the phenyl ring mixed with the d orbital of the Ir ion, and is especially distributed at positions C_1_, C_3_, C_5_, and C_7_ in the ppy ligand (see Figure 2 for notation of carbon atoms). On the other hand, the LUMO is found to be dominantly distributed on the pyridine ring, especially at the substitution positions C_2_, C_4_, C_6_, and C_7_. The calculated Hirshfeld charges of carbon atoms in the ppy ligand of complex **1** are listed in Table 1, where the charges of hydrogen atoms are involved into their bonded carbon atoms. Since the formal charge of each ppy ligand is −1, all carbon atoms show negative atomic charges (see Table 1). The result indicates the existence of a weak charge density wave (CDW)-like structure on those carbon atoms; for example, in the phenyl ring, C_3_ is more negative in comparison with C_2_ and C_4_, while in the pyridine ring, C_5_ and C_7_ are more negative in comparison with C_6_. The origin of the CDW-like structure is explained by so-called ortho-para orientation, which stems from the nitrogen atom in the pyridine ring, and by a charge polarization on the phenyl ring induced by the pyridine ring. In addition, the charge densities of carbon atoms in the phenyl ring tend to be more negative than those of the pyridine ring because the nitrogen atom—which has a larger electronegativity—tends to withdraw negative charge from the carbon atoms in the pyridine ring.

**Figure 4:**
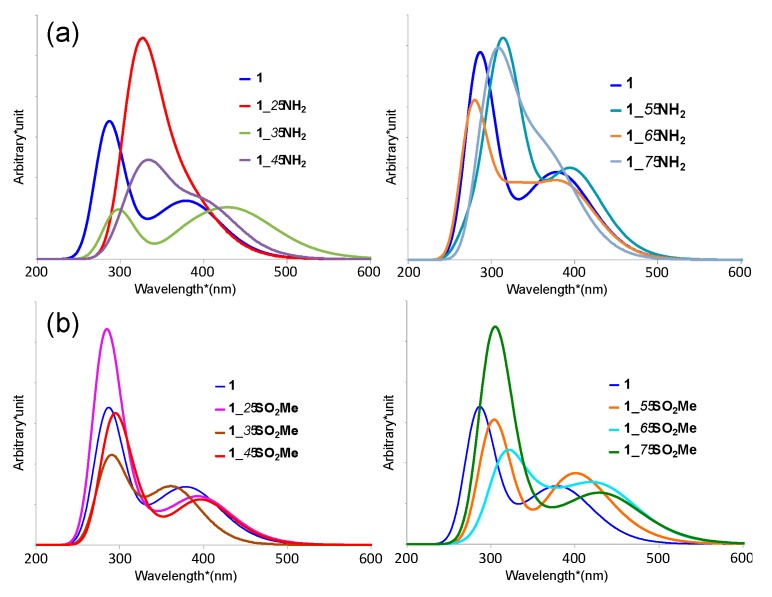
(**a**) Calculated absorption spectra of **1_***n***-NH_2_** in several substitution positions (left *n* = 2–4, right *n* = 5–7); (**b**) Calculated absorption spectra of **1_***n***-SO_2_Me** (left *n* = 2–4, right *n* = 5–7). All spectra are shown with the peak half-width of 0.300 eV.

The simulated absorption spectrum of the unsubstituted complex **1** is shown in Figure 4 within a range of 250–550 nm. The spectrum has two dominant peaks at 290 and 380 nm, and satisfactorily reproduces the experimental result that has peaks at 283 and 380 nm. As summarized in Table S2 in the Supplementary Information, the sharp peak around 290 nm is classified into a transition from π (HOMO − 4, HOMO − 5) orbitals to π^*^(LUMO + 1, LUMO + 2) orbitals of cyclometalated *C^N* ligands, indicating a ligand-to-ligand charge transfer (LLCT). On the other hand, the lower broad peaks in the visible light region (350–450nm) are transitions from (HOMO, HOMO − 1, HOMO − 2) to (LUMO, LUMO + 1, LUMO + 2) that can be attributed to a metal-to-ligand charge transfer (MLCT) or a metal-ligand-to-ligand charge transfer (MLLCT), which is a transition from the widely delocalized π orbital mixed with Ir orbital to π orbital of *C^N* ligands [11,19,22,23].

In order to examine the reliability of the DFT functional set, we confirm the absorption energy by using CAM-B3LYP. The calculated values for the LLCT and MLCT peaks that are 254 and 318/342 nm, respectively, fairly correspond to the B3LYP result (287 and 362/387 nm).

### 3.2. Absorption Spectra of Derivatives of Complex **1**

Next, the absorption spectra of the derivatives of complex **1** are examined. In order to simplify the discussion, we focus on amino- (**1**_*n*-**NH_2_**) and sulfonyl- (**1**_*n*-**SO_2_Me**) substitutes as representative of the electron-donating and -withdrawing groups, respectively. The results are summarized in Table 2 and shown in Figure 4. The detailed features of transition are given in Table S3 in the Supplementary Information. The substitutes of other electron-donating groups (**1**_*n*-**Cl** and **1**_*n*-**OMe**) and other electron-withdrawing groups (**1**_*n*-**NO_2_** and **1**_*n*-**CN**) show the same tendency as **1**_*n*-**NH_2_** and **1**_*n*-**SO_2_Me**, as shown in Figure S1.

Their spectral shapes are shown to be similar to the unsubstituted one, while the sharp LLCT peaks of the amino- and sulfonyl-substituents show red-shift in comparison with that of the unsubstituted complex **1**, except for **1**_*6*-**NH_2_**, and **1**_*2*-**SO_2_Me** (see Table 2). The broad MLCT peaks of the amino- and sulfonyl-substitutes also show red-shift in comparison with complex **1**, while **1**_*3*-**SO_2_Me**—in which the sulfonyl group is introduced in R_3_ position—only exhibits a blue-shift. Therefore, when focusing on the visible light region, **1**_*3*-**SO_2_Me** is the only derivative that exhibits a blue-shift.

From the results, we find a relationship between substitution groups, substitution positions, and peak shifts. For example, when the electron-donating groups are introduced in the R_3_ position, the MLCT absorption peaks are shown to be significantly red-shifted. Similarly, when the electron-withdrawing groups are introduced in the even-numbered positions on the ppy ligands (R_2_, R_4_, and R_6_), the MLCT absorption peaks also tend to be red-shifted.

### 3.3. Emission Wavelengths

We also estimate the emission wavelengths by S_0_-T_1_ energy gaps of complex **1** and its amino- and sulfonyl-substitutes at their optimized T_1_ structures as summarized in Table 3. The optimized structural data of the T_1_ state are given in Table S1 in the Supplementary Information. The S_0_-T_1_ energy gaps of other substitutes are given in Table S4 in the Supplementary Information. The emission originates in the one-electron transition from the LUMO to HOMO, and the calculated wavelength of complex **1** (532 nm) is found to be consistent with the experimental one (509 nm in CH_2_Cl_2_) [24]. All the calculated emission wavelengths are shown to be larger than the lower MLCT absorption band by about 100–200 nm, indicating the large Stokes shift. From the table, we also find the relationship between substitution groups, substitution positions, and peak shifts similar to the absorption spectra. For example, when the amino group is introduced to the odd-numbered positions on the ppy ligands (R_3_, R_5_, and R_7_), the emission wavelengths are shown to be significantly red-shifted in comparison with that of complex **1**. Especially, introduction of the amino group to R_3_ position exhibits a drastic shift. On the contrary, when the sulfonyl group is introduced to the even-numbered positions on the ppy ligands (R_2_, R_4_, and R_6_), the emission wavelengths are shown to be more red-shifted.

By summarizing the results in Table 2 and Table 3, we can, at this stage, deduce (i) that in the case of introducing the electron-donating groups into the R_3_ position on the ppy ligands, both the absorption and emission wavelengths tend to be red-shifted in comparison with that of complex **1**; (ii) that in the case of introducing the electron-withdrawing groups into the even-numbered positions on the ppy ligands (R_2_, R_4_ and R_6_), both the absorption peak and emission wavelengths tend to be red-shifted; and (iii) that in the case of introducing the electron-donating group into R_6_ position, or in the case of introducing an electron-withdrawing group into R_3_ position, the emission wavelength tends to be blue-shifted.

**Figure 5:**
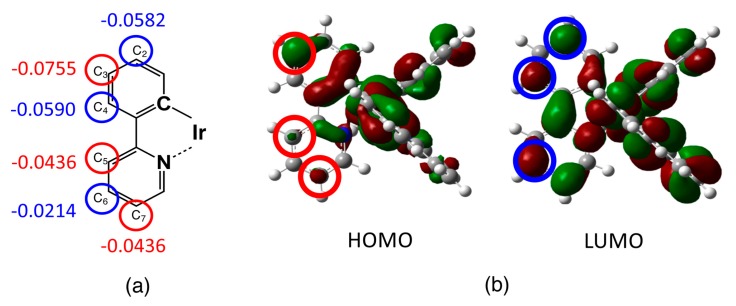
A relationship between (**a**) the distribution of Hirshfeld charge of ppy ligands in *fac*-Ir(ppy)_3_ and (**b**) the HOMO and LUMO orbitals in *fac*-Ir(ppy)_3_.

In order to elucidate the mechanism of such phenomena, we consider a relationship between the red/blue shift, charge density distribution on ligands, and frontier orbitals. First, let us recall the CDW-like structure on the ppy ligand of the unsubstituted complex **1**. Figure 5a illustrates the CDW-like structure on the ppy ligand; that is, C_3_, C_5_, and C_7_ atoms show bottoms of the wave (i.e., more negative), while C_2_, C_4_, and C_6_ atoms peaks of the wave (i.e., less negative), which are indicated by red and blue circles, respectively. In comparison with the results of the red/blue shift of the absorption and emission spectra, we can see that the red-shift of absorption and emission spectra is likely to be significant when introducing the electron-donating groups into the bottom (more negative carbon) positions, and/or when introducing the electron-withdrawing groups into the peak positions of the CDW-like structure.

This feature is understood by considering the distribution of the HOMO and LUMO as illustrated in Figure 5b. One can find that that the HOMO and LUMO are dominantly distributed around the carbon atoms at the bottoms and peaks of the CDW, respectively. The more negatively-charged carbon atoms in the ppy ligand tend to be included in the HOMO, so that the HOMO is predicted to be unstabilized if the electron-donor group is attached to those carbon atoms (indicated by red circles in Figure 5b). Similarly, the LUMO is predicted to be stabilized if the electron-withdrawing group is attached to the carbon atoms indicated by the blue circles in the figure. In such cases, the absorption and emission spectra are predicted to be red-shifted because the HOMO–LUMO gap is decreased. On the other hand, one cannot find a significant effect when the electron-donating and electron-withdrawing group are attached to (C_2_, C_4_, C_6_) and (C_3_, C_5_) positions, respectively. This feature is also explained based on the “nodes” in the HOMO and LUMO. For example, HOMO and LUMO have nodes at (C_2_, C_4_, C_6_) and (C_3_, C_5_) positions, respectively, which tend to attenuate the substituent effect at those “node” positions.

In order to demonstrate the above hypothesis, we summarize the HOMO–LUMO gap of complex **1** and the derivatives at S_0_ state in Table 4. Those HOMO and LUMO are also shown in Figure S2 in the Supplementary Information. The HOMO and LUMO energies of the unsubstituted complex **1** are shown to be −5.343 and −1.685 eV, respectively, and the gap is 3.658 eV. In the case of the amino-substituted models, the HOMO is found to be unstabilized by the substitution, suggesting a red-shift of the HOMO–LUMO transition. The HOMO–LUMO gaps of the models are shown to lie in a range of 3.6–3.7 eV, except for **1_***3***-NH_3_**, which exhibits a drastic red-shift in emission wavelength. The HOMO level of **1_***3***-NH_3_** model is shown to be significantly unstabilized from the unsbstituted one (c.a. 0.44 eV) in comparison with others (c.a. 0.1–0.2 eV), because the distribution of HOMO at the C_3_ position is shown to be considerably larger than other carbon atoms. We also find the unstabilization in the LUMO of **1_***2***-NH_3_**, **1_***6***-NH_3_**, and **1_***7***-NH_3_** (c.a. 0.2 eV) that is also predicted to originate in the larger distribution of LUMO at C_2_, C_6_, and C_7_ positions. Within those C_2_, C_6_, and C_7_, only C_6_ position has the “node” in HOMO, so that the introduction of the electron-donating group at this position is not predicted to contribute to the unstabilization of HOMO. As a result, the introduction of the electron-donating group into the C_6_ position is found to cause an increase in the HOMO–LUMO gap, resulting in a blue-shift in the emission of **1_***6***-NH_3_**.

On the other hand, in the case of the sulfonyl-substituted models, the frontier orbitals—especially the LUMO—are found to be stabilized in all models, indicating a decrease of the HOMO–LUMO gap (red-shift). The HOMO–LUMO gaps are shown to lie in the range of 3.3–3.6 eV, while **1_***3***-SO_2_Me** is found to have a larger gap than that of complex **1**. The results in Table 3 indicate that the LUMO of **1_***3***-SO_2_Me** is not as stabilized in comparison with others. Figure 5 clearly indicates that there is a “node” on C_3_ in the LUMO but it is not in the HOMO, so that it is predicted that the stabilization effect of the sulfonyl group at the C_3_ position only contributes to the HOMO but not to the LUMO. As a consequence, **1_***3***-SO_2_Me** exhibits a blue-shift in the emission. Those tendencies are qualitatively confirmed by other derivatives (**1**_*n*-**Cl**, **1**_*n*-**OMe**, **1**_*n*-**NO_2_**, **1**_*n*-**SO_2_Me**, and **1**_*n*-**CN**), as summarized in Figure S3.

On the basis of these results, we can conclude that most of the substitutions contribute to the red-shift, while the introduction of the electron-donating group into the C_6_ position or the introduction of the electron-withdrawing group into the C_3_ position have the potential to realize the blue-shift in this type of complex [13,25]. To date, modification or extension of π orbitals of the ppy ligand has mainly been discussed, but unfortunately there are no completely identical complexes to those we have proposed. However, for example, Aoki and co-workers reported that some complexes having the electron-withdrawing group at C_3_ position exhibit a blue-shift of emission [26]. In addition, a complex with fluorine substituents at C_2_ and C_4_ positions, where the LUMO dominantly populates, also exhibits a blue-shift of emission [27]. It is known that the fluorine acts as a donor for π orbitals by π back-donation; therefore, the fluorine atoms contribute to destabilizing the LUMO. Those experimental results indicate that one can certainly control the emission wavelength by introducing electron-donating or -withdrawing substituents to the ppy ligand.

On the other hand, in order to elucidate the solvation effect, we performed the calculation under the consideration of other solvents (CH_2_Cl_2_ and toluene) within the IEFPCM method. The estimated emission wavelengths of complex **1** (533 and 535 nm for CH_2_Cl_2_ and toluene, respectively) are almost the same as the result in MeOH (535 nm). Therefore, it is considered that the solvent effect does not contribute to the drastic change in the red/blue-shifts.

## 4. Concluding Remarks

In this paper, we investigated the optical properties of *fac*-Ir(ppy)_3_ derivatives. From the detailed analysis, we have clarified three fundamental relationships between red/blue-shift, electron-donating/withdrawing group, and electronic structures: (i) the electron-donating group—which mainly unstabilizes HOMO—effectively works for the red-shift of the emission (and the MLCT absorption) wavelength, especially when it is attached to the C_3_ position (where the HOMO is largely distributed); (ii) the electron-withdrawing group, which stabilizes LUMO rather than HOMO, also contributes to the red-shift of the emission wavelength—except for C_3_ position (where a node exists in the LUMO, but not in the HOMO); (iii) the blue-shift is possibly achieved when introducing the electron-donating group into the C_6_ position or introducing the electron-withdrawing group into the C_3_ position. These results straightforwardly provide rational design guidelines for absorption and emission color tuning of *fac*-Ir(ppy)_3_-based phosphorescent materials by modifying the species of substituents and their substitution positions.

## Figures and Tables

**Table 1:** Hirshfeld charges ^1^ of each carbon atom in the ppy ligand of the unsubstituted *fac*-Ir(ppy)_3_, where C_2_–C_4_ and C_5_–C_6_ belong to phenyl and pyridine rings, respectively.

Atoms ^2^	Phenyl Ring			Pyridine Ring		
	C_2_	C_3_	C_4_	C_5_	C_6_	C_7_
Hirshfeld charges ^3^	−0.0582	−0.0755	−0.0590	−0.0436	−0.0214	−0.0472
	(↗)	(↘)	(↗)	(↘)	(↗)	(↘)

^1^ Averaged values. ^2^ The number of carbon atoms is illustrated in Figure 2. ^3^ (↗) and (↘) represent the increase (more positive) and decrease (more negative) of Hirshfeld charges in comparison with adjacent carbon atoms.

**Table 2:** Calculated absorption wavelengths of the amino- and sulfonyl-substituted *fac*-Ir(ppy)_3_.

Complex	Absorption Wavelength (λ_ab_) (nm)		∆λ_ab_ (nm) ^1^		
	LLCT Peak	MLCT Peak	LLCT Peak	MLCT Peak	
**1**	287	(a) 362, (b) 387	-	(a)	(b)
**1**_*2*-**NH_2_**	320	386, 415	33	24	28
**1**_*3*-**NH_2_**	296	405, 439	9	43	52
**1**_*4*-**NH_2_**	322	393, 413	35	31	26
**1**_*5*-**NH_2_**	319	383, 398	32	21	11
**1**_*6*-**NH_2_**	276	381, 402	–11	19	15
**1**_*7*-**NH_2_**	307, 360	380, 391	20, 73	18	4
**1**_*2*-**SO_2_Me**	284	391, 409	–3	29	21
**1**_*3*-**SO_2_Me**	294	364, 376	7	2	–11
**1**_*4*-**SO_2_Me**	291	397, 420	4	35	33
**1**_*5*-**SO_2_Me**	304	393, 419	14	29	32
**1**_*6*-**SO_2_Me**	318	386, 431	31	24	44
**1**_*7*-**SO_2_Me**	304	427	17	40	-

^1^ A difference in λ_ab_ in comparison with the complex **1**. The positive and negative values indicate red and blue shifts, respectively. LLCT: ligand-to-ligand charge transfer; MLCT: metal-to-ligand charge transfer.

**Table 3:** Calculated emission wavelengths of the amino- and sulfonyl-substituted *fac*-Ir(ppy)_3_.

Complexes	Emission Wavelength (λ_em_, nm)	∆λ_em_ (nm) ^1^
**1**	532	
**1**_*2*-**NH_2_**	533	1
**1**_*3*-**NH_2_**	698	166
**1**_*4*-**NH_2_**	533	1
**1**_*5*-**NH_2_**	591	59
**1**_*6*-**NH_2_**	520	−12
**1**_*7*-**NH_2_**	575	43
**1**_*2*-**SO_2_Me**	566	34
**1**_*3*-**SO_2_Me**	514	−18
**1**_*4*-**SO_2_Me**	592	60
**1**_*5*-**SO_2_Me**	575	43
**1**_*6*-**SO_2_Me**	605	73
**1**_*7*-**SO_2_Me**	574	42

^1^ A difference in λ_em_ in comparison with complex **1**. The positive and negative values indicate red and blue shifts, respectively.

**Table 4:** HOMO, LUMO, and HOMO–LUMO gap energies of amino- and sulfonyl-substituted Ir(III) complexes.

Complexes	HOMO (eV)	LUMO (eV)	HOMO–LUMO Gap (eV)
**1**	−5.343	−1.685	3.658
**1**_*2*-**NH_2_**	−5.130	−1.435	3.695
**1**_*3*-**NH_2_**	−4.903	−1.635	3.267
**1**_*4*-**NH_2_**	−5.218	−1.654	3.564
**1**_*5*-**NH_2_**	−5.225	−1.564	3.661
**1**_*6*-**NH_2_**	−5.113	−1.438	3.675
**1**_*7*-**NH_2_**	−5.132	−1.425	3.706
**1**_*2*-**SO_2_Me**	−5.776	−2.185	3.590
**1**_*3*-**SO_2_Me**	−5.857	−2.001	3.856
**1**_*4*-**SO_2_Me**	−5.766	−2.094	3.672
**1**_*5*-**SO_2_Me**	−5.620	−2.104	3.516
**1**_*6*-**SO_2_Me**	−5.647	−2.342	3.304
**1**_*7*-**SO_2_Me**	−5.687	−2.320	3.367

## Supplementary Materials

The supplementary materials are available online.
