# Supplementary material for: Quantum Chemical Design Guidelines for Absorption and Emission Color Tuning of fac-Ir(ppy)3 Complexes
Source: Molecules. 2018 Mar 5;23(3):577. doi: 10.3390/molecules23030577 (PMC6017301; doi:10.3390/molecules23030577)
Supplement: Supplementary file 1 [file molecules-23-00577-s001.pdf]

Supplementary Information for

Quantum chemical design guidelines for absorption and emission color tuning of *fac*-Ir(ppy)$_3$ complexes

Yoshiki Natori[1)], Yasutaka Kitagawa[* 1,2)], Shogo Aoki[1)], Rena Teramoto[1)], Hayato Tada[1)], Iori Era[1)] and Masayoshi Nakano[* 1,2)]

1) Department of Materials Engineering Science, Graduate School of Engineering Science, Osaka University, Toyonaka, Osaka 560-8531, Japan.

2) Center for Spintronics Research Network (CSRN), Graduate School of Engineering Science, Osaka University, Toyonaka, Osaka 560-8531, Japan.

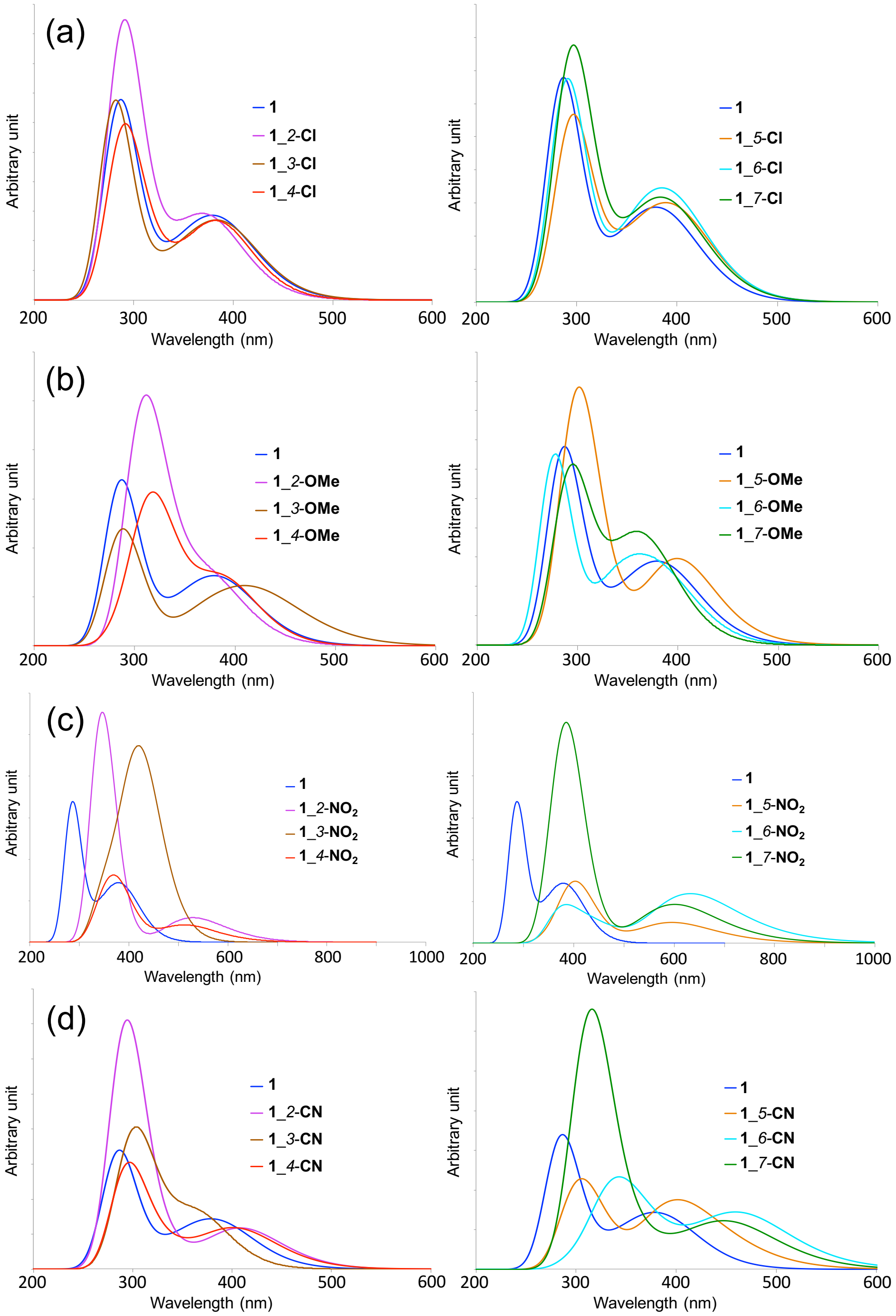

Figure S1. (a) Calculated absorption spectra of chloro-substituted *fac*-Ir(ppy)$_3$ in several substitution positions that are depicted with the peak half-width of 0.300 eV; (b) Calculated absorption spectra of methoxy-substituted *fac*-Ir(ppy)$_3$ in several substitution positions that are depicted with the peak half-width of 0.300 eV; (c) Calculated absorption spectra of nitro-substituted *fac*-Ir(ppy)$_3$ in several substitution positions that are depicted with the peak half-width of 0.300 eV; (d) Calculated absorption spectra of cyano-substituted *fac*-Ir(ppy)$_3$ in several substitution positions that are depicted with the peak half-width of 0.300 eV.

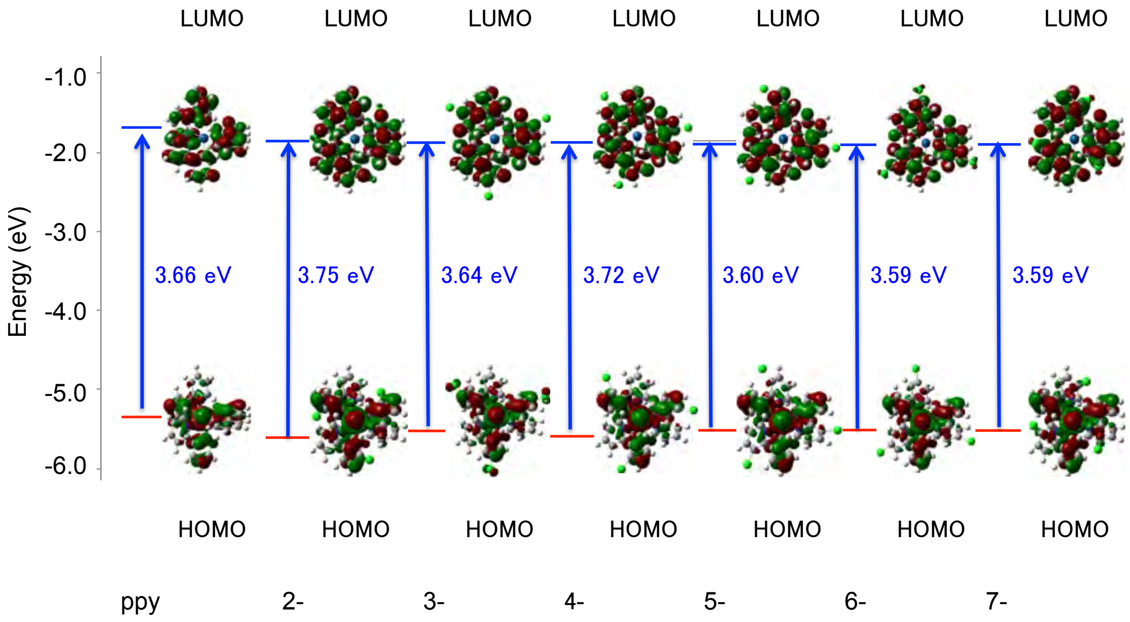

(a)

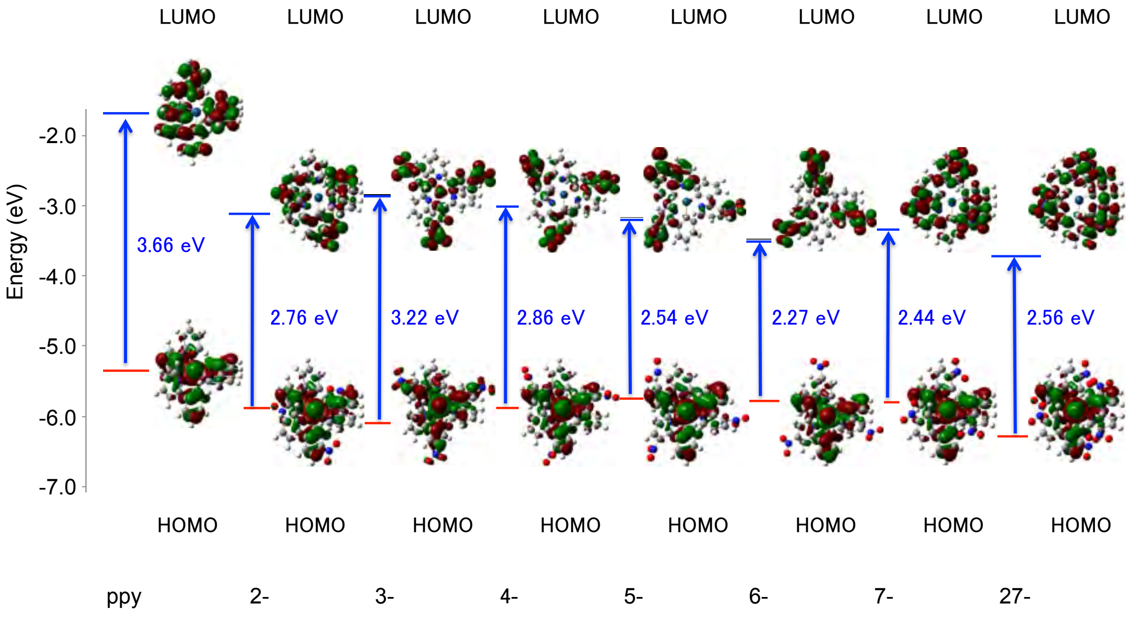

(b)

Figure S2. HOMO-LUMO (H-L) band gaps of (a) the chloro-substituted and (b) nitro-substituted Ir(III) complexes.

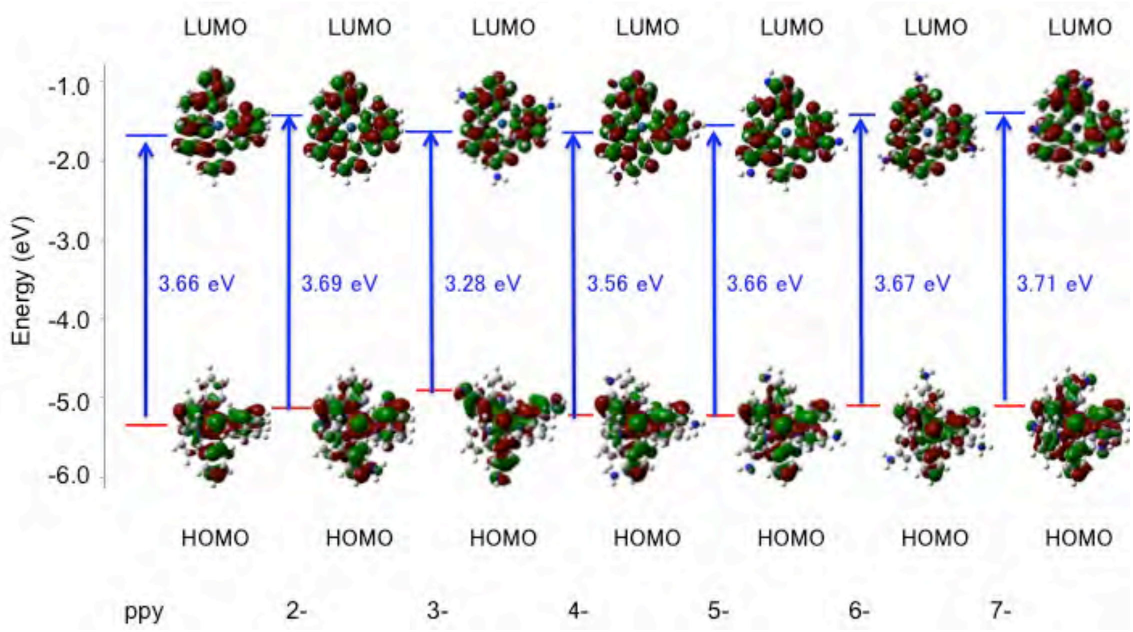
LUMO
-1.0
-2.0
-3.0
-4.0
-5.0
-6.0
Energy (eV)
3.66 eV
3.69 eV
3.28 eV
3.56 eV
3.66 eV
3.67 eV
3.71 eV
HOMO
ppy
2-
3-
4-
5-
6-
7-

(a)

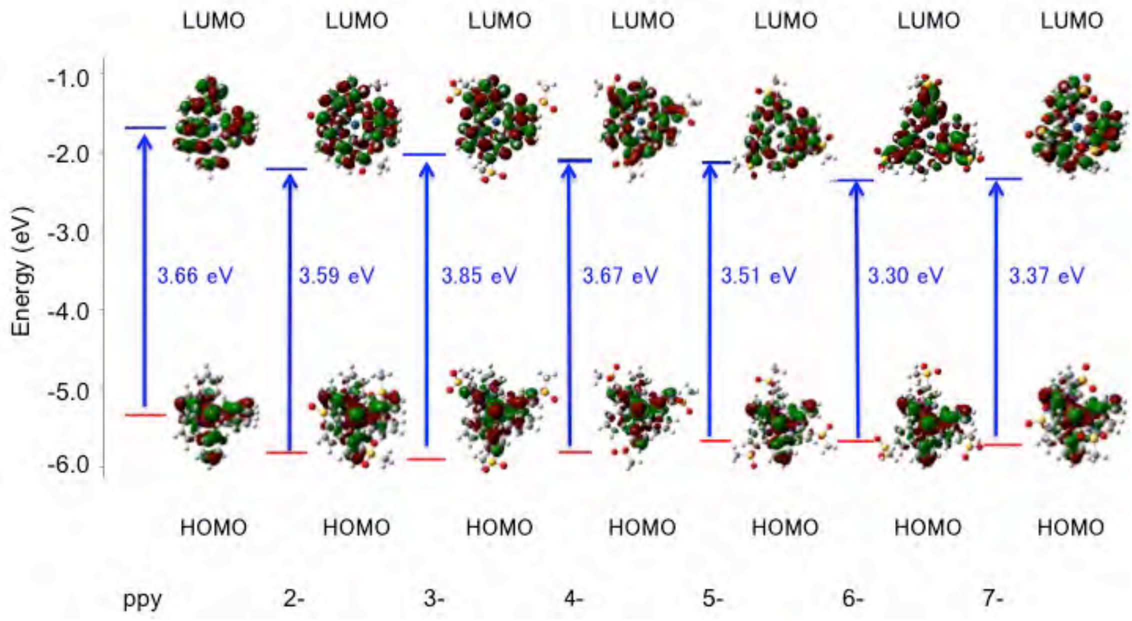
LUMO
-1.0
-2.0
-3.0
-4.0
-5.0
-6.0
Energy (eV)
3.66 eV
3.59 eV
3.85 eV
3.67 eV
3.51 eV
3.30 eV
3.37 eV
HOMO
ppy
2-
3-
4-
5-
6-
7-

(b)

(continue)

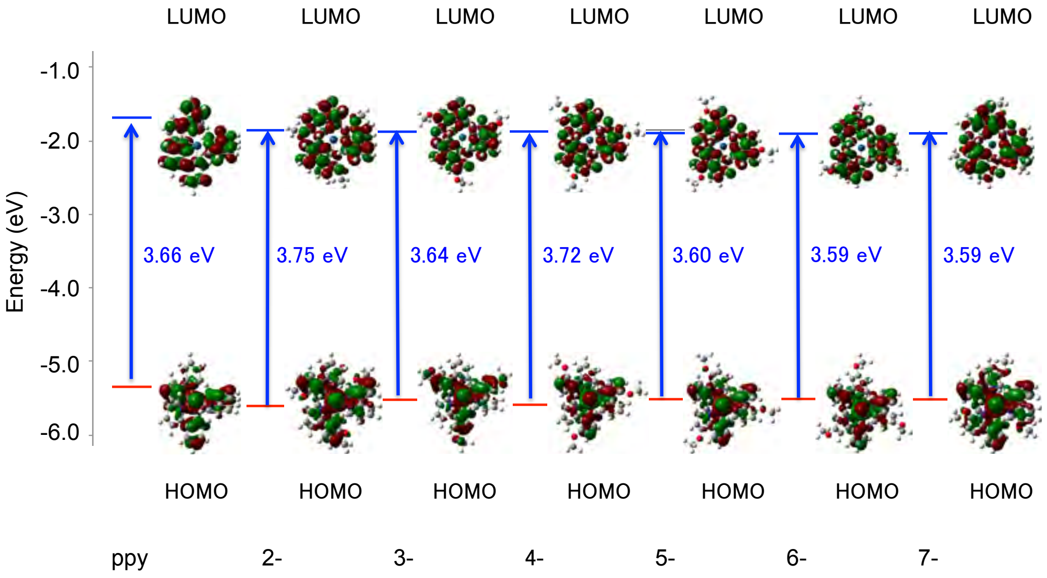

(c)

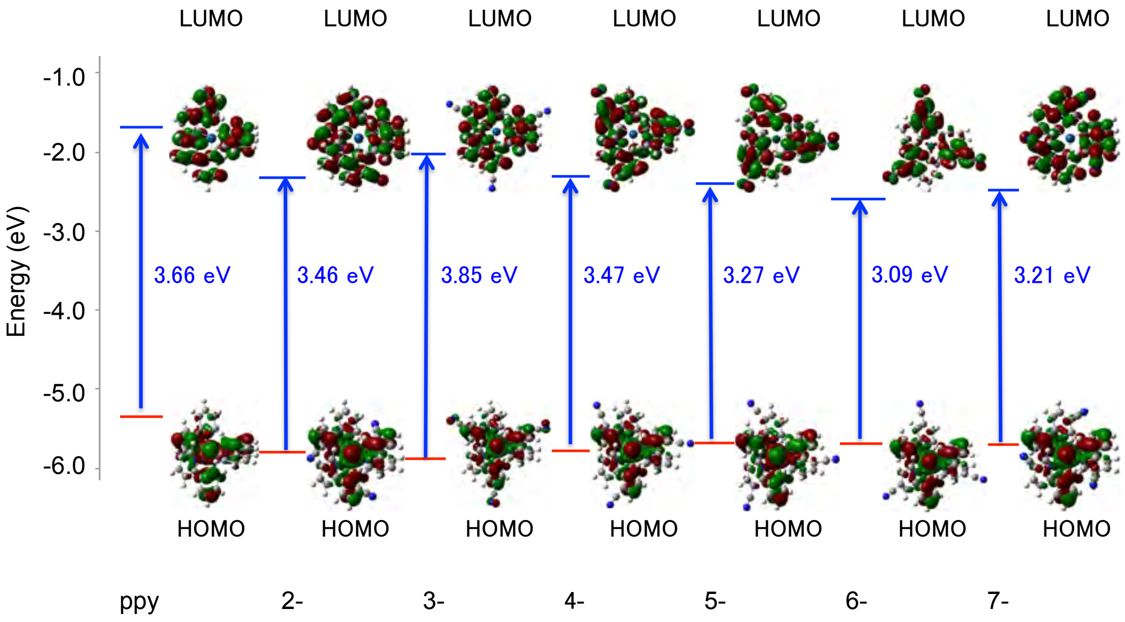

(d)

Figure S3. HOMO-LUMO (H-L) band gaps of (a) chloro-substituted, (b) nitro-substituted, (c) methoxy-substituted and (d) the cyano-substituted Ir(III) complexes.

Table S1 (1). Optimized parameter of S_0 state of **1**.

Atoms	X	Y	Z
Ir	-0.019334	-0.005291	0.033814
C	-2.040328	0.021783	0.215377
C	-3.003154	0.000533	-0.814371
C	-2.5451541	0.053978	1.55083799
C	-4.3745899	0.010976	-0.552111
H	-2.6688559	-0.020582	-1.847798
C	-3.928257	0.06165	1.81179404
C	-4.8454442	0.04097	0.76629698
H	-5.082674	-0.004572	-1.378472
H	-4.2975259	0.083887	2.83362007
H	-5.9120178	0.046744	0.97413099
C	-1.5470001	0.084169	2.6290679
C	-1.832088	0.139872	4.00367594
C	0.74813098	0.08448	3.1095109
C	-0.797647	0.16560701	4.93092823
H	-2.860873	0.16515	4.34325314
C	0.52473301	0.136943	4.47952318
H	1.75288796	0.063638	2.70208693
H	-1.018904	0.209033	5.99322081
N	-0.250007	0.056131	2.20859408
H	1.36350298	0.157019	5.1666832
C	0.013064	-0.251347	-1.980101
C	0.066308	0.74621397	-2.975076
C	-0.011337	-1.602543	-2.442019
C	0.091954	0.439605	-4.3370872
H	0.082352	1.79015696	-2.6748509
C	0.016791	-1.908167	-3.8156591
C	0.067825	-0.893256	-4.7654719
H	0.130888	1.24248302	-5.070807
H	-0.000949	-2.941246	-4.1526799
H	0.089592	-1.135847	-5.8244782
C	-0.07258	-2.64801	-1.410791
C	-0.127513	-4.0307941	-1.653543
C	-0.134331	-3.054956	0.897677
C	-0.184159	-4.9244919	-0.591148
H	-0.128343	-4.4023771	-2.6714301
C	-0.187157	-4.4311619	0.71656698
H	-0.138072	-2.6157229	1.88923597
H	-0.227091	-5.9931221	-0.77951
N	-0.076342	-2.186547	-0.127556
H	-0.231811	-5.0911069	1.57598805
C	0.225199	2.00862694	0.009486
C	1.576195	2.47184491	0.014032
C	-0.774243	3.00317502	-0.003005
C	1.87976897	3.84625697	0.008237
C	-0.469549	4.36579609	-0.011305
H	-1.817935	2.70173502	-0.011651
C	0.86311603	4.79544592	-0.005207
H	2.91264009	4.18437719	0.013397
H	-1.273731	5.09903193	-0.022768
H	1.10416603	5.85501909	-0.009532
C	2.6237731	1.44101095	0.017548
C	4.00747919	1.68409395	-0.001112
C	3.03380299	-0.86764	0.039333
C	4.90311384	0.62184799	0.003
H	4.37836695	2.70202494	-0.021211
C	4.41088009	-0.686113	0.024336
H	2.59555411	-1.8595591	0.052476
H	5.97245789	0.81046301	-0.011926
H	5.07241106	-1.545473	0.026704
N	2.16340995	0.157555	0.038432

Table S1(2). Optimized parameter of T_1 state of **1**.

Atoms	X	Y	Z
Ir	-0.060276	0.006647	0.029795
C	-2.0392699	0.043546	0.178242
C	-2.9921589	0.007185	-0.85691
C	-2.541708	0.102828	1.57725
C	-4.3637981	0.046157	-0.612016
H	-2.6407039	-0.037329	-1.8834961
C	-3.958678	0.151131	1.79700899
C	-4.831008	0.121985	0.73361498
H	-5.0765538	0.027163	-1.431392
H	-4.3525429	0.20890699	2.80722904
H	-5.9017882	0.157001	0.92286903
C	-1.583365	0.088321	2.61037207
C	-1.854808	0.123607	4.01706219
C	0.74264699	0.070527	3.09429312
C	-0.833878	0.138045	4.93151522
H	-2.8861091	0.136802	4.3545022
C	0.51691699	0.119173	4.46491909
H	1.75533605	0.050253	2.701864
H	-1.046141	0.163954	5.99620581
N	-0.235276	0.038625	2.17986107
H	1.35606205	0.13897	5.15119219
C	-0.00652	-0.26172	-1.993255
C	0.048592	0.72560197	-2.9941571
C	-0.00985	-1.618619	-2.432395
C	0.090771	0.40262401	-4.3526502
H	0.050311	1.77298105	-2.704953
C	0.033865	-1.940553	-3.801806
C	0.082753	-0.935534	-4.7625508
H	0.12835801	1.196033	-5.0962262
H	0.029914	-2.977047	-4.1277609
H	0.115432	-1.191213	-5.8180642
C	-0.055097	-2.6551681	-1.388742
C	-0.07954	-4.041214	-1.617004
C	-0.108969	-3.041513	0.928877
C	-0.119647	-4.9232249	-0.544038
H	-0.069059	-4.4246678	-2.630156
C	-0.134394	-4.4194059	0.76014102
H	-0.120381	-2.584887	1.91262996
H	-0.139775	-5.9942741	-0.721782
N	-0.070442	-2.187603	-0.108437
H	-0.167007	-5.0734901	1.62435699
C	0.21145	2.01582909	0.036943
C	1.56397903	2.46729803	0.034132
C	-0.788455	3.00758696	0.06258
C	1.86923695	3.84059	0.05871
C	-0.478828	4.36863804	0.082511
H	-1.831679	2.70588112	0.055263
C	0.85504103	4.79210615	0.08158
H	2.90257597	4.17568684	0.061409
H	-1.280759	5.10356379	0.098422
H	1.09998298	5.85040998	0.09907
C	2.60998011	1.43476903	0.005192
C	3.99278092	1.67809999	-0.035505
C	3.01586294	-0.8741	-0.027908
C	4.88570881	0.614196	-0.070683
H	4.36506319	2.695467	-0.043021
C	4.3924408	-0.693375	-0.067264
H	2.57708597	-1.865633	-0.024928
H	5.95476723	0.80163503	-0.102569
H	5.05274296	-1.553035	-0.095366
N	2.15017605	0.153183	0.011609

Table S1(3). Optimized parameter of S_0 state of **1**_*2*-**NH$_2$**.

Atoms	X	Y	Z
Ir	-0.065796	-0.018633	0.036277
C	-2.0871489	-0.001965	0.22731601
C	-3.0465009	-0.025807	-0.794675
C	-2.5754859	0.020869	1.57353604
C	-4.430912	-0.024352	-0.540065
H	-2.7190001	-0.039968	-1.8318141
C	-3.959909	0.017516	1.82994699
C	-4.8833509	-0.00396	0.79629701
H	-4.3322349	0.028429	2.85131311
H	-5.9497938	-0.005014	1.00891101
C	-1.577423	0.051416	2.638906
C	-1.85157	0.097822	4.01986122
C	0.72328901	0.065818	3.10889101
C	-0.812277	0.12582999	4.93885422
H	-2.8785729	0.114318	4.36624718
C	0.50991797	0.109466	4.48008299
H	1.72542596	0.054464	2.6939249
H	-1.02738	0.16179	6.00298405
N	-0.279353	0.034336	2.21272898
H	1.35250902	0.13218901	5.16251183
N	-5.3466992	0.016802	-1.5914
H	-4.9990411	-0.342793	-2.473012
H	-6.2640472	-0.357976	-1.378096
C	-0.043224	-0.26295	-1.979044
C	-0.005408	0.73298103	-2.9650259
C	-0.054686	-1.62189	-2.43154
C	0.01662	0.44166699	-4.3419781
H	-0.000484	1.77866697	-2.6655819
C	-0.028022	-1.914963	-3.8084049
C	0.006756	-0.906317	-4.7587199
H	-0.030903	-2.9458239	-4.1539331
H	0.025814	-1.147373	-5.818924
C	-0.099587	-2.660593	-1.4061691
C	-0.139024	-4.0484042	-1.644732
C	-0.151088	-3.0699141	0.90559202
C	-0.182315	-4.9398069	-0.582166
H	-0.138445	-4.4212732	-2.662487
C	-0.188417	-4.446311	0.72763801
H	-0.157535	-2.628608	1.89653504
H	-0.212899	-6.0093098	-0.76966
N	-0.104946	-2.200475	-0.119689
H	-0.223595	-5.1063972	1.58742595
N	-0.012359	1.46802998	-5.2861228
H	0.33997601	2.35958004	-4.9568749
H	0.37696901	1.23106003	-6.1915331
C	0.166394	1.99789906	0.011316
C	1.522457	2.45912004	0.018183
C	-0.836214	2.97783303	0.000753
C	1.80632699	3.83819008	0.015861
C	-0.553959	4.35681486	-0.006084
H	-1.879876	2.67150402	-0.009338
C	0.79130501	4.78224087	0.003072
H	2.83483005	4.19046688	0.02685
C	2.5681951	1.43997598	0.020943
C	3.95505905	1.68667197	0.005497
C	2.992625	-0.869577	0.034661
C	4.85364294	0.62930697	0.007174
H	4.32177591	2.70654297	-0.010501
C	4.3684001	-0.683482	0.022285
H	2.55777192	-1.86334	0.043281
H	5.92238998	0.82310301	-0.005064
H	5.03432178	-1.539482	0.022011
N	2.11609912	0.15072399	0.037169
H	1.02534497	5.84418201	0.000222
N	-1.585374	5.29287481	-0.081344
H	-2.482075	4.96645117	0.26040599
H	-1.362594	6.20943785	0.28983799

Table S1(4). Optimized parameter of T_1 state of **1**_*2*-**NH$_2$**.

Atoms	X	Y	Z
Ir	-0.062289	-0.003931	0.032843
C	-2.0737059	0.010975	0.197055
C	-3.02495	-0.015401	-0.804603
C	-2.561254	0.032541	1.59813094
C	-4.4248409	-0.014359	-0.543859
H	-2.705889	-0.031886	-1.84421
C	-3.9848969	0.047019	1.84727204
C	-4.88065	0.025802	0.80736601
H	-4.3605151	0.074943	2.86491489
H	-5.9501972	0.039035	1.00599205
C	-1.594821	0.028898	2.62349296
C	-1.8601871	0.036513	4.03203487
C	0.73196203	0.04931	3.09254098
C	-0.838734	0.054545	4.94610786
H	-2.8903911	0.026016	4.37346792
C	0.51090401	0.068515	4.46877718
H	1.74477696	0.05514	2.69919205
H	-1.046344	0.059077	6.01180506
N	-0.242386	0.018286	2.17958307
H	1.35450494	0.091713	5.14975405
N	-5.336237	0.006503	-1.577462
H	-5.0124788	-0.275081	-2.494556
H	-6.2906842	-0.266199	-1.378073
C	-0.036819	-0.267418	-1.98818
C	0.012925	0.718108	-2.983228
C	-0.058545	-1.630692	-2.4261811
C	0.036606	0.41372401	-4.3576369
H	0.025911	1.76686001	-2.693886
C	-0.031077	-1.9371901	-3.800206
C	0.015355	-0.938107	-4.7603569
H	-0.041834	-2.9710259	-4.1365252
H	0.035098	-1.190079	-5.818028
C	-0.106144	-2.6624351	-1.3919261
C	-0.15318	-4.0518699	-1.621438
C	-0.142425	-3.057621	0.92397898
C	-0.194458	-4.9356952	-0.552461
H	-0.159647	-4.4320111	-2.636425
C	-0.188773	-4.4347949	0.75477898
H	-0.137925	-2.6055081	1.91026604
H	-0.231658	-6.0061989	-0.733102
N	-0.101234	-2.196964	-0.108083
H	-0.221344	-5.09021	1.61824799
N	0.019417	1.43103194	-5.3116279
H	0.37993899	2.32229996	-4.9905548
H	0.40748	1.18143797	-6.2141781
C	0.163757	2.00921893	0.009431
C	1.51829696	2.47468305	0.018395
C	-0.843891	2.98384809	0.003157
C	1.79550898	3.85482907	0.024615
C	-0.567346	4.36386013	0.005748
H	-1.885739	2.67175603	-0.012413
C	0.776196	4.794415	0.017555
H	2.82240391	4.2113862	0.038618
C	2.56792998	1.45974004	0.015864
C	3.95376801	1.71157205	-0.00279
C	2.99975801	-0.848391	0.011804
C	4.85557318	0.65704799	-0.012308
H	4.31705809	2.73273802	-0.01281
C	4.37479877	-0.657475	-0.005664
H	2.56792092	-1.843448	0.015955
H	5.92362785	0.85437298	-0.026963
H	5.04361582	-1.511128	-0.015114
N	2.12079096	0.16927201	0.026425
H	1.00585306	5.85726881	0.020932
N	-1.602636	5.29603481	-0.062699
H	-2.4976659	4.9636631	0.27770799
H	-1.383255	6.2107482	0.31505999

Table S1(5). Optimized parameter of S_0 state of **1**_*3*-**NH$_2$**.

Atoms	X	Y	Z
Ir	-0.04272	0.010806	0.01373
C	-2.0675991	0.037992	0.194259
C	-3.0376239	0.018231	-0.827626
C	-2.5790241	0.068205	1.52365804
C	-4.407836	0.030281	-0.568475
H	-2.711576	-0.006026	-1.8638591
C	-3.9617779	0.075172	1.79085505
C	-4.894526	0.058646	0.75157899
H	-4.3292561	0.095444	2.81420994
C	-1.583716	0.094822	2.60438299
C	-1.8714941	0.14678501	3.97894192
C	0.70999902	0.09728	3.091115
C	-0.839835	0.17129099	4.90937805
H	-2.901135	0.170496	4.31613493
C	0.48368001	0.14584599	4.46112299
H	1.71597302	0.079344	2.686234
H	-1.064081	0.21163	5.97125006
N	-0.28466	0.069362	2.18665695
H	1.32092202	0.16551501	5.15027809
H	-5.1171489	0.017707	-1.395062
N	-6.2797632	0.13824099	1.00928104
H	-6.8428931	-0.301927	0.28843701
H	-6.5396962	-0.242157	1.91389596
C	-0.009402	-0.233463	-2.0042291
C	0.045208	0.756338	-3.0057499
C	-0.034093	-1.578577	-2.473412
C	0.068651	0.45344701	-4.3668609
H	0.066992	1.80265105	-2.7133019
C	-0.005923	-1.889823	-3.8465879
C	0.043361	-0.881364	-4.8114452
H	-0.023573	-2.9241149	-4.1823978
C	-0.092814	-2.626962	-1.445257
C	-0.145176	-4.0096479	-1.691213
C	-0.156221	-3.041224	0.86172998
C	-0.200856	-4.9070692	-0.631868
H	-0.14501	-4.3784242	-2.710155
C	-0.206228	-4.417408	0.67721099
H	-0.162217	-2.6049869	1.85472298
H	-0.241487	-5.9752698	-0.823625
N	-0.098398	-2.1686959	-0.159749
H	-0.250615	-5.0797372	1.53490996
H	0.106528	1.25677705	-5.1015282
N	-0.001326	-1.183699	-6.1891961
H	0.46112299	-0.486023	-6.7635241
H	0.376663	-2.0999889	-6.408711
C	0.20187999	2.02854896	-0.009106
C	1.54723799	2.49774408	-0.002668
C	-0.788873	3.03060794	-0.022865
C	1.85771501	3.87141991	-0.007414
C	-0.486534	4.39196777	-0.033005
H	-1.835392	2.73811197	-0.028562
C	0.84850401	4.83655119	-0.02529
H	2.892097	4.20735312	-0.000028
C	2.59670496	1.46912205	0.003941
C	3.98046088	1.71426404	-0.010689
C	3.01195908	-0.838493	0.021405
C	4.87880898	0.65426999	-0.006418
H	4.3494072	2.73298192	-0.028188
C	4.38909817	-0.654705	0.010385
H	2.57585001	-1.831524	0.03066
H	5.94783401	0.84537703	-0.018129
H	5.05221891	-1.5129401	0.012214
N	2.13839197	0.183741	0.021055
H	-1.290431	5.12684822	-0.047758
N	1.15260303	6.21238279	-0.103877
H	0.439206	6.8007822	0.314823
H	2.05509901	6.44491911	0.298695

Table S1(6). Optimized parameter of T_1 state of **1**_*3*-**NH$_2$**.

Atoms	X	Y	Z
Ir	-0.040762	0.054382	0.012631
C	-2.0752599	0.080357	0.206328
C	-3.0529561	0.090059	-0.806392
C	-2.5725279	0.073706	1.54092205
C	-4.4212961	0.097729	-0.535063
H	-2.7361209	0.094703	-1.84604
C	-3.9525271	0.078397	1.82097101
C	-4.89503	0.092922	0.78974003
H	-4.3115392	0.074204	2.84738493
C	-1.569392	0.058543	2.61698699
C	-1.848257	0.056621	3.99467611
C	0.73114699	0.026941	3.09059596
C	-0.809512	0.040415	4.91739702
H	-2.8750651	0.068768	4.34056997
C	0.51234299	0.025527	4.4623909
H	1.73122895	0.016295	2.67068291
H	-1.027366	0.039976	5.98137522
N	-0.272081	0.042707	2.19657111
H	1.35229194	0.015069	5.14830923
H	-5.1381421	0.109196	-1.355005
N	-6.2762742	0.170706	1.06326401
H	-6.8500562	-0.243433	0.335574
H	-6.5303788	-0.231701	1.95986605
C	-0.002025	-0.204409	-2.003331
C	0.060649	0.786928	-3.001935
C	-0.027099	-1.548835	-2.4699609
C	0.092121	0.48506999	-4.3626461
H	0.082353	1.832636	-2.7073231
C	0.008135	-1.857586	-3.843462
C	0.065574	-0.849306	-4.8079662
H	-0.009704	-2.8915329	-4.1793628
C	-0.092072	-2.599715	-1.445027
C	-0.142811	-3.981611	-1.696285
C	-0.173748	-3.0204189	0.85942101
C	-0.207106	-4.8815198	-0.63983
H	-0.133831	-4.3477469	-2.7159939
C	-0.223216	-4.395977	0.67063701
H	-0.185624	-2.5881081	1.853863
H	-0.245834	-5.949141	-0.834978
N	-0.106916	-2.146627	-0.159525
H	-0.27406	-5.0608101	1.52593195
H	0.137651	1.28941905	-5.0954289
N	0.0281	-1.150995	-6.1849742
H	0.49362001	-0.454262	-6.7578158
H	0.40256301	-2.0688	-6.4037809
C	0.180819	2.02267289	-0.021983
C	1.59435904	2.48937297	-0.013459
C	-0.826185	3.01284003	-0.053749
C	1.86108601	3.87360907	-0.037894
C	-0.556709	4.37501621	-0.079113
H	-1.863063	2.69096303	-0.066268
C	0.81371099	4.81146383	-0.071756
H	2.88129592	4.24831009	-0.031204
C	2.60095406	1.48559701	0.028066
C	4.01148796	1.70867002	0.044291
C	3.010602	-0.849265	0.080898
C	4.89281082	0.65480202	0.078125
H	4.38706684	2.72733688	0.028037
C	4.38722897	-0.677889	0.094503
H	2.57613397	-1.8454011	0.092681
H	5.96432781	0.83627802	0.089994
H	5.04531097	-1.539585	0.11711
N	2.13001895	0.163909	0.057888
H	-1.351428	5.11508512	-0.105478
N	1.07456601	6.14015579	-0.096601
H	0.32800701	6.8199172	-0.122925
H	2.02165198	6.49245405	-0.095803

Table S1(7). Optimized parameter of S_0 state of **1_*4*-NH$_2$**.

Atoms	X	Y	Z
Ir	-0.014463	-0.021496	0.045458
C	-2.0397911	-0.004903	0.21720199
C	-2.9578121	-0.051403	-0.846433
C	-2.5658369	0.080217	1.54948199
C	-4.3335018	0.024483	-0.62393
H	-2.589978	-0.13527	-1.86412
C	-3.9659779	0.24947099	1.76294899
C	-4.8350511	0.196196	0.66337103
H	-5.9040222	0.31179601	0.82962197
C	-1.571769	-0.016126	2.62961507
C	-1.830938	-0.199083	4.00422192
C	0.73536998	-0.058048	3.09618092
C	-0.785193	-0.283404	4.91642809
H	-2.8489699	-0.317388	4.34657717
C	0.53148198	-0.18703	4.46395302
H	1.73558402	-0.018138	2.679389
H	-0.99975	-0.427543	5.97141886
N	-0.269974	0.007324	2.20671391
H	1.378052	-0.231445	5.14032316
H	-5.025207	-0.015448	-1.463607
N	-4.5293121	0.44655699	3.04046106
H	-4.0223479	1.11335504	3.61442494
H	-5.5000172	0.740291	2.98153591
C	0.027818	-0.2572	-1.973015
C	0.097211	0.77556503	-2.92417
C	-0.039767	-1.60632	-2.457505
C	0.061284	0.50778502	-4.2933831
H	0.167238	1.80517304	-2.588074
C	-0.167776	-1.866408	-3.854176
C	-0.092202	-0.795821	-4.7571302
H	-0.176805	-0.997169	-5.8229508
C	0.02906	-2.653388	-1.426258
C	0.222167	-4.0352249	-1.634388
C	0.003795	-3.0436211	0.89551401
C	0.277913	-4.912106	-0.556946
H	0.37042499	-4.4105792	-2.6367331
C	0.142287	-4.4168811	0.74079102
H	-0.066385	-2.593214	1.87939703
H	0.43046901	-5.9731422	-0.732274
N	-0.034297	-2.1882479	-0.140295
H	0.163681	-5.0648761	1.61013198
H	0.118409	1.32457495	-5.0107999
N	-0.345275	-3.162461	-4.3805981
H	-1.0253071	-3.721441	-3.8744669
H	-0.610908	-3.1363361	-5.360764
C	0.22135501	1.99761605	0.033547
C	1.57221305	2.48024988	-0.007475
C	-0.812371	2.95024395	0.042222
C	1.83703399	3.87232804	-0.170938
C	-0.542691	4.31778193	-0.028221
H	-1.843928	2.61642098	0.091543
C	0.76526898	4.77695704	-0.156395
C	2.61627889	1.45228398	0.125025
C	3.99123502	1.66747403	0.35600999
C	3.00697899	-0.868728	0.18130299
C	4.86590099	0.59300101	0.47155899
H	4.36139917	2.674196	0.486976
C	4.37515402	-0.708603	0.35868201
H	2.55950904	-1.85497	0.12683301
H	5.92150402	0.77368599	0.65286398
H	5.02209091	-1.576335	0.426651
N	2.15321398	0.16467801	0.085017
H	0.96984297	5.83968592	-0.268072
H	-1.360364	5.03640413	-0.018242
N	3.13815403	4.39317703	-0.325574
H	3.71844506	3.86437511	-0.969525
H	3.12100101	5.36377287	-0.624796

Table S1(8). Optimized parameter of T_1 state of **1_*4*-NH₂**.

Atoms	X	Y	Z
Ir	-0.017179	-0.006155	0.023129
C	-2.031455	-0.034205	0.20182399
C	-2.935801	-0.09614	-0.872272
C	-2.5588479	0.03603	1.53332996
C	-4.3138518	-0.056857	-0.655678
H	-2.55532	-0.154353	-1.886694
C	-3.96348	0.164958	1.73811698
C	-4.8233099	0.092402	0.63038498
H	-5.8956852	0.17763899	0.79148799
C	-1.565738	-0.031904	2.61666608
C	-1.824139	-0.218979	3.99024296
C	0.73994499	0.022665	3.09130597
C	-0.77874	-0.254026	4.90616989
H	-2.837498	-0.378117	4.32969522
C	0.53472298	-0.103391	4.45903301
H	1.73931098	0.098573	2.67772007
H	-0.991159	-0.401763	5.96095419
N	-0.264489	0.038207	2.19998193
H	1.37888205	-0.107439	5.1395998
H	-5.000196	-0.107649	-1.498453
N	-4.5401011	0.33860299	3.00966311
H	-4.0561709	1.01067197	3.59676194
H	-5.5197129	0.599922	2.94975901
C	0.054395	-0.227448	-1.9699171
C	0.239438	0.77358598	-2.928541
C	-0.069022	-1.6263	-2.436218
C	0.209041	0.495482	-4.2983279
H	0.38030601	1.79882205	-2.6007321
C	-0.24592	-1.863864	-3.8629561
C	-0.07118	-0.815639	-4.7482972
H	-0.182344	-1.00687	-5.8143601
C	0.027487	-2.6309271	-1.44587
C	0.193923	-4.0448389	-1.632089
C	-0.008883	-3.0090671	0.91550702
C	0.218797	-4.9080119	-0.568612
H	0.33711499	-4.4176951	-2.6385291
C	0.073263	-4.3817539	0.75761098
H	-0.074878	-2.571619	1.90719795
H	0.36250699	-5.9727049	-0.725459
N	0.010112	-2.1327729	-0.104175
H	0.057149	-5.0281401	1.62825298
H	0.33490601	1.29233599	-5.0269108
N	-0.548219	-3.154995	-4.3430581
H	-1.246073	-3.6361871	-3.7819009
H	-0.8557	-3.1387341	-5.3116322
C	0.232069	2.02372694	0.037551
C	1.58359802	2.49745393	-0.012876
C	-0.800727	2.97289109	0.079974
C	1.85004795	3.89087605	-0.16013
C	-0.527813	4.34140205	0.025149
H	-1.831918	2.63928008	0.139192
C	0.77911502	4.79682207	-0.116883
C	2.62639093	1.46602201	0.112289
C	4.00174904	1.67862105	0.34316501
C	3.01357198	-0.859255	0.163175
C	4.87319899	0.60118097	0.45402101
H	4.37445021	2.6840539	0.47674999
C	4.38166714	-0.700497	0.338541
H	2.55660701	-1.8413431	0.108251
H	5.92922211	0.77950299	0.63491702
H	5.02897787	-1.568036	0.40348601
N	2.16594911	0.178444	0.068842
H	0.98494601	5.86041498	-0.216029
H	-1.342616	5.0621891	0.056965
N	3.1484549	4.40997124	-0.323881
H	3.72394896	3.88710093	-0.97665
H	3.13373995	5.38515091	-0.607394

Table S1(9). Optimized parameter of S_0 state of **1_*5*-NH₂**.

Atoms	X	Y	Z
Ir	0.017075	-0.051879	0.079581
C	-2.0040851	-0.118596	0.20572799
C	-2.9132881	-0.263844	-0.859767
C	-2.557071	-0.092831	1.52770603
C	-4.2869582	-0.418384	-0.657921
H	-2.530009	-0.269753	-1.876547
C	-3.9397781	-0.298495	1.72341502
C	-4.801888	-0.458144	0.64001101
H	-4.3483529	-0.385367	2.722754
H	-5.8630829	-0.61781	0.81249797
C	-1.5907021	0.085659	2.63082504
C	-1.885191	0.37694901	3.99486899
C	0.71700603	0.029659	3.13631511
C	-0.827081	0.40160501	4.91578197
C	0.48171699	0.205613	4.4940362
H	1.71840596	-0.067148	2.73382211
H	-1.045002	0.59741098	5.96278
N	-0.286834	-0.019591	2.24722695
H	1.30785894	0.226319	5.19674921
H	-4.9526658	-0.52702	-1.512187
N	-3.184227	0.61110902	4.45189381
H	-3.2066319	1.04739904	5.36801481
H	-3.7709789	1.12735403	3.80580902
C	0.141431	-0.24297	-1.93241
C	0.30474001	0.79271299	-2.8723471
C	0.13315199	-1.582738	-2.4422159
C	0.493155	0.546664	-4.2345262
H	0.29737201	1.82144201	-2.5226669
C	0.373541	-1.822552	-3.812299
C	0.55032098	-0.767232	-4.7053609
H	0.47409701	-2.8341911	-4.1858058
H	0.73672301	-0.973755	-5.756062
C	-0.069079	-2.6539731	-1.444914
C	-0.344604	-4.0291362	-1.700436
C	-0.080464	-3.079479	0.87997001
C	-0.397342	-4.9129582	-0.61211
C	-0.242965	-4.445466	0.68669599
H	-0.014936	-2.6410439	1.86873698
H	-0.581893	-5.967917	-0.799317
N	-0.005665	-2.2254691	-0.152369
H	-0.285409	-5.1194081	1.53565598
H	0.61474198	1.37911999	-4.925139
N	-0.536152	-4.5324121	-2.9893291
H	-0.963551	-5.452971	-2.9930439
H	-1.040611	-3.91188	-3.6127739
C	0.21006501	1.96365094	0.15093701
C	1.55101299	2.47031903	0.164774
C	-0.826775	2.90996909	0.26051301
C	1.78808105	3.84639692	0.37110701
C	-0.582228	4.27659702	0.41599199
H	-1.856015	2.56242609	0.236683
C	0.73096502	4.74642992	0.493469
H	2.79769206	4.22056913	0.488134
C	2.62495589	1.46539795	0.025261
C	4.00845623	1.70974302	-0.216709
C	3.04487801	-0.859035	0.104081
C	4.89093781	0.61912102	-0.205118
C	4.41572714	-0.673078	-0.021786
H	2.60273004	-1.844674	0.18907399
H	5.95168877	0.798522	-0.362194
H	5.08867502	-1.5238529	-0.01404
N	2.19135189	0.17634401	0.117591
H	0.93540198	5.80178595	0.65409398
H	-1.415761	4.97200108	0.4957
N	4.52042103	2.99075508	-0.436253
H	5.45246601	2.97886801	-0.837869
H	3.9158721	3.59855795	-0.977813

Table S1(10). Optimized parameter of T_1 state of **1_5-NH$_2$**.

Atoms	X	Y	Z
Ir	0.002192	-0.038325	0.071619
C	-2.0179789	-0.090189	0.18287601
C	-2.9157391	-0.2197	-0.89388
C	-2.582484	-0.071866	1.49955499
C	-4.291255	-0.37321	-0.707001
H	-2.5220721	-0.210537	-1.906445
C	-3.9673719	-0.277534	1.67840695
C	-4.8186989	-0.426259	0.58528203
H	-4.385756	-0.372745	2.67277408
H	-5.8817301	-0.585583	0.74579
C	-1.6290571	0.100863	2.61458993
C	-1.939075	0.38273901	3.97727799
C	0.673554	0.052321	3.14233398
C	-0.88971	0.40645301	4.90840816
C	0.42401701	0.21978	4.4987092
H	1.67944801	-0.039186	2.74996209
H	-1.118874	0.59417099	5.95444107
N	-0.322026	0.002874	2.24470401
H	1.24297905	0.24002799	5.2097168
H	-4.9483619	-0.470155	-1.569088
N	-3.243078	0.60740799	4.42332602
H	-3.2767749	1.03255296	5.34430408
H	-3.8259301	1.12820005	3.7774229
C	0.117128	-0.224345	-1.914312
C	0.202408	0.79981297	-2.870219
C	0.106235	-1.621418	-2.4154451
C	0.31551301	0.55075198	-4.2408509
H	0.188015	1.82990801	-2.5250909
C	0.31255901	-1.8447241	-3.8281109
C	0.39658901	-0.788154	-4.7041869
H	0.450813	-2.852581	-4.2023749
H	0.55624503	-0.982659	-5.7624469
C	-0.079501	-2.631273	-1.458626
C	-0.339332	-4.0573192	-1.687289
C	0.138918	-3.0917709	0.86881697
C	-0.181724	-4.9600291	-0.642352
C	0.110063	-4.4765062	0.65319699
H	0.25543499	-2.692333	1.87284398
H	-0.366592	-6.0170012	-0.813762
N	0.012375	-2.193908	-0.10143
H	0.219644	-5.153523	1.49289095
H	0.38245699	1.37382996	-4.9476829
N	-0.736723	-4.490417	-2.9265411
H	-1.080749	-5.4411378	-2.9898541
H	-1.220335	-3.837085	-3.5276339
C	0.23006199	1.98164797	0.13676
C	1.57817495	2.4640851	0.17566399
C	-0.792216	2.94406605	0.232666
C	1.83550501	3.83492398	0.39227101
C	-0.527727	4.30591917	0.399391
H	-1.8267421	2.61417699	0.189101
C	0.79168999	4.75225878	0.50133502
H	2.84929895	4.19145203	0.52743101
C	2.64121604	1.44351494	0.052421
C	4.02917624	1.67207003	-0.179021
C	3.03600311	-0.885731	0.148524
C	4.89953709	0.57147801	-0.152475
C	4.41007805	-0.714605	0.034311
H	2.58015895	-1.864966	0.233013
H	5.96350622	0.73890799	-0.300792
H	5.07462788	-1.571756	0.052977
N	2.19559002	0.159229	0.148092
H	1.01145995	5.8031621	0.67059201
H	-1.3504471	5.01510286	0.46849599
N	4.55746698	2.94513893	-0.402429
H	5.49116898	2.92133403	-0.799536
H	3.96219397	3.55978489	-0.946463

Table S1(11). Optimized parameter of S_0 state of **1_*6*-NH₂**.

Atoms	X	Y	Z
Ir	-0.012104	-0.014422	0.047499
C	-2.0303941	0.018215	0.243062
C	-3.000653	-0.000924	-0.781646
C	-2.5312009	0.048114	1.58143198
C	-4.3711982	0.009386	-0.515136
H	-2.6701641	-0.020966	-1.816618
C	-3.9129579	0.055113	1.84621596
C	-4.8362818	0.03682	0.80523002
H	-4.2785382	0.074497	2.869735
C	-1.526683	0.07395	2.6594131
C	-1.81629	0.127838	4.02402878
C	0.757245	0.073176	3.13560605
C	-0.784978	0.150887	4.97853422
H	-2.846731	0.154053	4.36146498
C	0.54271901	0.122511	4.50050497
H	1.76561904	0.056862	2.73568296
N	-0.232459	0.045259	2.22465611
H	1.38466501	0.141471	5.18520212
H	-5.082664	-0.004658	-1.3389651
H	-5.9019842	0.042054	1.01856697
N	-1.057066	0.147614	6.32605982
H	-0.319647	0.45636499	6.94632912
H	-1.978035	0.453091	6.61338902
C	0.01611	-0.279094	-1.962744
C	0.068828	0.71104002	-2.9671249
C	-0.006861	-1.634015	-2.417619
C	0.095014	0.39737299	-4.3274279
H	0.084213	1.75709105	-2.6730621
C	0.022211	-1.946286	-3.7891259
C	0.072342	-0.938143	-4.7471099
H	0.006569	-2.9816229	-4.1202021
C	-0.064861	-2.67682	-1.377741
C	-0.120852	-4.0501499	-1.622768
C	-0.123296	-3.075464	0.92017102
C	-0.176177	-4.9690571	-0.560774
H	-0.124009	-4.4213138	-2.641824
C	-0.176209	-4.4464822	0.75040299
H	-0.129537	-2.6421061	1.91483796
N	-0.065679	-2.1986761	-0.098619
H	-0.219899	-5.1022439	1.61415601
H	0.133507	1.19605005	-5.0661368
H	0.094874	-1.188468	-5.8045111
N	-0.176888	-6.325151	-0.786808
H	-0.508362	-6.9174471	-0.036326
H	-0.463464	-6.6415501	-1.704317
C	0.247381	1.99592495	0.01423
C	1.601071	2.45500207	0.022576
C	-0.746743	2.9976511	-0.005251
C	1.90836596	3.82795906	0.014936
C	-0.437869	4.35921621	-0.016079
H	-1.791752	2.69979405	-0.016966
C	0.89649802	4.78307581	-0.005363
H	2.94271111	4.162395	0.024327
C	2.64798999	1.41778302	0.033735
C	4.02178812	1.66582894	0.015356
C	3.05417299	-0.87939	0.062153
C	4.94467306	0.605946	0.024845
H	4.39032888	2.68552995	-0.008555
C	4.42575598	-0.706457	0.049163
H	2.62382293	-1.8753181	0.073727
H	5.08485985	-1.568748	0.054823
N	2.17336106	0.13757101	0.058142
H	-1.2393481	5.09570217	-0.032754
H	1.14299798	5.84160089	-0.010973
N	6.29940701	0.83682698	0.062286
H	6.90438414	0.078704	-0.226113
H	6.62260008	1.74632895	-0.241701

Table S1(12). Optimized parameter of T_1 state of **1_*6*-NH_2**.

Atoms	X	Y	Z
Ir	-0.017225	0.054915	0.046577
C	-2.04614	0.072785	0.252891
C	-3.0182569	0.076926	-0.765072
C	-2.526643	0.062129	1.59556305
C	-4.3869462	0.075318	-0.484856
H	-2.6957531	0.087695	-1.802755
C	-3.905411	0.05936	1.87418199
C	-4.8370051	0.06727	0.84017599
H	-4.262425	0.052868	2.90055203
C	-1.509357	0.050854	2.66357088
C	-1.7831351	0.044571	4.03023386
C	0.78722799	0.037506	3.11514401
C	-0.738538	0.032369	4.97357321
H	-2.8091929	0.049446	4.38067007
C	0.58663797	0.030488	4.48043489
H	1.78840303	0.038098	2.69758105
N	-0.217023	0.04589	2.21787596
H	1.43426299	0.024405	5.15788698
H	-5.1066999	0.081765	-1.301023
H	-5.900434	0.065984	1.06385803
N	-0.992723	-0.024567	6.31595278
H	-0.247247	0.221497	6.95334721
H	-1.918522	0.221523	6.64006615
C	0.034568	-0.265425	-1.960636
C	0.11259	0.71455699	-2.9703569
C	0.005208	-1.62501	-2.388051
C	0.155348	0.381322	-4.32551
H	0.129356	1.76272094	-2.686482
C	0.052207	-1.953769	-3.7549019
C	0.125499	-0.959072	-4.724987
H	0.032897	-2.9927349	-4.0721541
C	-0.075403	-2.6550269	-1.336228
C	-0.142866	-4.028698	-1.5690939
C	-0.178013	-3.0263331	0.964257
C	-0.22702	-4.9355078	-0.497327
H	-0.133041	-4.4106612	-2.5837641
C	-0.244918	-4.3974881	0.80827999
H	-0.194619	-2.583458	1.95429099
N	-0.090182	-2.1636109	-0.064277
H	-0.310944	-5.0428162	1.67822897
H	0.21232601	1.16967595	-5.073276
H	0.16029	-1.224838	-5.7780218
N	-0.241365	-6.2902122	-0.707946
H	-0.574298	-6.87849	0.04456
H	-0.495496	-6.6211872	-1.6294791
C	0.207802	2.02011704	-0.031671
C	1.63314605	2.4617331	-0.060708
C	-0.785596	3.01448989	-0.052412
C	1.90986705	3.87062597	-0.124045
C	-0.485658	4.3790288	-0.109726
H	-1.826879	2.70512295	-0.034734
C	0.88066697	4.78523111	-0.147098
H	2.93620396	4.22472811	-0.152538
C	2.6302669	1.46544898	-0.003984
C	4.04677296	1.69293296	-0.018246
C	3.02732491	-0.857356	0.094251
C	4.93787909	0.63367498	0.026703
H	4.42475224	2.70936108	-0.061594
C	4.40943193	-0.691541	0.07973
H	2.59939694	-1.8551461	0.133871
H	5.06433678	-1.555553	0.116818
N	2.15239406	0.148459	0.06586
H	-1.275151	5.12469912	-0.130331
H	1.117015	5.84681416	-0.19423
N	6.31593609	0.82552201	0.084958
H	6.86708593	0.064659	-0.296082
H	6.63727903	1.72061002	-0.26623

Table S1(13). Optimized parameter of S_0 state of **1**_*7*-**NH$_2$**.

Atoms	X	Y	Z
Ir	-0.022785	-0.000577	0.033037
C	-2.04669	0.015208	0.204126
C	-3.0055301	-0.005559	-0.828181
C	-2.554421	0.039097	1.53873801
C	-4.3795481	-0.000838	-0.571906
H	-2.666482	-0.02162	-1.860464
C	-3.937408	0.041714	1.79394901
C	-4.8528481	0.022883	0.74454403
H	-4.3096361	0.059435	2.81529093
H	-5.920095	0.024964	0.95057398
C	-1.557284	0.064412	2.619977
C	-1.833364	0.100557	3.99651599
C	0.74034899	0.066087	3.10317802
C	-0.806109	0.119744	4.92654514
H	-2.860352	0.112314	4.34369516
C	0.53119802	0.103364	4.48932314
H	1.74644399	0.053723	2.69615602
H	-1.029762	0.150168	5.98964024
N	-0.260337	0.04756	2.2101779
N	1.59891403	0.18818	5.37564611
H	1.41098499	-0.161554	6.30805111
H	2.48182988	-0.162917	5.02275705
H	-5.0846992	-0.015644	-1.400997
C	0.019378	-0.236755	-1.98384
C	0.068352	0.76304299	-2.9755731
C	0.007878	-1.587569	-2.447803
C	0.100976	0.46165401	-4.3400312
H	0.07549	1.80620599	-2.6712761
C	0.042406	-1.888142	-3.821177
C	0.088181	-0.869646	-4.770009
H	0.032914	-2.9211071	-4.1604118
H	0.11475	-1.110988	-5.8295012
C	-0.046811	-2.635669	-1.416834
C	-0.08015	-4.0204501	-1.6489151
C	-0.115933	-3.043354	0.89442599
C	-0.13329	-4.9162688	-0.592786
H	-0.063903	-4.400506	-2.6640489
C	-0.155538	-4.43538	0.72953397
H	-0.130237	-2.6029849	1.88641596
H	-0.161232	-5.9861221	-0.782127
N	-0.064255	-2.183948	-0.133991
N	-0.279771	-5.2856822	1.822294
H	0.066969	-6.2265301	1.67588305
H	0.045936	-4.9070382	2.70424199
H	0.136191	1.26708901	-5.071373
C	0.206908	2.01719499	0.017729
C	1.55601597	2.48594093	0.031249
C	-0.796918	3.00609803	0.003814
C	1.85122705	3.86097002	0.030522
C	-0.500778	4.3720541	0.00116
H	-1.838919	2.69806695	-0.01027
C	0.82899803	4.80669689	0.01442
H	2.88294601	4.20391989	0.040642
C	2.60842299	1.45807099	0.038753
C	3.99275804	1.69513094	0.037081
C	3.02592611	-0.852451	0.05353
C	4.89330387	0.641877	0.041563
H	4.36872196	2.71189594	0.032573
C	4.41795397	-0.682584	0.047301
H	2.58986211	-1.846504	0.058447
H	5.9628129	0.83498597	0.036845
N	2.16170192	0.17339399	0.048344
H	1.06623805	5.86744881	0.013886
N	5.27620602	-1.774153	-0.019179
H	6.20526505	-1.6129659	0.352027
H	4.89155483	-2.6484499	0.31966501
H	-1.309106	5.10093784	-0.011754

Table S1(14). Optimized parameter of T_1 state of **1**_7-NH_2.

Atoms	X	Y	Z
Ir	-0.025524	0.008735	0.028052
C	-2.027293	0.02694	0.195134
C	-2.9832771	0.018095	-0.833909
C	-2.5351231	0.033988	1.57352495
C	-4.359087	0.02776	-0.584983
H	-2.6388111	0.011629	-1.86428
C	-3.95383	0.049058	1.80396795
C	-4.8371911	0.045207	0.74898499
H	-4.3392649	0.065706	2.81950998
H	-5.907846	0.055779	0.93983102
C	-1.582952	0.019132	2.60948491
C	-1.857984	0.025955	4.01169109
C	0.75760102	0.071194	3.101933
C	-0.864585	0.059494	4.94428205
H	-2.8925431	0.005716	4.33947992
C	0.51585299	0.098174	4.47586679
H	1.77974701	0.094236	2.73305798
H	-1.075212	0.07123	6.00867414
N	-0.210106	0.006927	2.17097306
N	1.53233695	0.214931	5.38954306
H	1.349069	-0.058837	6.34644222
H	2.48243904	0.04773	5.08225822
H	-5.0655441	0.025614	-1.41166
C	0.015471	-0.241671	-1.9974001
C	0.075778	0.74954998	-2.996124
C	-0.001524	-1.596266	-2.4493239
C	0.1113	0.43803301	-4.358448
H	0.088502	1.79522204	-2.6994419
C	0.035012	-1.907476	-3.8204539
C	0.09015	-0.896565	-4.7770591
H	0.020423	-2.9426341	-4.1526451
H	0.117862	-1.146829	-5.834465
C	-0.055456	-2.640604	-1.4120801
C	-0.08992	-4.0266151	-1.637961
C	-0.112025	-3.039187	0.90336299
C	-0.138262	-4.9171081	-0.57682
H	-0.077928	-4.412303	-2.6509759
C	-0.153694	-4.4319019	0.74427599
H	-0.11952	-2.5872469	1.89050603
H	-0.166942	-5.9877572	-0.761739
N	-0.066619	-2.1874721	-0.130337
N	-0.273146	-5.2783942	1.84015501
H	0.070898	-6.2205019	1.69562399
H	0.055902	-4.8969369	2.71959996
H	0.15449201	1.23715901	-5.0962119
C	0.21213201	2.02094102	0.025788
C	1.56229305	2.48629594	0.04568
C	-0.791792	3.00978303	0.01949
C	1.85743904	3.861233	0.062632
C	-0.494397	4.37478781	0.034062
H	-1.83339	2.70099211	-0.00511
C	0.83603001	4.80763388	0.056563
H	2.8892951	4.20315695	0.07829
C	2.61428404	1.45839202	0.038724
C	3.99887991	1.69442403	0.03784
C	3.02857494	-0.852371	0.006967
C	4.89771891	0.63991803	0.01901
H	4.37624502	2.71055007	0.052102
C	4.42087793	-0.684045	-0.001651
H	2.59081602	-1.84559	-0.004852
H	5.9674859	0.83166599	0.01585
N	2.16711497	0.17468099	0.026741
H	1.07418203	5.86804104	0.06853
N	5.27690506	-1.774912	-0.093719
H	6.20867014	-1.622913	0.27438
H	4.89276505	-2.655787	0.228195
H	-1.301983	5.1043601	0.026411

Table S1(15). Optimized parameter of S_0 state of **1**_*2*-**Cl**.

Atoms	X	Y	Z
Ir	-0.018723	-0.007556	0.035437
C	-2.037987	0.020508	0.21636499
C	-2.988955	-0.000821	-0.822254
C	-2.543397	0.057757	1.55066299
C	-4.3523779	0.013511	-0.540367
H	-2.6636479	-0.024904	-1.856398
C	-3.924953	0.069103	1.80643904
C	-4.8464799	0.047672	0.76455599
H	-4.302485	0.094727	2.82435298
H	-5.9131522	0.05604	0.95869797
C	-1.5451781	0.087738	2.62919903
C	-1.831125	0.148601	4.00225687
C	0.74987298	0.079956	3.10725904
C	-0.795734	0.17308301	4.92889214
H	-2.8595259	0.179076	4.34229994
C	0.52586401	0.137419	4.47700214
H	1.75440097	0.053359	2.70026112
H	-1.016328	0.220819	5.99095297
N	-0.249264	0.05347	2.20742989
H	1.36477602	0.155994	5.1638298
Cl	-5.5089159	-0.013343	-1.884357
C	0.013168	-0.252791	-1.9768521
C	0.063045	0.754098	-2.9603429
C	-0.011869	-1.602962	-2.43942
C	0.086729	0.42813501	-4.3137808
H	0.078535	1.79850602	-2.669179
C	0.014891	-1.9033901	-3.811729
C	0.064262	-0.89213	-4.7657762
H	-0.002408	-2.9329	-4.157177
H	0.085445	-1.121118	-5.8253059
C	-0.071952	-2.6487811	-1.408079
C	-0.127529	-4.030221	-1.651724
C	-0.13081	-3.0530491	0.90040398
C	-0.183444	-4.9232111	-0.588332
H	-0.129699	-4.402442	-2.669277
C	-0.18461	-4.4291668	0.71866298
H	-0.132694	-2.614049	1.891801
H	-0.227175	-5.9917388	-0.776038
N	-0.073893	-2.1858289	-0.125881
H	-0.228588	-5.0886951	1.57827902
Cl	0.14840899	1.73385096	-5.5120039
C	0.22573701	2.00475192	0.011097
C	1.57595003	2.46799588	0.011643
C	-0.782443	2.98826599	-0.000436
C	1.87503195	3.84088492	0.005096
C	-0.457693	4.34214687	-0.00923
H	-1.8267911	2.69650006	-0.007233
C	0.862535	4.79479885	-0.005806
H	2.90449405	4.18692684	0.0083
H	1.09052503	5.85474586	-0.01045
C	2.62345409	1.43667305	0.013936
C	4.00587082	1.68003201	-0.010375
C	3.02991796	-0.872001	0.041738
C	4.90038395	0.61647302	-0.006164
H	4.3778038	2.69737411	-0.035258
C	4.40696001	-0.69046	0.021436
H	2.59153509	-1.863503	0.060046
H	5.96966505	0.80396497	-0.025727
H	5.0677228	-1.550249	0.024456
N	2.1611619	0.154539	0.040099
Cl	-1.764948	5.54018688	-0.024872

Table S1(16). Optimized parameter of T_1 state of **1_*2*-Cl**.

Atoms	X	Y	Z
Ir	-0.025399	-0.000831	0.00962
C	-2.0433061	0.006694	0.205713
C	-2.9899421	-0.034875	-0.834257
C	-2.5427611	0.040027	1.54059696
C	-4.353601	-0.044337	-0.55214
H	-2.6612029	-0.049903	-1.867401
C	-3.9243491	0.026406	1.79522002
C	-4.8451648	-0.014984	0.75362098
H	-4.3018122	0.047602	2.81293511
H	-5.9115429	-0.023992	0.94910902
C	-1.54585	0.090074	2.61988902
C	-1.835053	0.158691	3.9916451
C	0.74852699	0.128397	3.09907389
C	-0.800508	0.21106599	4.9180479
H	-2.8638921	0.173967	4.33088112
C	0.52196503	0.196251	4.46785307
H	1.75380397	0.114667	2.69359803
H	-1.022719	0.264651	5.97941399
N	-0.250227	0.072901	2.2010541
H	1.35953796	0.236981	5.15519619
Cl	-5.5075359	-0.095633	-1.894051
C	-0.007095	-0.210593	-1.970398
C	0.05013	0.78789997	-2.9479699
C	-0.051934	-1.630394	-2.425586
C	0.051454	0.46785301	-4.3067899
H	0.082493	1.83040702	-2.651314
C	-0.058694	-1.906718	-3.8423519
C	-0.007324	-0.88619	-4.753685
H	-0.105619	-2.9292171	-4.201941
H	-0.011802	-1.09755	-5.8184738
C	-0.070504	-2.625423	-1.437475
C	-0.10456	-4.0434542	-1.666909
C	-0.116386	-3.031894	0.89971399
C	-0.149478	-4.9220872	-0.616137
H	-0.09306	-4.4143038	-2.685848
C	-0.162542	-4.413271	0.71468699
H	-0.120803	-2.6095691	1.90004396
H	-0.175319	-5.9928999	-0.793485
N	-0.059116	-2.152981	-0.103494
H	-0.204921	-5.0712881	1.57499301
Cl	0.115283	1.74746001	-5.517457
C	0.23716199	2.02010298	0.000594
C	1.59193301	2.46464205	0.010301
C	-0.761717	3.00970793	-0.004725
C	1.90505099	3.83453798	0.012255
C	-0.423734	4.36120176	-0.003195
H	-1.808833	2.727525	-0.017189
C	0.90148902	4.79796791	0.004582
H	2.93743706	4.17123508	0.019389
H	1.14091504	5.85538721	0.005803
C	2.631284	1.42304802	0.022368
C	4.01579523	1.65511	0.015679
C	3.02057505	-0.892425	0.061799
C	4.90057516	0.58346403	0.032154
H	4.39711523	2.66886806	-0.004376
C	4.39852285	-0.720668	0.056716
H	2.56886292	-1.878093	0.078997
H	5.97137403	0.76278698	0.025494
H	5.05397892	-1.5842789	0.069239
N	2.16358995	0.143158	0.045063
Cl	-1.71718	5.57148504	-0.013625

Table S1(17). Optimized parameter of S_0 state of **1**_*3*-**Cl**.

Atoms	X	Y	Z
Ir	-0.017888	-0.008913	0.036931
C	-2.0372901	0.019769	0.21677101
C	-2.9993391	-0.002027	-0.812892
C	-2.5420749	0.05662	1.55027699
C	-4.3721471	0.010706	-0.561744
H	-2.67027	-0.025724	-1.847325
C	-3.9226539	0.066797	1.81842303
C	-4.8190451	0.044351	0.76004899
H	-5.0862942	-0.005529	-1.379481
H	-4.3028302	0.091436	2.83365607
C	-1.5450881	0.08836	2.63204694
C	-1.83225	0.150498	4.0042572
C	0.74886602	0.083062	3.11026812
C	-0.797175	0.1767	4.93166018
H	-2.8605139	0.180723	4.34474707
C	0.52420998	0.141663	4.48007679
H	1.75364399	0.057031	2.70384407
H	-1.018326	0.22532	5.99352503
N	-0.249625	0.054835	2.20996809
H	1.36292398	0.16159999	5.16712523
Cl	-6.5589519	0.0563	1.09380805
C	0.013642	-0.253478	-1.975484
C	0.064705	0.74396002	-2.9698701
C	-0.0115	-1.602918	-2.4371841
C	0.08983	0.448093	-4.3335872
H	0.080373	1.78885996	-2.6751211
C	0.016017	-1.91599	-3.808027
C	0.065888	-0.887573	-4.737627
H	0.127515	1.24207699	-5.0732989
H	-0.000704	-2.9429929	-4.1560121
C	-0.073275	-2.6517999	-1.4068
C	-0.130106	-4.032475	-1.651455
C	-0.134152	-3.05585	0.90060198
C	-0.187556	-4.926054	-0.588239
H	-0.132116	-4.4053111	-2.668777
C	-0.189113	-4.4320869	0.718445
H	-0.136389	-2.617249	1.89217603
H	-0.232169	-5.9944358	-0.776338
N	-0.075718	-2.188324	-0.125166
H	-0.234274	-5.0916829	1.57796001
Cl	0.101659	-1.2782511	-6.465302
C	0.22574399	2.00352693	0.012151
C	1.57519901	2.466012	0.013096
C	-0.773104	2.9978981	0.001717
C	1.88679099	3.83750892	0.00836
C	-0.478591	4.36209011	-0.005983
H	-1.817927	2.70249891	-0.005316
C	0.85705298	4.76689816	-0.001534
H	2.9136939	4.18620586	0.012432
H	-1.273589	5.10161495	-0.015189
C	2.6258831	1.43567204	0.013603
C	4.00754118	1.68012202	-0.012198
C	3.03239298	-0.871952	0.038876
C	4.90278721	0.61677498	-0.009799
H	4.37996483	2.69728208	-0.036926
C	4.40956783	-0.689909	0.017248
H	2.59450698	-1.863683	0.056652
H	5.97192717	0.80473	-0.030411
H	5.0705061	-1.54958	0.018803
N	2.16318893	0.15404101	0.039118
Cl	1.24587798	6.49535084	-0.00635

Table S1(18). Optimized parameter of T_1 state of **1**_*3*-**Cl**.

Atoms	X	Y	Z
Ir	-0.060753	-0.003237	0.031613
C	-2.0356319	0.040717	0.17910001
C	-2.9899819	0.007696	-0.852636
C	-2.5363841	0.109085	1.582165
C	-4.3635349	0.052465	-0.616477
H	-2.646049	-0.041623	-1.880994
C	-3.950824	0.165251	1.81132102
C	-4.8037019	0.135069	0.73697799
H	-5.0858321	0.03368	-1.423911
H	-4.353888	0.228902	2.81504202
C	-1.57897	0.097292	2.61455202
C	-1.8511421	0.14293399	4.02092409
C	0.74327803	0.059605	3.09579492
C	-0.826441	0.151618	4.93279696
H	-2.8808169	0.169055	4.36136818
C	0.519328	0.115344	4.46799421
H	1.75456202	0.026456	2.70167899
H	-1.037877	0.186138	5.99736309
N	-0.237074	0.036327	2.18552804
H	1.35911703	0.127618	5.15330601
Cl	-6.5410891	0.20127299	1.02565503
C	-0.011455	-0.265188	-1.989971
C	0.034834	0.72344398	-2.9894221
C	-0.011166	-1.619527	-2.431751
C	0.075372	0.41337901	-4.350214
H	0.032154	1.77131295	-2.704248
C	0.030937	-1.946658	-3.7988689
C	0.072607	-0.926691	-4.7387929
H	0.107163	1.19947696	-5.0983319
H	0.031646	-2.9769161	-4.1370759
C	-0.052369	-2.6609421	-1.390357
C	-0.075646	-4.0447002	-1.621788
C	-0.099799	-3.0469561	0.92579597
C	-0.111576	-4.9280462	-0.548898
H	-0.067863	-4.428225	-2.6348519
C	-0.123401	-4.4249048	0.75472701
H	-0.109429	-2.592469	1.91028404
H	-0.130784	-5.9987578	-0.727505
N	-0.065466	-2.1923521	-0.111041
H	-0.152724	-5.079495	1.61854303
Cl	0.125137	-1.335565	-6.4602408
C	0.209225	2.00664091	0.035218
C	1.55959797	2.4588151	0.032095
C	-0.789983	2.99834991	0.057407
C	1.87220299	3.82953811	0.054444
C	-0.491365	4.36117792	0.075647
H	-1.834365	2.70241904	0.050159
C	0.84511602	4.76184893	0.074881
H	2.89974904	4.17526484	0.05762
H	-1.285014	5.10166597	0.090001
C	2.60946107	1.42765498	0.004795
C	3.99018002	1.67342997	-0.037526
C	3.01428103	-0.879877	-0.01885
C	4.88394022	0.609321	-0.06837
H	4.36302805	2.69045997	-0.049832
C	4.39092302	-0.697507	-0.058756
H	2.57708001	-1.871836	-0.011367
H	5.95268393	0.79720199	-0.101512
H	5.05144596	-1.556991	-0.082884
N	2.14830709	0.147273	0.015664
Cl	1.238886	6.48646498	0.101243

Table S1(19). Optimized parameter of S_0 state of **1_*4*-Cl**.

Atoms	X	Y	Z
Ir	0.003898	-0.031778	0.060867
C	-2.018786	-0.035256	0.241786
C	-2.9250381	-0.10512	-0.831607
C	-2.546154	-0.007245	1.58015203
C	-4.3013482	-0.165204	-0.63669
H	-2.537638	-0.114692	-1.845153
C	-3.9488339	-0.113103	1.73949397
C	-4.8206549	-0.182721	0.65705699
H	-4.9788752	-0.214606	-1.485796
H	-5.8878279	-0.259979	0.83164197
C	-1.533681	0.13345499	2.65392804
C	-1.759583	0.355744	4.02379894
C	0.78536499	0.175304	3.06735396
C	-0.692409	0.46718901	4.90968895
H	-2.7643819	0.45294401	4.39995098
C	0.61237597	0.362183	4.43214798
H	1.77406895	0.112751	2.62757802
H	-0.885707	0.638843	5.96429777
N	-0.242356	0.070832	2.20819402
H	1.47402203	0.43771601	5.08611822
Cl	-4.7744012	-0.226058	3.31694007
C	0.068747	-0.281431	-1.953194
C	0.163118	0.76029801	-2.8933289
C	0.055074	-1.63727	-2.43488
C	0.260084	0.51875401	-4.2601261
H	0.161175	1.78625095	-2.540055
C	0.197684	-1.844093	-3.8281441
C	0.29092199	-0.791929	-4.7342181
H	0.32760301	1.344275	-4.9646912
H	0.39618599	-1.002798	-5.7924161
C	-0.112955	-2.67626	-1.390458
C	-0.32472	-4.0539298	-1.575207
C	-0.221468	-3.009845	0.93949503
C	-0.465339	-4.9028812	-0.481735
H	-0.390902	-4.4650011	-2.5687339
C	-0.399873	-4.3804288	0.80832201
H	-0.18887	-2.53563	1.91362095
H	-0.628484	-5.9641318	-0.643441
N	-0.08931	-2.186384	-0.113859
H	-0.498976	-5.0046358	1.68942499
Cl	0.32981801	-3.4484849	-4.5971141
C	0.25193599	1.98398399	0.071588
C	1.60759902	2.46580291	0.082535
C	-0.791411	2.92635012	0.11209
C	1.81148398	3.8620429	0.196523
C	-0.551733	4.29523993	0.180188
H	-1.817229	2.5732491	0.091536
C	0.75783902	4.77021694	0.236561
H	-1.378407	5.00121307	0.206221
H	0.966223	5.83071709	0.32174501
C	2.65058494	1.41827595	-0.02971
C	4.03166819	1.59889603	-0.22147
C	2.98911691	-0.913046	-0.053997
C	4.88478708	0.50308299	-0.308169
H	4.4420228	2.59063911	-0.31475
C	4.36292315	-0.785076	-0.208642
H	2.51571703	-1.886566	0.000978
H	5.9487319	0.66123801	-0.456583
H	4.99028206	-1.667714	-0.265078
N	2.16164494	0.14242201	0.02574
Cl	3.41234207	4.63223982	0.36000901

Table S1(20). Optimized parameter of T_1 state of **1_*4*-Cl**.

Atoms	X	Y	Z
Ir	-0.018655	-0.025113	0.034765
C	-2.0427389	-0.035989	0.243163
C	-2.949527	-0.126144	-0.826914
C	-2.559325	-0.003471	1.58257198
C	-4.323432	-0.206347	-0.622878
H	-2.5660219	-0.128077	-1.841315
C	-3.95871	-0.135159	1.75103605
C	-4.834271	-0.226725	0.673186
H	-5.0035119	-0.270212	-1.468508
H	-5.8988118	-0.32245	0.85415602
C	-1.546509	0.1605	2.6522131
C	-1.771712	0.42032099	4.01508522
C	0.77255303	0.209392	3.05871105
C	-0.701953	0.55130398	4.89520216
H	-2.77564	0.53239799	4.389503
C	0.60166699	0.429308	4.41880417
H	1.760692	0.133448	2.62026405
H	-0.892399	0.75158298	5.94516277
N	-0.258532	0.085845	2.20719099
H	1.46431696	0.51721197	5.06966782
Cl	-4.7660389	-0.262003	3.33226991
C	-0.006443	-0.225982	-1.938228
C	-0.054457	0.81224602	-2.883213
C	0.078929	-1.6485421	-2.413888
C	0.031932	0.57951599	-4.2519908
H	-0.155866	1.83121502	-2.5244401
C	0.305307	-1.808633	-3.8294621
C	0.25534999	-0.746881	-4.7053042
H	-0.018621	1.39021695	-4.9716158
H	0.41461599	-0.932706	-5.7631669
C	-0.062345	-2.65185	-1.4267
C	-0.228004	-4.0661011	-1.617016
C	-0.232909	-3.0153389	0.925484
C	-0.373339	-4.9148569	-0.548977
H	-0.26239	-4.4627991	-2.620398
C	-0.362498	-4.3897471	0.773184
H	-0.246131	-2.5632639	1.91249704
H	-0.509919	-5.979229	-0.715528
N	-0.088069	-2.164422	-0.097643
H	-0.470174	-5.0258098	1.64430594
Cl	0.75993198	-3.3691821	-4.5650749
C	0.27098399	1.99679601	0.055552
C	1.63617694	2.44226003	0.068862
C	-0.752738	2.95594096	0.101791
C	1.87210906	3.833812	0.182206
C	-0.4829	4.31991482	0.173134
H	-1.785739	2.62422705	0.079647
C	0.83686298	4.76365185	0.225326
H	-1.2931449	5.04418802	0.20270599
H	1.06892395	5.81931877	0.30919501
C	2.65932989	1.37085104	-0.027177
C	4.04408407	1.52167106	-0.213715
C	2.95635509	-0.971663	-0.005293
C	4.87584305	0.40733901	-0.274849
H	4.47482109	2.50301909	-0.322469
C	4.3327322	-0.870288	-0.15306
H	2.45839095	-1.931886	0.065378
H	5.94319105	0.54364198	-0.420048
H	4.9462018	-1.763477	-0.188609
N	2.15248394	0.102791	0.045473
Cl	3.48737597	4.56926584	0.33811301

Table S1(21). Optimized parameter of S_0 state of **1_5-Cl**.

Atoms	X	Y	Z
Ir	-0.01453	-0.013101	0.042668
C	-2.0304699	-0.042325	0.204973
C	-2.9431591	-0.15747	-0.861979
C	-2.5790839	0.009231	1.52876306
C	-4.3203511	-0.241921	-0.661728
H	-2.55914	-0.186564	-1.877251
C	-3.973654	-0.10315	1.71835899
C	-4.83851	-0.226819	0.63631099
H	-4.9892068	-0.329615	-1.515546
H	-4.3979578	-0.108122	2.71058297
H	-5.9078832	-0.313111	0.80853099
C	-1.598145	0.149096	2.62678695
C	-1.8016	0.36768499	4.01221991
C	0.73660398	0.128491	3.06234598
C	-0.732396	0.42767501	4.90330505
C	0.56689298	0.29214001	4.42891216
H	1.72239304	0.050138	2.61944103
H	-0.927589	0.588983	5.9576602
N	-0.294666	0.063735	2.20841193
H	1.42116702	0.332017	5.09481192
Cl	-3.380183	0.635764	4.74886179
C	0.074701	-0.243651	-1.96444
C	0.207689	0.79157799	-2.910475
C	0.041526	-1.586098	-2.4680181
C	0.326327	0.544505	-4.2775712
H	0.22328299	1.81956303	-2.561235
C	0.187738	-1.8227691	-3.8522279
C	0.32827201	-0.770571	-4.7507138
H	0.42691901	1.37520003	-4.9731712
H	0.204996	-2.8287079	-4.2423911
H	0.44051099	-0.979245	-5.8111472
C	-0.121613	-2.6505201	-1.45373
C	-0.322882	-4.044292	-1.613696
C	-0.175874	-3.001821	0.89506799
C	-0.413496	-4.896524	-0.515257
C	-0.325881	-4.3746939	0.76986301
H	-0.132809	-2.5215981	1.865345
H	-0.560344	-5.9585562	-0.677411
N	-0.081724	-2.185616	-0.163929
H	-0.390808	-5.009614	1.645895
Cl	-0.527287	-4.8414321	-3.172271
C	0.217765	1.99631202	0.079631
C	1.56164598	2.49648404	0.06113
C	-0.81707	2.9478879	0.17008799
C	1.79972601	3.88293004	0.18057901
C	-0.568512	4.31685495	0.26126701
H	-1.846292	2.60180593	0.17455
C	0.74756902	4.78703213	0.27881801
H	2.80662704	4.27016783	0.211623
H	-1.399119	5.01652384	0.32905301
H	0.95681	5.84926081	0.37127399
C	2.62699199	1.47670305	-0.050012
C	4.02313423	1.62978899	-0.238442
C	2.97909594	-0.871498	-0.002765
C	4.87734795	0.529616	-0.267937
C	4.35453892	-0.751018	-0.132702
H	2.49915004	-1.8400559	0.071308
H	5.94141817	0.686257	-0.405791
H	4.9908638	-1.628307	-0.148468
N	2.16084909	0.18951599	0.032319
Cl	4.81921577	3.17930603	-0.505986

Table S1(22). Optimized parameter of T_1 state of **1_*5*-Cl**.

Atoms	X	Y	Z
Ir	-0.031171	-0.008075	0.006947
C	-2.0451529	-0.054029	0.184248
C	-2.948282	-0.190096	-0.886945
C	-2.5918629	0.000178	1.50622201
C	-4.3240118	-0.295933	-0.688463
H	-2.560045	-0.211076	-1.9002711
C	-3.983887	-0.136973	1.69111705
C	-4.843791	-0.282942	0.60784298
H	-4.9881902	-0.398066	-1.543715
H	-4.4095178	-0.144944	2.68243289
H	-5.9115839	-0.386345	0.77875799
C	-1.618264	0.162956	2.60787606
C	-1.833737	0.39675301	3.98884797
C	0.71364403	0.18562099	3.05117702
C	-0.76785	0.48609501	4.88172817
C	0.535263	0.365991	4.41451311
H	1.70283699	0.114943	2.61510491
H	-0.96928	0.65804201	5.93319082
N	-0.314761	0.090477	2.19778609
H	1.38589001	0.42844099	5.0831461
Cl	-3.4180501	0.64693302	4.71396112
C	0.033296	-0.209461	-1.9579051
C	0.127277	0.83540702	-2.9036691
C	0.004061	-1.613441	-2.4546349
C	0.221726	0.60146099	-4.2685261
H	0.128022	1.85796297	-2.53969
C	0.16138101	-1.813236	-3.8609741
C	0.25659999	-0.742842	-4.7280541
H	0.29086301	1.422979	-4.975256
H	0.22092301	-2.811697	-4.2678919
H	0.37186599	-0.939801	-5.7915659
C	-0.136522	-2.634198	-1.4741761
C	-0.3502	-4.0544848	-1.6135941
C	-0.057288	-3.0043211	0.90156198
C	-0.349154	-4.9076309	-0.540127
C	-0.156147	-4.379993	0.76815099
H	0.022272	-2.5450871	1.88210595
H	-0.53131	-5.9660459	-0.688505
N	-0.057722	-2.1581891	-0.134214
H	-0.137218	-5.024919	1.63856602
Cl	-0.738255	-4.797431	-3.1743219
C	0.234163	2.0112071	0.05747
C	1.58608902	2.4820261	0.068985
C	-0.785676	2.97461796	0.14599299
C	1.84783995	3.8608501	0.218778
C	-0.514143	4.33806419	0.26493201
H	-1.820725	2.64639807	0.127077
C	0.80945802	4.78171921	0.31469101
H	2.86070895	4.22910976	0.27762899
H	-1.332498	5.05186892	0.329851
H	1.03640103	5.83786011	0.430594
C	2.63992405	1.44630301	-0.033333
C	4.03709078	1.58467305	-0.222495
C	2.96912909	-0.908575	0.038098
C	4.87896776	0.473912	-0.2354
C	4.34615278	-0.801058	-0.085316
H	2.47239804	-1.868497	0.116715
H	5.9447279	0.618536	-0.373757
H	4.97605896	-1.682935	-0.088658
N	2.16637897	0.163435	0.056724
Cl	4.84441996	3.12096596	-0.516899

Table S1(23). Optimized parameter of S_0 state of **1_*6*-Cl**.

Atoms	X	Y	Z
Ir	-0.015881	-0.01068	0.039131
C	-2.0365691	0.014008	0.21629301
C	-2.995157	-0.012311	-0.816904
C	-2.5467589	0.049652	1.54948401
C	-4.3668661	-0.004298	-0.558509
H	-2.6576929	-0.035313	-1.849013
C	-3.9309959	0.054538	1.80688596
C	-4.8431239	0.028213	0.75833201
H	-5.0720148	-0.023813	-1.38702
H	-4.3048601	0.078358	2.8268261
H	-5.9103298	0.031669	0.96170199
C	-1.551849	0.087407	2.62755895
C	-1.850575	0.15623	3.99854112
C	0.73824	0.091875	3.12012005
C	-0.811289	0.189803	4.91633892
H	-2.8767731	0.18665799	4.34074211
C	0.51698798	0.15737601	4.48936176
H	1.747455	0.067307	2.72503304
N	-0.252769	0.054893	2.21392298
H	1.34562802	0.184522	5.18595123
Cl	-1.170619	0.27672601	6.62924623
C	0.020266	-0.253653	-1.9743561
C	0.076833	0.746795	-2.965831
C	-0.004264	-1.603192	-2.440717
C	0.106361	0.442972	-4.327919
H	0.092459	1.78978205	-2.663167
C	0.028064	-1.906358	-3.8152599
C	0.082943	-0.88889	-4.760746
H	0.147889	1.24755704	-5.0593128
H	0.010912	-2.937984	-4.1562052
H	0.108099	-1.127866	-5.8202572
C	-0.071745	-2.647917	-1.412173
C	-0.134315	-4.0279021	-1.6679241
C	-0.141552	-3.0653269	0.89173502
C	-0.198682	-4.9112382	-0.600601
H	-0.13634	-4.402935	-2.6829269
C	-0.203021	-4.4409771	0.71342498
H	-0.145254	-2.637609	1.887923
N	-0.07516	-2.1920941	-0.126914
H	-0.254646	-5.1101141	1.56323099
Cl	-0.278243	-6.6349378	-0.905948
C	0.225586	2.00307298	0.018528
C	1.57499301	2.47055888	0.021316
C	-0.776655	2.99447608	0.012358
C	1.87622094	3.84595299	0.021835
C	-0.474636	4.35725021	0.009632
H	-1.81949	2.690943	0.004489
C	0.85706902	4.79114723	0.01544
H	2.90769911	4.18776178	0.027595
H	-1.280508	5.08837891	0.003129
H	1.09457695	5.85128784	0.016245
C	2.62191796	1.44212604	0.015686
C	4.00298119	1.69779694	-0.016819
C	3.04254007	-0.862192	0.030695
C	4.88840818	0.63028997	-0.022222
H	4.37738085	2.71273208	-0.041582
C	4.4192729	-0.68391	0.002373
H	2.615731	-1.85864	0.04671
H	5.09009314	-1.533936	-0.003349
N	2.16719794	0.156739	0.04012
Cl	6.61348391	0.93554699	-0.063575

Table S1(24). Optimized parameter of T_1 state of **1**_*6*-**Cl**.

Atoms	X	Y	Z
Ir	-0.061071	0.005092	0.035044
C	-2.0384891	0.038574	0.178509
C	-2.9847961	-0.000667	-0.865475
C	-2.5479701	0.101402	1.57048094
C	-4.3556328	0.040462	-0.628548
H	-2.6263311	-0.047533	-1.8891751
C	-3.9601181	0.150738	1.78241503
C	-4.8288322	0.119788	0.71236199
H	-5.063674	0.02018	-1.45169
H	-4.3613172	0.21053	2.78952503
H	-5.900065	0.15569501	0.897021
C	-1.58941	0.088649	2.60931492
C	-1.8742271	0.127496	4.0103879
C	0.73372197	0.073429	3.10212803
C	-0.846001	0.145583	4.91082811
H	-2.902185	0.14060099	4.35277081
C	0.51312298	0.127535	4.47177315
H	1.74910295	0.052668	2.71799707
N	-0.240818	0.036763	2.18482709
H	1.33982205	0.15215901	5.16968918
Cl	-1.175006	0.18769599	6.64220381
C	-0.000609	-0.263778	-1.987851
C	0.060081	0.72475898	-2.9864349
C	-0.004356	-1.620056	-2.428416
C	0.107054	0.40201601	-4.3444891
H	0.06221	1.77172005	-2.6966319
C	0.044629	-1.941956	-3.797997
C	0.098795	-0.935899	-4.756218
H	0.148634	1.19579506	-5.0871191
H	0.041167	-2.977602	-4.1260152
H	0.13548601	-1.1899509	-5.8117352
C	-0.055338	-2.6541729	-1.385309
C	-0.087937	-4.0380578	-1.623607
C	-0.114386	-3.0465789	0.929169
C	-0.13483	-4.9084468	-0.544142
H	-0.080045	-4.4267831	-2.6332159
C	-0.148528	-4.4240279	0.76582301
H	-0.12451	-2.598887	1.91672003
N	-0.06891	-2.1898029	-0.103798
H	-0.186844	-5.0857558	1.62200499
Cl	-0.178436	-6.6345282	-0.828188
C	0.20992599	2.01367903	0.048268
C	1.56110203	2.46838689	0.040252
C	-0.792678	3.00190592	0.084607
C	1.86429501	3.8422389	0.070377
C	-0.485076	4.36292124	0.109142
H	-1.8351311	2.69848204	0.081326
C	0.84788001	4.79000187	0.103261
H	2.89629006	4.18077993	0.070538
H	-1.288406	5.09575605	0.13278501
H	1.08985102	5.84864807	0.124896
C	2.60616899	1.43790495	0.001853
C	3.98596096	1.693856	-0.051868
C	3.02201009	-0.866509	-0.038137
C	4.86905479	0.62499601	-0.096771
H	4.36159182	2.70839405	-0.063096
C	4.39777517	-0.688855	-0.090976
H	2.59464407	-1.862599	-0.032211
H	5.0669179	-1.539354	-0.127075
N	2.15177798	0.15463001	0.011781
Cl	6.59154177	0.92821002	-0.165207

Table S1(25). Optimized parameter of S_0 state of **1_7-Cl**.

Atoms	X	Y	Z
Ir	-0.013559	-0.012152	0.041181
C	-2.034641	0.012784	0.217888
C	-2.992419	-0.013772	-0.815355
C	-2.543216	0.04872	1.55139506
C	-4.364409	-0.005566	-0.556644
H	-2.655057	-0.03704	-1.847469
C	-3.9274211	0.053898	1.80878603
C	-4.8398991	0.027453	0.76019502
H	-5.0697041	-0.025242	-1.38501
H	-4.3001828	0.078251	2.82909489
H	-5.9070659	0.031204	0.96397299
C	-1.550436	0.086577	2.63087893
C	-1.837691	0.155047	4.00490618
C	0.748456	0.091	3.11282706
C	-0.812988	0.189731	4.94011116
H	-2.86535	0.184476	4.34627199
C	0.50390399	0.156188	4.47875118
H	1.75853705	0.067186	2.7215209
H	-1.0285291	0.24344499	6.00167799
N	-0.251098	0.05462	2.21785307
Cl	1.85083306	0.19895799	5.59781218
C	0.021411	-0.253919	-1.972829
C	0.077264	0.74698597	-2.9631481
C	-0.003006	-1.603567	-2.4380491
C	0.106288	0.44334799	-4.3256311
H	0.092717	1.789868	-2.6602011
C	0.028789	-1.906204	-3.8126869
C	0.082967	-0.888343	-4.7581749
H	0.14726201	1.24807405	-5.0568838
H	0.011528	-2.93805	-4.152875
H	0.107635	-1.127328	-5.8177352
C	-0.070161	-2.650162	-1.411951
C	-0.132825	-4.0327292	-1.656628
C	-0.140571	-3.0573821	0.90026802
C	-0.19828	-4.9343009	-0.603644
H	-0.133753	-4.4066162	-2.6732409
C	-0.201604	-4.430388	0.69806403
H	-0.145098	-2.633744	1.89759195
H	-0.24769	-6.0023208	-0.785959
N	-0.074017	-2.195354	-0.126275
Cl	-0.285719	-5.5049939	2.07882905
C	0.226281	2.00215006	0.019758
C	1.57568395	2.46880698	0.021437
C	-0.776636	2.9921391	0.014136
C	1.87607503	3.84434891	0.021141
C	-0.475112	4.35536385	0.010734
H	-1.819297	2.68806911	0.007197
C	0.85632402	4.78928709	0.015183
H	2.9076941	4.18565798	0.025706
H	-1.281284	5.08614683	0.004671
H	1.09361303	5.84951591	0.015295
C	2.62469912	1.44306898	0.015561
C	4.00827312	1.68801796	-0.017088
C	3.03575301	-0.869483	0.03088
C	4.91222382	0.63507497	-0.023128
H	4.38122511	2.70467997	-0.041374
C	4.40976906	-0.666948	0.002196
H	2.61334395	-1.8672071	0.046604
H	5.98103523	0.817608	-0.048998
N	2.17133498	0.157142	0.040005
Cl	5.48760605	-2.0477171	-0.005826

Table S1(26). Optimized parameter of T_1 state of **1_*7*-Cl**.

Atoms	X	Y	Z
Ir	-0.049144	0.005723	0.037185
C	-2.0299339	0.035414	0.184834
C	-2.9775779	-0.002103	-0.857044
C	-2.538805	0.090622	1.57560599
C	-4.3478122	0.033582	-0.616774
H	-2.621846	-0.043672	-1.8819081
C	-3.953002	0.133935	1.79099298
C	-4.8227358	0.104845	0.72438401
H	-5.0562549	0.014945	-1.439853
H	-4.3503938	0.18782599	2.79985809
H	-5.8940058	0.13604601	0.909006
C	-1.5835331	0.079273	2.61455989
C	-1.858155	0.1172	4.01638603
C	0.74886501	0.076892	3.09686208
C	-0.85155	0.141119	4.94427109
H	-2.8897901	0.125291	4.35230398
C	0.49647501	0.130142	4.45917511
H	1.76757705	0.06291	2.72350097
H	-1.0550031	0.16808701	6.00844288
N	-0.229777	0.032728	2.18425798
Cl	1.83639801	0.178073	5.58950901
C	0.007112	-0.26412	-1.985656
C	0.072736	0.72393	-2.9843869
C	-0.004714	-1.620659	-2.425679
C	0.117066	0.40105101	-4.3427072
H	0.081114	1.77089906	-2.694726
C	0.04157	-1.942355	-3.79546
C	0.100915	-0.936707	-4.7540908
H	0.16277701	1.19466305	-5.0853248
H	0.031979	-2.9782519	-4.1225872
H	0.135571	-1.191239	-5.8096252
C	-0.061225	-2.6570139	-1.3856061
C	-0.103138	-4.0433512	-1.614363
C	-0.121691	-3.040915	0.93591797
C	-0.155122	-4.9316549	-0.549603
H	-0.097562	-4.4303398	-2.6258259
C	-0.16418	-4.416132	0.748496
H	-0.129203	-2.597477	1.92498302
H	-0.188848	-6.0022788	-0.720074
N	-0.071058	-2.1948929	-0.103618
Cl	-0.232678	-5.4786949	2.13742709
C	0.213385	2.01401806	0.044819
C	1.56426203	2.47095203	0.038252
C	-0.790953	3.00039697	0.076724
C	1.86467803	3.845469	0.066579
C	-0.486007	4.36224222	0.099735
H	-1.832958	2.69521093	0.071342
C	0.84622902	4.79149103	0.096025
H	2.89636111	4.18509579	0.067528
H	-1.290617	5.09382105	0.120001
H	1.08625996	5.85067415	0.116151
C	2.6127789	1.44475698	0.002212
C	3.99520111	1.69143605	-0.049488
C	3.02179694	-0.86744	-0.037037
C	4.89706182	0.63784498	-0.093945
H	4.36864424	2.70797706	-0.05917
C	4.3952322	-0.664803	-0.088161
H	2.59926009	-1.865086	-0.032055
H	5.96533298	0.820539	-0.134864
N	2.16138792	0.16019601	0.011467
Cl	5.47105694	-2.043767	-0.145584

Table S1(27). Optimized parameter of S_0 state of **1_*2*-OMe**.

Atoms	X	Y	Z
Ir	-0.002083	-0.036444	0.054788
C	-2.0197539	0.019758	0.26060501
C	-2.988318	0.00343	-0.751058
C	-2.5003991	0.069268	1.60945296
C	-4.3638701	0.034041	-0.473959
H	-2.691334	-0.028297	-1.794995
C	-3.877574	0.096209	1.87838602
C	-4.8194299	0.080208	0.85315198
H	-4.2407599	0.13084599	2.90211105
H	-5.8762479	0.101418	1.09073901
C	-1.490051	0.095742	2.6694479
C	-1.752947	0.16494	4.04965305
C	0.81314301	0.073935	3.11767101
C	-0.704936	0.186259	4.96015406
H	-2.7764051	0.203979	4.40415907
C	0.61151201	0.13981301	4.48991489
H	1.81128895	0.038527	2.694947
H	-0.909936	0.240058	6.0253458
N	-0.198331	0.04921	2.23118091
H	1.46040404	0.15621001	5.16461802
O	-5.1879072	0.015639	-1.5643719
C	-6.5952692	0.049462	-1.3511601
H	-6.900887	0.96558398	-0.830841
H	-7.0495982	0.028967	-2.343245
H	-6.936038	-0.82161	-0.777906
C	-0.000015	-0.305847	-1.955969
C	0.048769	0.67442101	-2.9551649
C	-0.044088	-1.6690511	-2.395005
C	0.051096	0.35450599	-4.3217378
H	0.078055	1.72738194	-2.6912551
C	-0.038045	-1.980916	-3.7633481
C	0.007931	-0.986021	-4.7363172
H	-0.070115	-3.01527	-4.0955401
H	0.011226	-1.256901	-5.7853351
C	-0.100894	-2.696871	-1.353025
C	-0.170039	-4.0842609	-1.575065
C	-0.138058	-3.0736749	0.96276098
C	-0.221321	-4.9618292	-0.500321
H	-0.185745	-4.469553	-2.5878949
C	-0.205008	-4.4512429	0.80181402
H	-0.125867	-2.6203451	1.94806004
H	-0.275058	-6.032639	-0.673605
N	-0.084466	-2.219146	-0.074671
H	-0.245213	-5.0993772	1.67038
O	0.096484	1.41838896	-5.1787
C	0.0923	1.16168904	-6.5791922
H	-0.819886	0.63676	-6.8886938
H	0.128342	2.13909006	-7.0634208
H	0.96737701	0.573865	-6.8828359
C	0.26622301	1.97334003	0.002021
C	1.62977004	2.41335893	-0.013344
C	-0.716169	2.971766	-0.011085
C	1.93993998	3.78194404	-0.037108
C	-0.397721	4.33835316	-0.038088
H	-1.7691441	2.70643306	-0.007318
C	0.94315201	4.7539382	-0.050664
H	2.97447109	4.11507893	-0.046301
H	1.21275306	5.80313206	-0.068431
C	2.65980291	1.37199605	-0.010238
C	4.0483551	1.59411299	-0.049195
C	3.03942204	-0.943243	0.033515
C	4.92786503	0.51975	-0.044346
H	4.43309784	2.60658598	-0.085968
C	4.41814899	-0.782128	-0.001667
H	2.58684111	-1.928493	0.064244
H	5.99956989	0.69307703	-0.075269
H	5.0678792	-1.650429	0.001807
N	2.18291712	0.093917	0.032177
O	-1.463238	5.19440794	-0.05083
C	-1.207641	6.59473515	-0.083234
H	-0.654204	6.88141298	-0.985994
H	-2.186074	7.07816219	-0.090775
H	-0.648181	6.92185307	0.80192

Table S1(28). Optimized parameter of T_1 state of **1_*2*-OMe**.

Atoms	X	Y	Z
Ir	-0.008681	-0.018149	0.050994
C	-2.008394	0.040159	0.230211
C	-2.9705169	0.019955	-0.766329
C	-2.4820631	0.089836	1.63643599
C	-4.355484	0.059051	-0.48702
H	-2.676501	-0.016852	-1.810799
C	-3.902756	0.139356	1.89228702
C	-4.810494	0.1232	0.85832202
H	-4.2720938	0.19342799	2.91108203
H	-5.8713708	0.16029701	1.08023202
C	-1.509961	0.07213	2.64860511
C	-1.760402	0.096541	4.06404209
C	0.82495201	0.067154	3.09317803
C	-0.72909	0.113153	4.9631381
H	-2.7869461	0.097676	4.41555119
C	0.61647803	0.108077	4.47119999
H	1.834077	0.055664	2.69113398
H	-0.922963	0.12968799	6.03120184
N	-0.158921	0.033817	2.19152498
H	1.46663499	0.131971	5.14363289
O	-5.1823921	0.038152	-1.570014
C	-6.5926518	0.084069	-1.365709
H	-6.8932462	1.00912905	-0.85953
H	-7.0389471	0.054679	-2.3607791
H	-6.9407582	-0.778702	-0.785327
C	-0.001463	-0.310558	-1.966926
C	0.05848	0.65823799	-2.974551
C	-0.050924	-1.678466	-2.3870859
C	0.065672	0.32289401	-4.3380461
H	0.092648	1.71411395	-2.722151
C	-0.041219	-2.0058351	-3.7519009
C	0.015045	-1.022148	-4.7357669
H	-0.078112	-3.0433221	-4.0732732
H	0.020604	-1.305696	-5.7814469
C	-0.107689	-2.697283	-1.334383
C	-0.177907	-4.0866652	-1.544785
C	-0.1336	-3.056432	0.98692298
C	-0.225941	-4.954483	-0.462071
H	-0.196561	-4.4811249	-2.5538969
C	-0.203339	-4.4347711	0.83668298
H	-0.114486	-2.5905969	1.96641505
H	-0.281102	-6.0265861	-0.62663
N	-0.08585	-2.213074	-0.059196
H	-0.240207	-5.0771222	1.70960999
O	0.122241	1.375808	-5.206316
C	0.12467	1.10339606	-6.6041861
H	-0.789348	0.58142102	-6.9129729
H	0.170405	2.07512212	-7.098753
H	0.99757499	0.50630099	-6.895411
C	0.25988299	1.98780298	0.003593
C	1.622684	2.42814589	-0.012662
C	-0.727644	2.98002005	0.001299
C	1.92691398	3.7978189	-0.022446
C	-0.413454	4.34787512	-0.012582
H	-1.778927	2.7088809	-0.000189
C	0.92640197	4.76617813	-0.023603
H	2.96000504	4.13467121	-0.02958
H	1.19295096	5.81621313	-0.03238
C	2.656111	1.39003801	-0.022284
C	4.04332209	1.61731505	-0.070903
C	3.04191899	-0.924531	-0.006999
C	4.92518997	0.54500097	-0.085707
H	4.42509079	2.63107395	-0.100253
C	4.4196949	-0.758791	-0.053802
H	2.59206605	-1.911105	0.018142
H	5.99607992	0.72152102	-0.123845
H	5.07200098	-1.624983	-0.06595
N	2.18442798	0.110919	0.012554
O	-1.4809001	5.199646	-0.014645
C	-1.230265	6.601686	-0.026394
H	-0.679733	6.90346193	-0.925869
H	-2.210566	7.0811348	-0.024875
H	-0.670481	6.91676998	0.86276698

Table S1(29). Optimized parameter of S_0 state of **1_*3*-OMe**.

Atoms	X	Y	Z
Ir	0.003436	0.015876	-0.000694
C	-2.023087	0.07474	0.142992
C	-2.9737401	0.049865	-0.892042
C	-2.5532839	0.13719501	1.46697605
C	-4.3545308	0.083956	-0.66305
H	-2.6314571	0.005978	-1.922102
C	-3.935281	0.169642	1.70933604
C	-4.8419709	0.14360601	0.64919102
H	-4.340416	0.215657	2.71573806
C	-1.5745879	0.17192701	2.56388092
C	-1.885177	0.24933399	3.93125606
C	0.71003801	0.15647499	3.08576393
C	-0.86798	0.278202	4.87768698
H	-2.920192	0.28818399	4.25016308
C	0.461721	0.231058	4.45078278
H	1.72198403	0.119686	2.69774604
H	-1.108315	0.33805901	5.9350009
N	-0.270855	0.124329	2.16632795
H	1.28798199	0.25310701	5.15292311
H	-5.0348191	0.062886	-1.508139
O	-6.171792	0.178903	0.99382901
C	-7.1320162	0.15679	-0.053653
H	-7.059371	-0.761686	-0.650472
H	-8.108901	0.19311699	0.432302
H	-7.0266171	1.02419901	-0.718164
C	0.067625	-0.271286	-2.0114451
C	0.147664	0.69332302	-3.0308271
C	0.033179	-1.630102	-2.4482789
C	0.18924899	0.36659601	-4.3915639
H	0.172971	1.74580204	-2.76244
C	0.07601	-1.970104	-3.809294
C	0.15378401	-0.977239	-4.7864661
H	0.049723	-3.0026701	-4.1447129
C	-0.05773	-2.655056	-1.397424
C	-0.120907	-4.0410161	-1.615378
C	-0.17078	-3.015156	0.91555297
C	-0.208482	-4.913764	-0.537413
H	-0.103407	-4.4308901	-2.6262579
C	-0.23431	-4.3943381	0.75969601
H	-0.190616	-2.5569661	1.89837205
H	-0.257578	-5.9854331	-0.7058
N	-0.08213	-2.1669869	-0.124171
H	-0.303897	-5.0367551	1.63069797
H	0.24898	1.16114497	-5.128294
O	0.190308	-1.415253	-6.088521
C	0.26275	-0.437145	-7.1170421
H	0.27843499	-0.989991	-8.0582428
H	-0.609384	0.229366	-7.1038208
H	1.17576802	0.167087	-7.0376539
C	0.27831301	2.02784896	-0.063401
C	1.63459396	2.47349501	-0.040796
C	-0.69395	3.04155898	-0.11978
C	1.96486795	3.837291	-0.070786
C	-0.376812	4.40479994	-0.151965
H	-1.744401	2.76540208	-0.145065
C	0.96465898	4.80842781	-0.126305
H	2.99538994	4.17956686	-0.053217
C	2.66805911	1.42828405	0.007513
C	4.05430412	1.65330994	0.017497
C	3.04434896	-0.883622	0.083374
C	4.93535185	0.57952601	0.062774
H	4.4381299	2.66619802	-0.0118
C	4.42408991	-0.720625	0.096891
H	2.59261608	-1.86935	0.106282
H	6.00724888	0.75337201	0.070019
H	5.0732789	-1.588719	0.131044
N	2.18791103	0.15231501	0.04237
H	-1.1767761	5.13672686	-0.196683
O	1.39360905	6.11376286	-0.151312
C	0.40828601	7.13635206	-0.207963
H	-0.252147	7.11117792	0.668513
H	0.95482802	8.08129311	-0.219147
H	-0.201625	7.06089783	-1.117542

Table S1(30). Optimized parameter of T_1 state of **1_*3*-OMe**.

Atoms	X	Y	Z
Ir	0.0029	0.017494	-0.055191
C	-2.0232389	0.061535	0.116024
C	-2.9716599	0.008864	-0.917991
C	-2.5442381	0.130484	1.44061196
C	-4.3516059	0.02397	-0.68504
H	-2.629456	-0.039615	-1.9476481
C	-3.92577	0.14373501	1.68445206
C	-4.8355899	0.091457	0.62772298
H	-4.328227	0.1927	2.69135809
C	-1.565501	0.186859	2.53657007
C	-1.878673	0.27649701	3.90245199
C	0.71822101	0.20667	3.05830002
C	-0.861759	0.32927099	4.84784603
H	-2.913867	0.30593899	4.22095203
C	0.46848401	0.29461899	4.42210817
H	1.73111796	0.177149	2.67306995
H	-1.103021	0.39803201	5.90431404
N	-0.262983	0.149958	2.14149189
H	1.29408705	0.335033	5.12398481
H	-5.032661	-0.017849	-1.52837
O	-6.1625662	0.110725	0.97611803
C	-7.1272068	0.048441	-0.066811
H	-7.0355249	-0.878747	-0.646854
H	-8.1020412	0.071307	0.42369199
H	-7.0440502	0.90643501	-0.746234
C	0.046515	-0.24486	-2.0103221
C	0.121432	0.73876202	-3.0201931
C	-0.00937	-1.6679651	-2.4449329
C	0.13691001	0.44181499	-4.3806248
H	0.157285	1.78183901	-2.7219839
C	0.007469	-1.9571331	-3.8292079
C	0.077229	-0.932204	-4.7726188
H	-0.031667	-2.975584	-4.2020969
C	-0.064662	-2.6510069	-1.42409
C	-0.121104	-4.0650821	-1.6202411
C	-0.139215	-3.008584	0.91925299
C	-0.187246	-4.9223361	-0.549131
H	-0.112641	-4.4588509	-2.6318419
C	-0.201588	-4.3872762	0.77267402
H	-0.146919	-2.555125	1.90673602
H	-0.230015	-5.996459	-0.707133
N	-0.061528	-2.151788	-0.11031
H	-0.258388	-5.0262232	1.64691103
H	0.189069	1.23204601	-5.1188312
O	0.084527	-1.330749	-6.0636711
C	0.15335	-0.355228	-7.1086969
H	0.143801	-0.923554	-8.039238
H	-0.711168	0.315828	-7.0796471
H	1.07761896	0.227974	-7.0442491
C	0.295508	2.03966689	-0.109233
C	1.65515304	2.46645188	-0.0456
C	-0.665161	3.05945611	-0.187533
C	2.00065804	3.82624507	-0.064422
C	-0.333208	4.41989422	-0.206388
H	-1.7171691	2.79271889	-0.243291
C	1.01144803	4.80800104	-0.144728
H	3.03336	4.15856123	-0.018588
C	2.67807698	1.41104698	0.043754
C	4.06496716	1.62300801	0.10721
C	3.03084612	-0.909182	0.15432701
C	4.93148899	0.53985202	0.19362301
H	4.46059895	2.63137889	0.088235
C	4.40980291	-0.756957	0.21832
H	2.5606041	-1.886532	0.169723
H	6.00380182	0.70417798	0.24132399
H	5.0513072	-1.628763	0.28339401
N	2.19225502	0.137759	0.06901
H	-1.123515	5.16056395	-0.270165
O	1.454898	6.10690689	-0.156034
C	0.48307499	7.14208889	-0.231431
H	-0.19797	7.12015676	0.62898499
H	1.04149997	8.07994175	-0.224648
H	-0.10502	7.07788277	-1.155923

Table S1(31). Optimized parameter of S_0 state of **1_*4*-OMe**.

Atoms	X	Y	Z
Ir	-0.032692	-0.006144	0.032225
C	-2.0544241	0.015785	0.22117899
C	-2.9806931	-0.021892	-0.839397
C	-2.569634	0.056619	1.55635798
C	-4.352715	-0.0166	-0.608962
H	-2.6155961	-0.051166	-1.86104
C	-3.9784739	0.056653	1.77289295
C	-4.8621168	0.022155	0.691459
H	-5.9329991	0.02244	0.85122901
C	-1.563709	0.104244	2.63264394
C	-1.800894	0.173958	4.02076912
C	0.75258899	0.124831	3.0601089
C	-0.738109	0.217172	4.91600084
H	-2.816618	0.194791	4.38138103
C	0.57169902	0.192055	4.43497276
H	1.74442601	0.106248	2.62233996
H	-0.934629	0.27135801	5.98302412
N	-0.269244	0.079175	2.1883769
H	1.42988098	0.22552601	5.09736776
H	-5.0477209	-0.043656	-1.4461221
O	-4.427206	0.090993	3.06573296
C	-5.8289838	0.093462	3.31655312
H	-6.3068671	-0.813945	2.9288671
H	-5.9342799	0.123404	4.40221596
H	-6.3127861	0.97473001	2.87908196
C	0.010112	-0.268333	-1.980679
C	0.076203	0.75708902	-2.9440379
C	-0.018701	-1.621694	-2.447295
C	0.108071	0.47646701	-4.3063588
H	0.095989	1.79151404	-2.6163161
C	0.017761	-1.889752	-3.8467989
C	0.079499	-0.841679	-4.7684121
H	0.107411	-1.040764	-5.8323159
C	-0.095339	-2.6607959	-1.404436
C	-0.156196	-4.0568442	-1.592721
C	-0.184182	-3.0033851	0.92452502
C	-0.22979	-4.9126778	-0.499518
H	-0.146534	-4.4539828	-2.5948751
C	-0.244375	-4.3840518	0.792059
H	-0.195678	-2.52911	1.89960003
H	-0.276679	-5.9863091	-0.658283
N	-0.109115	-2.16977	-0.126826
H	-0.30256	-5.0148258	1.67245197
H	0.15607899	1.28751695	-5.0305648
O	-0.010137	-3.1979959	-4.2489162
C	0.015913	-3.4991579	-5.6405392
H	-0.017443	-4.5877519	-5.7074828
H	-0.852626	-3.0752361	-6.1582179
H	0.93518299	-3.132896	-6.1126061
C	0.227997	2.00736809	0.014753
C	1.58136904	2.47485399	0.014384
C	-0.799442	2.97102809	0.016854
C	1.84746206	3.87523103	0.018869
C	-0.520607	4.33404398	0.016171
H	-1.83384	2.64257097	0.013331
C	0.797598	4.79682779	0.017738
H	0.99523401	5.86136293	0.020625
C	2.62283111	1.43172097	0.00062
C	4.01982594	1.61996305	-0.033004
C	2.96907806	-0.898303	0.002207
C	4.8779521	0.526196	-0.046783
H	4.41599703	2.62240791	-0.049766
C	4.35072803	-0.765899	-0.027975
H	2.49607706	-1.873995	0.013227
H	5.95229006	0.684865	-0.0735
H	4.98337412	-1.6468101	-0.03902
N	2.13309002	0.15370899	0.018733
H	-1.333078	5.0582509	0.015143
O	3.15576506	4.27806616	0.024855
C	3.45541906	5.67024422	0.02518
H	3.05693007	6.16551399	0.918477
H	4.54449177	5.73761606	0.028343
H	3.06215191	6.16479492	-0.870822

Table S1(32). Optimized parameter of T_1 state of **1_*4*-OMe**.

Atoms	X	Y	Z
Ir	-0.05191	-0.018291	0.019893
C	-2.0416601	-0.023143	0.18608899
C	-2.971442	-0.126036	-0.860666
C	-2.544919	0.078832	1.58097994
C	-4.34235	-0.083194	-0.634353
H	-2.6042581	-0.217515	-1.8780299
C	-3.9836381	0.169634	1.76658297
C	-4.8386579	0.078576	0.690359
H	-5.9104638	0.133442	0.84211302
C	-1.578463	0.057639	2.61216092
C	-1.792454	0.068911	4.03327608
C	0.77648002	0.05133	3.0322659
C	-0.741955	0.081843	4.91287708
H	-2.8094029	0.061622	4.39592218
C	0.59605402	0.088766	4.40586901
H	1.77538705	0.044592	2.60630488
H	-0.923182	0.085225	5.98361111
N	-0.230047	0.011385	2.14570808
H	1.45687604	0.115644	5.06486607
H	-5.0483451	-0.149981	-1.457439
O	-4.4223199	0.351428	3.05424309
C	-5.8202581	0.45933601	3.28873992
H	-6.3490682	-0.45493	2.99113607
H	-5.9322929	0.60979301	4.36378193
H	-6.2508392	1.31478906	2.75321388
C	0.01108	-0.297538	-2.0027499
C	0.086957	0.72391897	-2.965584
C	-0.010106	-1.653595	-2.454771
C	0.126692	0.43505001	-4.3268428
H	0.104834	1.76000798	-2.6424179
C	0.031209	-1.9299671	-3.8526959
C	0.097337	-0.885521	-4.7790041
H	0.128337	-1.091166	-5.8415308
C	-0.068913	-2.689462	-1.405333
C	-0.109812	-4.0868769	-1.588095
C	-0.131442	-3.0260029	0.92939198
C	-0.161795	-4.93817	-0.490082
H	-0.101336	-4.4887719	-2.588258
C	-0.172834	-4.4072161	0.80100399
H	-0.139885	-2.541837	1.89976895
H	-0.193992	-6.0128522	-0.644881
N	-0.080048	-2.199574	-0.127964
H	-0.214251	-5.0374508	1.68255198
H	0.178978	1.24111795	-5.0558848
O	0.002769	-3.2385621	-4.2476659
C	0.031525	-3.5484259	-5.6381698
H	-0.0038	-4.637229	-5.6978169
H	-0.835321	-3.125972	-6.1594892
H	0.95273697	-3.1867311	-6.1095638
C	0.195586	1.99188197	0.044936
C	1.54240704	2.47253895	0.040008
C	-0.850451	2.93313289	0.08375
C	1.78604996	3.87589192	0.078977
C	-0.59059	4.29958391	0.113103
H	-1.8780921	2.58529997	0.077366
C	0.72045898	4.7797122	0.112231
H	0.90259898	5.84657717	0.138955
C	2.5995841	1.44606304	-0.008739
C	3.99218893	1.65838897	-0.057309
C	2.97999001	-0.877348	-0.081808
C	4.86544991	0.57827401	-0.11778
H	4.37315607	2.66672206	-0.049548
C	4.35855484	-0.721907	-0.131397
H	2.52249098	-1.8602721	-0.090379
H	5.93664503	0.75407898	-0.155705
H	5.00440407	-1.591749	-0.179307
N	2.13150597	0.161713	-0.01769
H	-1.413254	5.01103592	0.137835
O	3.085814	4.29767323	0.083351
C	3.36660004	5.69444084	0.117435
H	2.96962905	6.15968084	1.02710903
H	4.45452881	5.77562284	0.11286
H	2.95871806	6.20549583	-0.762337

Table S1(33). Optimized parameter of S_0 state of **1_5-OMe**.

Atoms	X	Y	Z
Ir	-0.037193	0.000031	0.037726
C	-2.055676	0.00837	0.21275499
C	-2.9882021	-0.030271	-0.84258
C	-2.587049	0.043004	1.54391897
C	-4.3667202	-0.03283	-0.624042
H	-2.6202481	-0.054912	-1.864357
C	-3.983367	0.03744	1.75503695
C	-4.8677359	0.000637	0.68042499
H	-4.3781929	0.062781	2.76002598
H	-5.9394498	-0.003319	0.86272699
C	-1.588196	0.087337	2.63106608
C	-1.8332731	0.148022	4.03574896
C	0.73615497	0.107369	3.06691504
C	-0.759885	0.184368	4.92725992
C	0.54528898	0.163656	4.43664885
H	1.72572505	0.092957	2.62653303
H	-0.932672	0.229867	5.99517012
N	-0.295666	0.068219	2.20453095
H	1.39507794	0.192836	5.10997009
H	-5.0505438	-0.061293	-1.470557
O	-3.126771	0.168851	4.44808912
C	-3.409313	0.236983	5.84581518
H	-4.49686	0.246507	5.92295694
H	-3.0123651	-0.637037	6.37455511
H	-3.0026541	1.15297103	6.28928423
C	0.023108	-0.250341	-1.9716981
C	0.09202	0.76821399	-2.942456
C	0.003417	-1.6010081	-2.452656
C	0.135758	0.497228	-4.3110042
H	0.10592	1.80346704	-2.613827
C	0.049907	-1.865199	-3.8390639
C	0.114984	-0.825327	-4.7625008
H	0.033829	-2.884409	-4.1961889
H	0.150122	-1.0486391	-5.8258519
C	-0.072728	-2.6498351	-1.415234
C	-0.123457	-4.0630021	-1.608394
C	-0.170216	-2.996738	0.922337
C	-0.19529	-4.9128842	-0.503364
C	-0.21915	-4.3728118	0.78215998
H	-0.189375	-2.517786	1.89372098
H	-0.233911	-5.9867778	-0.636379
N	-0.097143	-2.1747069	-0.139912
H	-0.276411	-5.0133629	1.655514
H	0.18592501	1.31706595	-5.0253782
O	-0.099273	-4.524508	-2.8851049
C	-0.156092	-5.9320941	-3.116348
H	-0.127242	-6.0505648	-4.1998348
H	0.70393503	-6.4433889	-2.669112
H	-1.085425	-6.3618221	-2.72558
C	0.21315099	2.01037788	0.035663
C	1.56418204	2.49088693	0.034727
C	-0.806216	2.98284292	0.047918
C	1.82788098	3.87814999	0.047239
C	-0.535636	4.3520999	0.057178
H	-1.841774	2.65480208	0.045732
C	0.787296	4.8029542	0.057679
H	2.84738803	4.23481083	0.04841
H	1.01029801	5.86690998	0.067651
C	2.61404896	1.45202005	0.019847
C	4.02785921	1.64390004	-0.015328
C	2.96255898	-0.887162	0.03272
C	4.87894392	0.537534	-0.019079
C	4.33937216	-0.748165	0.006575
H	2.48439598	-1.859036	0.046642
H	5.95338392	0.66956198	-0.043537
H	4.98090696	-1.622672	0.00304
N	2.13922596	0.17657501	0.040526
H	-1.356058	5.06753397	0.064471
O	4.488585	2.9207871	-0.046059
C	5.89660406	3.15043998	-0.09845
H	6.33725786	2.71247911	-1.001277
H	6.01409197	4.23411179	-0.124463
H	6.39830399	2.74910998	0.789388

Table S1(34). Optimized parameter of T_1 state of **1_*5*-OMe**.

Atoms	X	Y	Z
Ir	-0.065983	0.028056	0.024799
C	-2.0454929	0.051342	0.180134
C	-2.974545	0.005406	-0.878143
C	-2.5738969	0.108565	1.57074702
C	-4.3496351	0.045921	-0.672685
H	-2.5939469	-0.044075	-1.893898
C	-4.0011611	0.167606	1.74307406
C	-4.8460379	0.135759	0.657754
H	-4.4152789	0.23581	2.7380991
H	-5.9204569	0.18097199	0.82386702
C	-1.617679	0.089736	2.61045003
C	-1.8438	0.100663	4.04798985
C	0.73736399	0.150106	3.05078912
C	-0.788213	0.161918	4.93349791
C	0.54136503	0.205621	4.42266512
H	1.73854494	0.16191401	2.63160801
H	-0.954356	0.17017201	6.0027442
N	-0.263614	0.068497	2.16719699
H	1.39185297	0.26754501	5.09236097
H	-5.0400262	0.022463	-1.5114141
O	-3.1393199	0.037039	4.44534779
C	-3.433161	0.043173	5.84170198
H	-4.519918	-0.009772	5.91416979
H	-2.9866741	-0.823795	6.34165287
H	-3.075398	0.96400601	6.31595898
C	0.005466	-0.254644	-1.991647
C	0.085321	0.74835402	-2.9742811
C	-0.01157	-1.613277	-2.4448681
C	0.136289	0.454631	-4.338479
H	0.099293	1.78885698	-2.6618519
C	0.041097	-1.900416	-3.8264041
C	0.112849	-0.87522	-4.7659831
H	0.02506	-2.9247971	-4.167963
H	0.15139399	-1.116771	-5.8251138
C	-0.079106	-2.648526	-1.390735
C	-0.119238	-4.0647039	-1.5642819
C	-0.158835	-2.9653909	0.95623797
C	-0.177653	-4.8983579	-0.445577
C	-0.197903	-4.34341	0.83368099
H	-0.175232	-2.4659591	1.91754305
H	-0.208503	-5.9742751	-0.563941
N	-0.101025	-2.1622701	-0.119961
H	-0.245545	-4.9742889	1.71448302
H	0.192371	1.26162004	-5.066525
O	-0.098802	-4.5440021	-2.833137
C	-0.142816	-5.9557209	-3.0451801
H	-0.118234	-6.0879092	-4.127079
H	0.72467101	-6.4518061	-2.5955391
H	-1.0660681	-6.3885441	-2.64393
C	0.21206699	2.03210092	0.039442
C	1.56566799	2.49998808	0.037409
C	-0.805191	3.00516391	0.06985
C	1.83445501	3.88550401	0.068225
C	-0.526816	4.37219	0.095674
H	-1.841359	2.6799891	0.062292
C	0.79841697	4.81477499	0.09621
H	2.85559201	4.23669481	0.069839
H	1.02722895	5.87708712	0.11808
C	2.61136103	1.45757496	0.003907
C	4.02525806	1.64622104	-0.038688
C	2.94923711	-0.882432	-0.032439
C	4.87066889	0.53576899	-0.073874
C	4.32638407	-0.747789	-0.070786
H	2.46816492	-1.852753	-0.030863
H	5.94539118	0.66374999	-0.105402
H	4.96427679	-1.624367	-0.098777
N	2.13328791	0.185002	0.006787
H	-1.343498	5.09104109	0.114754
O	4.4900732	2.92057991	-0.044822
C	5.89921713	3.14713097	-0.099757
H	6.33260822	2.72937012	-1.015398
H	6.01948977	4.23069	-0.100595
H	6.40367794	2.7230649	0.77567601

Table S1(35). Optimized parameter of S_0 state of **1**_*6*-**OMe**.

Atoms	X	Y	Z
Ir	-0.046757	0.010472	0.014676
C	-2.0626991	0.050511	0.233585
C	-3.044672	0.02585	-0.779082
C	-2.546382	0.090555	1.57709706
C	-4.4114389	0.039252	-0.494445
H	-2.7278791	-0.0009	-1.817999
C	-3.9245031	0.100933	1.86082196
C	-4.8600202	0.075899	0.83158302
H	-4.2758808	0.127978	2.88905406
H	-5.9229102	0.083477	1.05778897
C	-1.53004	0.12526099	2.64078808
C	-1.798218	0.19549	4.00832176
C	0.762254	0.122124	3.0874629
C	-0.749833	0.228728	4.93514204
H	-2.814786	0.229985	4.38098383
C	0.57056201	0.190139	4.46150684
H	1.76552796	0.094358	2.67604899
N	-0.237678	0.087172	2.19471693
H	1.42869401	0.214332	5.1202631
H	-5.1332851	0.020459	-1.308875
O	-1.103417	0.29839301	6.23664093
C	-0.069805	0.34685799	7.2248292
H	-0.583417	0.406138	8.18461704
H	0.548204	-0.557257	7.19459009
H	0.56209898	1.23168397	7.09125185
C	-0.043829	-0.256204	-1.995793
C	0.004724	0.73252797	-3.0010331
C	-0.07845	-1.61083	-2.4480081
C	0.017626	0.41551501	-4.3606558
H	0.028131	1.77888596	-2.7092471
C	-0.062943	-1.9271621	-3.8189621
C	-0.015338	-0.920698	-4.7781129
H	-0.087273	-2.963304	-4.1466332
H	-0.002985	-1.1723059	-5.8352361
C	-0.135983	-2.6502349	-1.407733
C	-0.2071	-4.0233302	-1.64564
C	-0.176715	-3.0436561	0.89392501
C	-0.263794	-4.9254718	-0.576944
H	-0.224925	-4.4188838	-2.653894
C	-0.247108	-4.4215031	0.73271501
H	-0.16589	-2.6093099	1.88789296
N	-0.119879	-2.17451	-0.125552
H	-0.289749	-5.0601902	1.60520005
H	0.053844	1.212479	-5.1010861
O	-0.333333	-6.2344799	-0.90164
C	-0.408852	-7.1978769	0.153506
H	-0.464521	-8.1690359	-0.338689
H	0.48303699	-7.1589022	0.78853798
H	-1.305055	-7.0437622	0.76434201
C	0.21671	2.0206399	-0.024648
C	1.57086098	2.47536707	-0.039039
C	-0.774985	3.02422309	-0.024172
C	1.88393104	3.84715891	-0.047769
C	-0.461118	4.3845458	-0.036006
H	-1.820915	2.73009205	-0.018719
C	0.874677	4.80452919	-0.046728
H	2.9196949	4.17687321	-0.055074
H	1.12378001	5.86230087	-0.05289
C	2.61328506	1.43664002	-0.05104
C	3.98706007	1.67609096	-0.100317
C	3.01186705	-0.864388	-0.03099
C	4.89222383	0.60853899	-0.114188
H	4.3809948	2.68457198	-0.133924
C	4.39055014	-0.70158	-0.077395
H	2.57935095	-1.858896	-0.005913
H	5.03154182	-1.573338	-0.087115
N	2.13978601	0.154026	-0.015038
H	-1.260188	5.1235919	-0.036436
O	6.20168114	0.93484598	-0.164875
C	7.1688571	-0.119078	-0.19501
H	8.13983822	0.37441799	-0.241606
H	7.11236286	-0.73302	0.71061498
H	7.03550577	-0.75133	-1.079627

Table S1(36). Optimized parameter of T_1 state of **1_*6*-OMe**.

Atoms	X	Y	Z
Ir	-0.050472	0.078605	0.004091
C	-2.0765569	0.098882	0.232806
C	-3.059267	0.09092	-0.774646
C	-2.54333	0.103381	1.58015394
C	-4.4245782	0.09101	-0.479072
H	-2.7485371	0.090716	-1.8158211
C	-3.918983	0.102382	1.87477803
C	-4.8609052	0.097222	0.85077602
H	-4.2638278	0.106584	2.90526104
H	-5.921864	0.09701	1.08533704
C	-1.517648	0.106114	2.63728809
C	-1.774011	0.121038	4.00769186
C	0.78450298	0.088607	3.06669402
C	-0.716332	0.120924	4.92606783
H	-2.7868919	0.13474201	4.39091921
C	0.602301	0.103957	4.44180822
H	1.78169703	0.07549	2.64008594
N	-0.226557	0.08885	2.18516803
H	1.464275	0.104655	5.09569883
H	-5.152956	0.087463	-1.287409
O	-1.058791	0.136655	6.22894716
C	-0.019447	0.14015999	7.21435785
H	-0.529613	0.15483101	8.17745781
H	0.59735698	-0.761716	7.13855791
H	0.610551	1.030797	7.1181159
C	-0.024623	-0.240144	-2.002064
C	0.044944	0.73896599	-3.0128219
C	-0.061481	-1.599432	-2.428576
C	0.07369	0.40357301	-4.3676872
H	0.066976	1.78745604	-2.7307379
C	-0.029444	-1.93106	-3.795331
C	0.036781	-0.937246	-4.766181
H	-0.054686	-2.9706409	-4.109973
H	0.060556	-1.203368	-5.819304
C	-0.134344	-2.627274	-1.3772171
C	-0.210965	-4.001163	-1.603884
C	-0.206959	-2.9964681	0.92720002
C	-0.28681	-4.892096	-0.526446
H	-0.218194	-4.406702	-2.608027
C	-0.284356	-4.3747401	0.77847302
H	-0.204522	-2.553515	1.91713703
N	-0.130049	-2.1401839	-0.101351
H	-0.341366	-5.0044522	1.656551
H	0.12521499	1.190925	-5.1167369
O	-0.359785	-6.2019029	-0.838633
C	-0.45318	-7.1564221	0.22427
H	-0.507537	-8.1309938	-0.260977
H	0.431292	-7.1157379	0.86914098
H	-1.356258	-6.992126	0.821859
C	0.168465	2.04368091	-0.075301
C	1.59413195	2.48918104	-0.12923
C	-0.826173	3.03760791	-0.076155
C	1.86928201	3.89749503	-0.199274
C	-0.527613	4.40063906	-0.137719
H	-1.866684	2.72802305	-0.040027
C	0.83892697	4.8096261	-0.201698
H	2.89471006	4.25145912	-0.248407
H	1.07180595	5.87151718	-0.253234
C	2.59226394	1.49513102	-0.088684
C	4.00731611	1.72298503	-0.122155
C	2.99567389	-0.827051	0.001299
C	4.89053106	0.66068101	-0.100937
H	4.40339994	2.73075795	-0.16574
C	4.38384724	-0.665426	-0.041423
H	2.57260203	-1.826305	0.050094
H	5.02305984	-1.5374	-0.026227
N	2.12154889	0.174325	-0.009611
H	-1.3172931	5.14612103	-0.143297
O	6.21791315	0.96493101	-0.138277
C	7.16288424	-0.103361	-0.129275
H	8.14519978	0.369791	-0.168836
H	7.08476686	-0.70078	0.78670597
H	7.03914213	-0.755687	-1.0016609

Table S1(37). Optimized parameter of S_0 state of **1_*7*-OMe**.

Atoms	X	Y	Z
Ir	-0.041263	0.017095	0.011599
C	-2.0669811	-0.007585	0.156168
C	-3.0095229	-0.037238	-0.890902
C	-2.59459	-0.005024	1.48273003
C	-4.3863931	-0.05986	-0.654412
H	-2.6550591	-0.038097	-1.918043
C	-3.9810729	-0.029891	1.71805501
C	-4.8798142	-0.056195	0.65521699
H	-4.3683782	-0.028254	2.73375511
H	-5.9497862	-0.075348	0.844854
C	-1.6147749	0.030118	2.57938409
C	-1.911517	0.060978	3.94797802
C	0.67367297	0.068705	3.0910449
C	-0.899378	0.095365	4.90239477
H	-2.943527	0.060923	4.27935791
C	0.43200999	0.099192	4.46954393
H	1.69272602	0.073986	2.72093296
H	-1.152584	0.120108	5.95576477
N	-0.308852	0.034771	2.18427706
H	-5.0790091	-0.080326	-1.49376
O	1.53072	0.132047	5.26598978
C	1.33247006	0.18254399	6.67918015
H	0.79775602	-0.705062	7.03704214
H	2.330549	0.20745499	7.11760902
H	0.78211802	1.08497095	6.970294
C	0.030242	-0.18899	-2.00755
C	0.075808	0.827389	-2.982549
C	0.04442	-1.531721	-2.492919
C	0.12909099	0.54758298	-4.3504171
H	0.063822	1.86541605	-2.6617219
C	0.099879	-1.810698	-3.870357
C	0.14147	-0.776959	-4.801836
H	0.110413	-2.8380771	-4.2255449
H	0.18441699	-1.000561	-5.8645339
C	-0.007694	-2.597326	-1.480098
C	-0.027159	-3.9746771	-1.734664
C	-0.093285	-3.0375791	0.82180899
C	-0.078959	-4.897284	-0.694148
H	-0.004561	-4.337636	-2.755666
C	-0.112523	-4.4230032	0.622711
H	-0.122527	-2.6361871	1.82858002
H	-0.094281	-5.9581499	-0.914735
N	-0.042208	-2.1616709	-0.187655
H	0.160861	1.36473203	-5.0686498
O	-0.164905	-5.1848149	1.74502301
C	-0.202051	-6.6038561	1.59070504
H	0.69926399	-6.9721141	1.08687103
H	-0.24583	-7.010644	2.60145497
H	-1.090416	-6.9186769	1.03063798
C	0.151364	2.03901696	0.029153
C	1.49036503	2.53377295	0.061297
C	-0.871874	3.00795603	0.021893
C	1.75944102	3.91416407	0.082171
C	-0.601759	4.37867403	0.040878
H	-1.90745	2.68002796	-0.004394
C	0.71931899	4.83933401	0.070692
H	2.78405905	4.27648687	0.106384
H	0.93527502	5.90435886	0.08676
C	2.56311297	1.52725995	0.066044
C	3.9389801	1.790398	0.076912
C	3.0193119	-0.773129	0.060435
C	4.86885118	0.75507498	0.081898
H	4.29533005	2.81400108	0.079823
C	4.4035182	-0.565362	0.074462
H	2.62492108	-1.7830271	0.049993
H	5.92838001	0.98241401	0.089713
N	2.13633704	0.231436	0.057401
H	-1.423802	5.09196186	0.031943
O	5.17307615	-1.683607	0.077773
C	6.59162617	-1.520837	0.083186
H	6.92656422	-0.988615	0.98121101
H	7.00553322	-2.5296431	0.082417
H	6.93267012	-0.984648	-0.810144

Table S1(38). Optimized parameter of T_1 state of **1_*7*-OMe**.

Atoms	X	Y	Z
Ir	-0.052681	0.019989	-0.005839
C	-2.073451	-0.023894	0.154194
C	-3.01407	-0.070637	-0.893154
C	-2.5940599	-0.024965	1.48246706
C	-4.3893361	-0.11535	-0.65365
H	-2.6586239	-0.062203	-1.919803
C	-3.979604	-0.072981	1.71852398
C	-4.8791032	-0.117284	0.65718502
H	-4.3651929	-0.075848	2.73456812
H	-5.948091	-0.152839	0.84912997
C	-1.614075	0.028468	2.57814789
C	-1.911052	0.06573	3.94652009
C	0.67377502	0.11095	3.08657098
C	-0.898455	0.126396	4.89879894
H	-2.9424241	0.050568	4.2791338
C	0.43263	0.150879	4.46508503
H	1.69236398	0.128472	2.71574211
H	-1.151003	0.155497	5.95217705
N	-0.309624	0.050359	2.18329191
H	-5.0828261	-0.147641	-1.491515
O	1.53041399	0.210977	5.25961924
C	1.33336401	0.26781401	6.67316723
H	0.81604803	-0.627224	7.03773117
H	2.33158207	0.31451601	7.10932779
H	0.76674497	1.16190195	6.95844793
C	0.008042	-0.160495	-1.993412
C	0.0557	0.855151	-2.9618051
C	0.012613	-1.562183	-2.48049
C	0.086488	0.58659297	-4.3322492
H	0.054959	1.88966501	-2.6304851
C	0.035063	-1.808808	-3.900456
C	0.072257	-0.763213	-4.7885408
H	0.022373	-2.8278511	-4.2757449
H	0.09075	-0.966485	-5.856977
C	0.006873	-2.577306	-1.514003
C	0.012875	-3.9883561	-1.764321
C	-0.08147	-3.0361381	0.82139099
C	-0.032764	-4.9052248	-0.751994
H	0.056146	-4.3371458	-2.790813
C	-0.09191	-4.4130659	0.60057801
H	-0.122316	-2.6661401	1.84141004
H	-0.025359	-5.9668841	-0.965057
N	-0.016911	-2.1272161	-0.157221
H	0.114218	1.39893198	-5.0535522
O	-0.15432	-5.1963759	1.71065903
C	-0.19826	-6.613771	1.54737794
H	0.70779598	-6.9868422	1.05636406
H	-0.259376	-7.0253911	2.55555201
H	-1.080157	-6.9213371	0.973813
C	0.159086	2.05047607	0.023542
C	1.50309801	2.52824306	0.05853
C	-0.854903	3.02668405	0.026383
C	1.78546	3.90594697	0.086431
C	-0.571821	4.39499807	0.053636
H	-1.893566	2.70802307	-0.000906
C	0.75390297	4.84090996	0.082159
H	2.81304312	4.25931215	0.110804
H	0.980883	5.9035058	0.102945
C	2.56964707	1.51267505	0.068436
C	3.94741988	1.76659703	0.088932
C	3.01177812	-0.793824	0.076257
C	4.86895609	0.72404802	0.104086
H	4.31188583	2.78718805	0.09238
C	4.39699507	-0.594632	0.099001
H	2.60332704	-1.798344	0.068399
H	5.92983723	0.94472802	0.119096
N	2.1396451	0.218799	0.060603
H	-1.386373	5.116642	0.050833
O	5.16056919	-1.7158411	0.112106
C	6.58035517	-1.5609371	0.124844
H	6.9129529	-1.0274071	1.02288306
H	6.98820782	-2.5721171	0.130072
H	6.92901087	-1.030094	-0.768641

Table S1(39). Optimized parameter of S_0 state of **1**_*2*-**SO_2Me**.

Atoms	X	Y	Z
Ir	-0.054597	-0.046692	-0.008922
C	-2.0684919	-0.125417	0.195878
C	-3.029269	-0.266483	-0.823268
C	-2.5669351	-0.024426	1.52941501
C	-4.3928971	-0.302794	-0.526997
H	-2.71328	-0.352605	-1.856845
C	-3.9438691	-0.072822	1.80846298
C	-4.8721671	-0.210904	0.78309602
H	-4.3069739	-0.00368	2.82913899
H	-5.9352112	-0.256936	0.99034399
C	-1.560302	0.138576	2.59235907
C	-1.838348	0.26149899	3.96124911
C	0.73316199	0.31532601	3.03636789
C	-0.797901	0.41247499	4.87137222
H	-2.8628621	0.238721	4.31273985
C	0.51737499	0.44074199	4.40356398
H	1.73354495	0.330962	2.6191411
H	-1.010758	0.50717098	5.93167305
N	-0.270319	0.16616499	2.15408897
H	1.35968399	0.55769098	5.07631397
S	-5.5725079	-0.444021	-1.872077
O	-4.9259749	-1.156354	-2.995291
O	-6.8410349	-0.977962	-1.329554
C	-5.8652091	1.25785995	-2.3929241
H	-6.2929978	1.81558502	-1.557774
H	-4.919836	1.70103598	-2.7114921
H	-6.5699182	1.21689403	-3.2274029
C	-0.020765	-0.452649	-1.993957
C	-0.0569	0.46945	-3.0569789
C	0.048219	-1.832728	-2.3514271
C	-0.016337	0.039643	-4.3849249
H	-0.135809	1.53068304	-2.8481319
C	0.084741	-2.2457111	-3.6944079
C	0.055732	-1.313856	-4.7257118
H	0.13149001	-3.299499	-3.950978
H	0.075241	-1.623745	-5.764216
C	0.067812	-2.7962971	-1.237264
C	0.1172	-4.191164	-1.371286
C	0.042539	-3.0150981	1.09505498
C	0.130173	-4.9983978	-0.239052
H	0.14399099	-4.6422882	-2.3558111
C	0.092357	-4.4021001	1.02295804
H	0.010274	-2.5005441	2.04871392
H	0.168005	-6.078589	-0.340292
N	0.032427	-2.2319479	0.002516
H	0.099387	-4.9919591	1.93276596
S	-0.046341	1.26100099	-5.6996479
O	-0.430206	0.58562797	-6.9583321
O	-0.839823	2.42412591	-5.247335
C	1.66836905	1.79712796	-5.860538
H	1.99591303	2.23175097	-4.9143481
H	2.28610206	0.93838602	-6.1296811
H	1.69084597	2.54833508	-6.6541739
C	0.051332	1.96674395	-0.195678
C	1.363608	2.5260911	-0.240059
C	-1.0204411	2.87536097	-0.284717
C	1.57163	3.91107893	-0.363129
C	-0.793598	4.24720621	-0.40523
H	-2.0412631	2.51030993	-0.277737
C	0.49538499	4.78701687	-0.445906
H	2.57605791	4.3209219	-0.400892
H	0.64736301	5.85511112	-0.551524
C	2.483109	1.57159996	-0.164019
C	3.84293795	1.91116905	-0.206704
C	3.04895496	-0.694072	0.023524
C	4.81102896	0.91585302	-0.130355
H	4.14271688	2.94806409	-0.300288
C	4.41030788	-0.416432	-0.013182
H	2.68310809	-1.7107281	0.111452
H	5.86435509	1.17656505	-0.163371
H	5.12996912	-1.225263	0.047052
N	2.11045599	0.26598099	-0.046075
S	-2.2011831	5.35682678	-0.49536
O	-1.760463	6.62511492	-1.115751
O	-3.3396051	4.62907505	-1.097047
C	-2.617234	5.70037413	1.226089
H	-2.873512	4.76377821	1.72483897
H	-1.762924	6.1779151	1.70943701
H	-3.476821	6.37539721	1.21466696

Table S1.(40) Optimized parameter of T_1 state of **1**_*2*-**SO_2Me**.

Atoms	X	Y	Z
Ir	-0.075575	-0.004912	-0.008429
C	-2.097611	-0.123143	0.20276099
C	-3.0562661	-0.243214	-0.815997
C	-2.5801821	-0.076943	1.54221797
C	-4.4176149	-0.308002	-0.51204
H	-2.744046	-0.286693	-1.8534941
C	-3.9541719	-0.154374	1.82919705
C	-4.8861818	-0.268811	0.80395401
H	-4.312201	-0.1247	2.85328889
H	-5.947114	-0.335119	1.01676702
C	-1.566017	0.057175	2.60380912
C	-1.833014	0.120712	3.97838712
C	0.73516297	0.244794	3.03847098
C	-0.785163	0.24958999	4.88377094
H	-2.8532879	0.06977	4.33838797
C	0.52725399	0.3143	4.40999413
H	1.72912502	0.28845	2.6080761
H	-0.990633	0.29908699	5.94850302
N	-0.277615	0.119517	2.1633811
H	1.37160802	0.41708899	5.08213186
S	-5.6051469	-0.416478	-1.8545901
O	-4.9494119	-1.066797	-3.0091319
O	-6.856472	-1.000291	-1.3250231
C	-5.9360719	1.29992294	-2.2987931
H	-6.3760672	1.80982101	-1.4398381
H	-5.0015192	1.77893198	-2.59694
H	-6.6403251	1.279742	-3.1344321
C	0.001343	-0.441983	-1.991392
C	-0.001236	0.481951	-3.0507071
C	0.076928	-1.8214459	-2.3358121
C	0.074575	0.049619	-4.375989
H	-0.085516	1.54279196	-2.8442359
C	0.14835601	-2.2345851	-3.6773379
C	0.149551	-1.304428	-4.7100129
H	0.20032001	-3.2884259	-3.9305611
H	0.19474	-1.617445	-5.746778
C	0.0702	-2.7857251	-1.222056
C	0.113664	-4.1800728	-1.359528
C	-0.009932	-3.0088351	1.10774302
C	0.095458	-4.9891491	-0.228761
H	0.15958001	-4.6295562	-2.3437979
C	0.032013	-4.3958602	1.03325403
H	-0.059442	-2.4986789	2.06255889
H	0.128739	-6.0692158	-0.331628
N	0.011999	-2.225338	0.016373
H	0.014598	-4.9875102	1.941571
S	0.083074	1.27054703	-5.69309
O	-0.291889	0.59592199	-6.9540682
O	-0.700949	2.44321108	-5.2511339
C	1.80795598	1.77948296	-5.826622
H	2.12679291	2.21078992	-4.8759332
H	2.41651702	0.91100597	-6.0851922
H	1.85388005	2.52906299	-6.62078
C	-0.019206	1.95601702	-0.217048
C	1.35921705	2.51779294	-0.327991
C	-1.101545	2.8462019	-0.273761
C	1.51168001	3.93181896	-0.524854
C	-0.910424	4.2217679	-0.455787
H	-2.1127269	2.4636519	-0.201534
C	0.42060101	4.75489998	-0.587227
H	2.50039411	4.36499023	-0.63677
H	0.550071	5.81982279	-0.753083
C	2.43692994	1.61402202	-0.219741
C	3.8242681	1.95325899	-0.282352
C	3.03113794	-0.660475	0.05302
C	4.78619003	0.975384	-0.179046
H	4.11369896	2.99009395	-0.410634
C	4.39222622	-0.373752	-0.010031
H	2.68393111	-1.680549	0.183746
H	5.83903313	1.23687196	-0.226982
H	5.11843395	-1.174258	0.070784
N	2.07878494	0.27487901	-0.034958
S	-2.291574	5.31048203	-0.485956
O	-1.954608	6.48358107	-1.3278101
O	-3.5105829	4.53110409	-0.806707
C	-2.4926541	5.92483377	1.20278597
H	-2.6940229	5.08103514	1.86556005
H	-1.580808	6.44434118	1.50434697
H	-3.339185	6.6168561	1.19776797

Table S1(41). Optimized parameter of S_0 state of **1**_*3*-**SO_2Me**.

Atoms	X	Y	Z
Ir	0.186197	-0.208208	0.076542
C	-1.8123471	-0.18639	0.38283899
C	-2.835134	-0.270419	-0.586662
C	-2.229655	-0.0944	1.74681795
C	-4.1856408	-0.265863	-0.249506
H	-2.5682609	-0.343973	-1.636212
C	-3.58636	-0.089654	2.09721303
C	-4.5534892	-0.17419	1.09900105
H	-4.948545	-0.345972	-1.018037
H	-3.9073081	-0.035332	3.13180208
C	-1.164881	-0.006057	2.76003408
C	-1.366416	0.108429	4.14313412
C	1.15465498	0.038332	3.087816
C	-0.274263	0.18626	4.99983311
H	-2.3715439	0.13765	4.54637718
C	1.01576996	0.15090699	4.46555614
H	2.13189292	0.008373	2.61933899
H	-0.427506	0.274528	6.07093191
N	0.100151	-0.0409	2.25703907
H	1.89599097	0.210122	5.09590912
S	-6.2798281	-0.156595	1.55051696
O	-7.0350761	-0.932724	0.54306197
O	-6.3951788	-0.536337	2.97567511
C	-6.784049	1.56871605	1.40118206
H	-6.191977	2.17249298	2.09140897
H	-6.6338172	1.89625502	0.37081799
H	-7.8436408	1.61349797	1.66543198
C	0.094375	-0.557833	-1.911755
C	0.080176	0.394811	-2.953593
C	0.058717	-1.932707	-2.3004561
C	0.035437	0.031387	-4.2963548
H	0.106681	1.45166898	-2.7075839
C	0.012169	-2.3094461	-3.649447
C	-0.000069	-1.327167	-4.6363239
H	0.041523	0.78802902	-5.0751162
H	-0.000835	-3.351552	-3.949954
C	0.075691	-2.9276471	-1.2151051
C	0.023444	-4.3185668	-1.385764
C	0.16749699	-3.209801	1.10918403
C	0.046594	-5.155581	-0.275952
H	-0.037163	-4.743	-2.380676
C	0.120785	-4.5940762	1.00081003
H	0.223104	-2.7203751	2.07500696
H	0.005911	-6.2326779	-0.40529
N	0.14783899	-2.3980269	0.037396
H	0.139989	-5.2086759	1.89385998
S	-0.069121	-1.811278	-6.3526378
O	0.63508701	-0.788592	-7.1564169
O	0.36208799	-3.222424	-6.4590368
C	-1.817629	-1.738882	-6.789331
H	-2.369508	-2.4413781	-6.1622438
H	-2.180109	-0.719641	-6.6436892
H	-1.8933491	-2.0241511	-7.8416882
C	0.40574801	1.79690194	-0.059635
C	1.75231004	2.26903391	-0.140878
C	-0.609638	2.77765703	-0.071651
C	2.04407692	3.63786507	-0.208515
C	-0.331091	4.13944006	-0.146087
H	-1.648507	2.46658897	-0.02849
C	1.00288904	4.56228399	-0.208766
H	3.06418395	4.00037813	-0.27229
H	-1.135113	4.86895084	-0.166587
C	2.81137204	1.24637902	-0.155319
C	4.18639994	1.50046802	-0.261361
C	3.23741889	-1.05553	-0.063738
C	5.08958006	0.44358	-0.265199
H	4.54787588	2.51857209	-0.342896
C	4.60973501	-0.864132	-0.16402
H	2.81007004	-2.048574	0.014543
H	6.15448618	0.63772601	-0.347689
H	5.27736187	-1.718475	-0.164777
N	2.36140704	-0.035227	-0.05681
S	1.38222003	6.3044672	-0.27388
O	2.75634909	6.46540594	-0.797567
O	0.27173099	7.00074291	-0.959195
C	1.38643098	6.84478807	1.447227
H	0.400866	6.66430902	1.88021195
H	2.15636611	6.29352903	1.99020505
H	1.61201406	7.91421509	1.44351697

Table S1(42). Optimized parameter of T_1 state of **1_*3*-SO$_2$Me**.

Atoms	X	Y	Z
Ir	0.16484299	-0.201761	0.076562
C	-1.80291	-0.176912	0.36752
C	-2.8303399	-0.271824	-0.58109
C	-2.1925459	-0.065391	1.80180502
C	-4.1838388	-0.252409	-0.237128
H	-2.5693841	-0.361292	-1.631034
C	-3.5946059	-0.030225	2.14042997
C	-4.5269642	-0.120466	1.14221299
H	-4.95859	-0.348826	-0.989113
H	-3.9227779	0.063929	3.16882491
C	-1.168324	-0.013721	2.75558209
C	-1.339471	0.086383	4.17998791
C	1.18461597	0.026847	3.06147408
C	-0.252566	0.16131	5.01004314
H	-2.342108	0.100015	4.59228277
C	1.05578995	0.13886601	4.44622898
H	2.16679502	0.001355	2.59935308
H	-0.383895	0.236718	6.08492279
N	0.145402	-0.061078	2.22927594
H	1.94264197	0.203003	5.06586695
S	-6.264595	-0.068926	1.59358203
O	-6.9859271	-1.051029	0.75514197
O	-6.3807359	-0.169534	3.06380391
C	-6.8025489	1.58036399	1.102718
H	-6.2524309	2.32017207	1.68713903
H	-6.6214361	1.71411705	0.034706
H	-7.872457	1.63899195	1.31817496
C	0.071411	-0.565092	-1.920838
C	0.056349	0.38117999	-2.9649799
C	0.042369	-1.942204	-2.2942369
C	0.010655	0.007377	-4.305717
H	0.080673	1.43985903	-2.7257431
C	-0.006381	-2.329128	-3.640717
C	-0.024221	-1.353326	-4.6336112
H	0.013995	0.757631	-5.0904698
H	-0.016907	-3.3730719	-3.934448
C	0.078759	-2.931448	-1.202235
C	0.040369	-4.3235469	-1.3654521
C	0.20299301	-3.202771	1.12573099
C	0.084676	-5.153863	-0.251089
H	-0.025831	-4.754518	-2.3570261
C	0.168345	-4.5873852	1.02323902
H	0.26659501	-2.703928	2.08628392
H	0.053802	-6.231854	-0.375253
N	0.15992001	-2.399576	0.048697
H	0.203942	-5.1990242	1.91768503
S	-0.09587	-1.850809	-6.3482938
O	0.60289598	-0.83053	-7.1591749
O	0.34114	-3.2605901	-6.4436369
C	-1.846006	-1.787697	-6.7788758
H	-2.393317	-2.4875031	-6.1448498
H	-2.2114291	-0.768604	-6.6398859
H	-1.923745	-2.0812421	-7.8287959
C	0.39484999	1.80412698	-0.03362
C	1.73951805	2.27389407	-0.124645
C	-0.624564	2.77818394	-0.009769
C	2.02621007	3.64497399	-0.16726
C	-0.348224	4.14173603	-0.058394
H	-1.660882	2.45987105	0.034801
C	0.98371297	4.56641388	-0.131152
H	3.04445505	4.01063204	-0.238595
H	-1.153469	4.86981487	-0.051275
C	2.80133891	1.25495601	-0.174146
C	4.17288923	1.51646197	-0.301743
C	3.23410106	-1.0470901	-0.142767
C	5.07765388	0.46188399	-0.347831
H	4.53048182	2.53692508	-0.367513
C	4.60343504	-0.849238	-0.267787
H	2.81140995	-2.042882	-0.07656
H	6.14018297	0.66075802	-0.447003
H	5.27309084	-1.701195	-0.301669
N	2.35824609	-0.028698	-0.092887
S	1.36045206	6.31275892	-0.167268
O	2.72270489	6.48418283	-0.716663
O	0.23424201	7.0190382	-0.8146
C	1.39937401	6.81293392	1.56514502
H	0.42353499	6.61942387	2.01423907
H	2.18242788	6.25248623	2.07914305
H	1.62162006	7.88293886	1.58029199

Table S1(43). Optimized parameter of S_0 state of **1_*4*-SO_2Me**.

Atoms	X	Y	Z
Ir	-0.102955	0.054543	-0.022508
C	-2.103688	0.19960199	0.28178099
C	-3.094316	0.18233	-0.72028
C	-2.532795	0.252195	1.64596796
C	-4.4505839	0.175947	-0.419064
H	-2.787637	0.171321	-1.761348
C	-3.919395	0.105037	1.93567002
C	-4.866962	0.098796	0.91143399
H	-5.1940579	0.18334299	-1.21161
H	-5.923842	0.010281	1.12518501
C	-1.4497271	0.488383	2.62888002
C	-1.602038	0.96485001	3.94004512
C	0.88268101	0.48615199	2.91546988
C	-0.486317	1.15452898	4.74963522
H	-2.5813069	1.218683	4.32004881
C	0.78417802	0.88613302	4.24205208
H	1.84425402	0.31557301	2.44527698
H	-0.611742	1.51975703	5.76438999
N	-0.192507	0.30806699	2.12969208
H	1.680004	1.01372695	4.83956814
S	-4.5533319	-0.255065	3.59827709
O	-3.6821661	-1.282359	4.20934391
O	-4.7846861	0.99969298	4.35503912
C	-6.1710839	-1.0277391	3.37513804
H	-6.9036088	-0.30369	3.018677
H	-6.0914211	-1.886677	2.70807695
H	-6.4379129	-1.357749	4.38231421
C	-0.178831	-0.308794	-2.017195
C	-0.110861	0.66190702	-3.0365319
C	-0.235526	-1.6844389	-2.407141
C	-0.059867	0.31939301	-4.3820572
H	-0.096483	1.71187401	-2.7618101
C	-0.042215	-2.0183711	-3.777848
C	0.013158	-1.024069	-4.7553148
H	-0.029203	1.08905399	-5.1486211
H	0.136832	-1.2714961	-5.8013392
C	-0.525197	-2.6312971	-1.305348
C	-1.022315	-3.936955	-1.437413
C	-0.603196	-2.851913	1.03254402
C	-1.2625279	-4.7114658	-0.306627
H	-1.252422	-4.3390541	-2.4137881
C	-1.024282	-4.1735511	0.95745599
H	-0.455031	-2.358093	1.98605704
H	-1.6431	-5.722455	-0.41577
N	-0.376563	-2.099951	-0.057457
H	-1.191087	-4.7436099	1.86467505
S	0.31494099	-3.704612	-4.3470039
O	1.28612804	-4.3107581	-3.410526
O	-0.942541	-4.4430699	-4.6188269
C	1.17234302	-3.5404999	-5.9286928
H	0.494311	-3.190835	-6.7070298
H	2.03829908	-2.8862951	-5.8220158
H	1.49656701	-4.5601392	-6.1516552
C	0.27811101	2.04177904	-0.152526
C	1.65674102	2.42394495	-0.182444
C	-0.69075	3.06515694	-0.15228
C	1.98975599	3.801512	-0.038651
C	-0.34518	4.41042709	-0.144365
H	-1.741251	2.79245305	-0.158217
C	0.99739099	4.7823391	-0.050668
H	-1.1123101	5.1798439	-0.165137
H	1.24478996	5.83195782	0.035514
C	2.60613298	1.30549598	-0.391266
C	3.93192196	1.40738904	-0.839667
C	2.81145	-1.035647	-0.360236
C	4.70622396	0.262263	-1.000234
H	4.35119486	2.37005901	-1.0949661
C	4.14962292	-0.987133	-0.72997
H	2.30395603	-1.978327	-0.190461
H	5.73242283	0.348952	-1.344138
H	4.71887493	-1.904137	-0.834166
N	2.05973291	0.067799	-0.211873
S	3.66761398	4.38489723	0.33555001
O	4.23515987	3.50988293	1.38433695
O	4.44818401	4.57090616	-0.912138
C	3.49030709	6.02155018	1.07984102
H	3.16591692	6.75346422	0.34023499
H	2.81221509	5.97811222	1.93264997
H	4.50241613	6.26005411	1.41637897

Table S1(44). Optimized parameter of T_1 state of **1_*4*-SO₂Me**.

Atoms	X	Y	Z
Ir	-0.208644	0.025528	-0.06406
C	-2.205302	0.22069199	0.30376801
C	-3.218554	0.20038401	-0.673333
C	-2.595912	0.30764499	1.675174
C	-4.5664568	0.22632401	-0.337715
H	-2.9389091	0.16767099	-1.720722
C	-3.977145	0.197429	2.00206089
C	-4.950438	0.188125	1.003039
H	-5.3278208	0.231388	-1.112746
H	-6.00312	0.128667	1.24547195
C	-1.486302	0.52546799	2.63138103
C	-1.590848	1.04557002	3.9299829
C	0.84721398	0.41079101	2.87371898
C	-0.450261	1.19975805	4.71221685
H	-2.5492289	1.35738003	4.32039309
C	0.79442298	0.84973598	4.19042778
H	1.78918195	0.177315	2.39149094
H	-0.536206	1.59920394	5.7180562
N	-0.252417	0.270477	2.11521411
H	1.70659602	0.94426101	4.76883078
S	-4.573422	-0.10458	3.69089699
O	-3.706552	-1.132818	4.30516481
O	-4.7570038	1.17622197	4.41530609
C	-6.2113528	-0.848512	3.5292809
H	-6.9383259	-0.118291	3.17409706
H	-6.1660399	-1.72492	2.88203502
H	-6.4576702	-1.148301	4.55106115
C	-0.388932	-0.284709	-2.0082369
C	-0.606714	0.64692003	-3.028383
C	-0.299578	-1.72925	-2.3858149
C	-0.656091	0.267353	-4.3777332
H	-0.722029	1.69456196	-2.7689591
C	-0.035213	-2.0247209	-3.792511
C	-0.308844	-1.050979	-4.7449331
H	-0.864244	0.99611998	-5.1545272
H	-0.2141	-1.277017	-5.8029318
C	-0.538521	-2.661129	-1.35405
C	-0.844754	-4.0492258	-1.511725
C	-0.709667	-2.9165549	0.99364901
C	-1.062837	-4.8430238	-0.408329
H	-0.942224	-4.4610381	-2.5086479
C	-0.975652	-4.2808971	0.88489598
H	-0.662511	-2.4330781	1.96434605
H	-1.31376	-5.8920898	-0.53382
N	-0.504773	-2.1250329	-0.062075
H	-1.128034	-4.87537	1.77834105
S	0.79735303	-3.51368	-4.3077011
O	1.74316502	-3.908915	-3.2371609
O	-0.164798	-4.545671	-4.774178
C	1.79405606	-3.0597391	-5.7472262
H	1.15607703	-2.8146999	-6.596755
H	2.45308399	-2.22843	-5.4932351
H	2.38058496	-3.9538269	-5.9735289
C	0.240455	2.00661397	-0.198912
C	1.62913096	2.33593893	-0.23665
C	-0.695497	3.05675602	-0.196593
C	2.01079392	3.70214796	-0.107791
C	-0.304265	4.38997078	-0.197722
H	-1.7543581	2.81817698	-0.196102
C	1.05086899	4.71487093	-0.118848
H	-1.044413	5.185112	-0.217103
H	1.33446503	5.75625515	-0.044982
C	2.54107094	1.18104196	-0.429104
C	3.86591911	1.22990298	-0.886221
C	2.67769194	-1.1678881	-0.332077
C	4.6034708	0.056535	-1.0184079
H	4.31387901	2.17185307	-1.168805
C	4.01445007	-1.168558	-0.708541
H	2.14103889	-2.0880599	-0.132778
H	5.62968922	0.103324	-1.369688
H	4.55694914	-2.1037059	-0.78887
N	1.96267295	-0.036905	-0.216806
S	3.71265888	4.23087692	0.240749
O	4.25626898	3.35107088	1.29768097
O	4.48388815	4.36548424	-1.018747
C	3.60321307	5.88468599	0.95827198
H	3.293468	6.61463308	0.210453
H	2.93691802	5.87952614	1.82139099
H	4.62805414	6.09300995	1.27591503

Table S1(45). Optimized parameter of S_0 state of **1_5-SO$_2$Me**.

Atoms	X	Y	Z
Ir	0.10133	-0.164376	0.20573001
C	-1.908877	-0.370199	0.19683
C	-2.730525	-0.575941	-0.928685
C	-2.547904	-0.417815	1.47665298
C	-4.089694	-0.86664	-0.811651
H	-2.289623	-0.527891	-1.919771
C	-3.9087601	-0.771352	1.58742595
C	-4.6787562	-0.990386	0.45197099
H	-4.6872211	-1.025813	-1.7068191
H	-4.3652582	-0.91812	2.55798602
H	-5.7245131	-1.267493	0.550798
C	-1.68387	-0.181214	2.64228201
C	-2.0451701	0.115827	3.98642802
C	0.58266997	-0.234804	3.3159709
C	-1.072668	0.092054	4.98824406
C	0.262308	-0.122446	4.66066504
H	1.61173999	-0.300789	2.98181605
H	-1.335308	0.26459801	6.02414989
N	-0.348857	-0.263596	2.35327411
H	1.03433096	-0.145332	5.42050886
S	-3.702719	0.68970102	4.4684
O	-4.1611948	1.65955198	3.455899
O	-4.5686412	-0.464457	4.79360199
C	-3.46363	1.62162805	5.99685383
H	-2.7127299	2.39946294	5.85156107
H	-4.441916	2.07558489	6.17426014
H	-3.210819	0.96042103	6.8257308
C	0.38878	-0.214927	-1.7946
C	0.62251103	0.888035	-2.639118
C	0.47061199	-1.512201	-2.3935289
C	0.97409803	0.73436499	-3.9800279
H	0.54844302	1.89087296	-2.229708
C	0.88746101	-1.659073	-3.7329049
C	1.13373697	-0.544716	-4.5251989
H	1.15300798	1.61318302	-4.5959678
H	1.06182206	-2.6408119	-4.1538711
H	1.45882201	-0.671801	-5.553957
C	0.192689	-2.653132	-1.508513
C	-0.085962	-4.0081291	-1.844377
C	0.116103	-3.2572191	0.77741897
C	-0.125352	-4.9787769	-0.841305
C	0.011969	-4.6104579	0.49315
H	0.12777101	-2.8917091	1.79786396
H	-0.286159	-6.0220242	-1.081371
N	0.20366199	-2.323961	-0.180236
H	-0.0155	-5.3459148	1.28829396
S	-0.553637	-4.5444231	-3.5189791
O	-1.505528	-3.558296	-4.0644121
O	0.65501601	-4.8780241	-4.3036952
C	-1.478593	-6.0791369	-3.2955589
H	-0.823041	-6.890604	-2.9797361
H	-2.3041179	-5.9265208	-2.5992169
H	-1.8676029	-6.2891231	-4.2951822
C	0.16184901	1.84393895	0.42472899
C	1.462623	2.43441105	0.509996
C	-0.937558	2.70662999	0.60121399
C	1.61464	3.79018903	0.86768901
C	-0.777853	4.06036282	0.89701599
H	-1.942613	2.302845	0.524387
C	0.50300902	4.60207605	1.055686
H	2.59740996	4.20987988	1.04152501
H	-1.654115	4.69085789	1.03243005
H	0.63293898	5.64350319	1.33624005
C	2.60118604	1.52708995	0.307928
C	3.9658699	1.83861697	0.053073
C	3.18995309	-0.761899	0.38751099
C	4.93110609	0.83144403	0.115563
C	4.54868078	-0.489973	0.32343999
H	2.81748104	-1.778542	0.44152001
H	5.98090792	1.05737197	-0.022083
H	5.27915812	-1.288448	0.37576199
N	2.26107001	0.20367099	0.38105699
S	4.52203608	3.472332	-0.521948
O	3.55027294	3.95740795	-1.520139
O	4.85144281	4.33759403	0.631441
C	6.06211519	3.1654191	-1.414549
H	6.86559296	2.89416504	-0.729898
H	5.90769577	2.40724397	-2.183492
H	6.28512621	4.12908792	-1.87941

Table S1(46). Optimized parameter of T_1 state of **1**_*5*-**SO_2Me**.

Atoms	X	Y	Z
Ir	0.07895	-0.114408	0.156102
C	-1.9426661	-0.347593	0.209509
C	-2.79339	-0.547936	-0.891447
C	-2.530602	-0.431083	1.50841999
C	-4.1434522	-0.865677	-0.733222
H	-2.3871491	-0.474031	-1.8960789
C	-3.880441	-0.808773	1.66052198
C	-4.6849322	-1.020591	0.54710603
H	-4.7687898	-1.020485	-1.6096441
H	-4.3012381	-0.979354	2.64339495
H	-5.7220688	-1.316201	0.67689699
C	-1.63141	-0.209295	2.65288401
C	-1.958877	0.069668	4.00840092
C	0.65411502	-0.278716	3.27082801
C	-0.961403	0.025111	4.98476982
C	0.365302	-0.18928	4.62404823
H	1.67371404	-0.341298	2.90790892
H	-1.199424	0.181274	6.02936077
N	-0.30366	-0.288854	2.33442593
H	1.15388095	-0.227818	5.36584616
S	-3.6034961	0.64589602	4.53728008
O	-4.0860872	1.61506104	3.53625703
O	-4.4589071	-0.508575	4.8854208
C	-3.3148749	1.57733297	6.05710411
H	-2.5607779	2.34754896	5.88945198
H	-4.283392	2.04100204	6.26087999
H	-3.045852	0.91421098	6.87920904
C	0.34432501	-0.188497	-1.84743
C	0.569745	0.91568202	-2.689909
C	0.42247	-1.487241	-2.437994
C	0.91271198	0.75950903	-4.032589
H	0.48808599	1.917781	-2.281611
C	0.83224797	-1.633124	-3.7786231
C	1.07157004	-0.519629	-4.574564
H	1.08441401	1.63798499	-4.6502361
H	1.00780702	-2.6149759	-4.198009
H	1.38946402	-0.648865	-5.6050348
C	0.15644801	-2.6301069	-1.549875
C	-0.122679	-3.9853771	-1.8826129
C	0.093611	-3.2270081	0.73782599
C	-0.161145	-4.951273	-0.87533
C	-0.018355	-4.5799441	0.45777801
H	0.114917	-2.8594229	1.75725603
H	-0.325104	-5.9949589	-1.110821
N	0.180815	-2.3001339	-0.225691
H	-0.044605	-5.313148	1.25489795
S	-0.587823	-4.5276079	-3.5572939
O	-1.552424	-3.550905	-4.0963311
O	0.62475199	-4.8419652	-4.3428931
C	-1.489803	-6.0751271	-3.3332839
H	-0.821609	-6.8763361	-3.017638
H	-2.317971	-5.9348211	-2.6375451
H	-1.875119	-6.2909379	-4.3331199
C	0.101338	1.85254502	0.30301899
C	1.45292795	2.44886804	0.441331
C	-1.017025	2.71687698	0.33596301
C	1.54066098	3.83722305	0.73796302
C	-0.891726	4.08149099	0.55659401
H	-2.0048871	2.28759789	0.20295601
C	0.402428	4.623106	0.78184098
H	2.50060391	4.29137278	0.949875
H	-1.764918	4.72605801	0.58439702
H	0.50429398	5.68330479	1.00200605
C	2.5632081	1.57120001	0.314567
C	3.98436904	1.87347496	0.188637
C	3.1385119	-0.730353	0.52436501
C	4.91837978	0.87970501	0.406349
C	4.50398302	-0.443687	0.65641803
H	2.78239703	-1.755919	0.55169702
H	5.98051596	1.08329904	0.331662
H	5.22037411	-1.235899	0.83467197
N	2.21542001	0.204712	0.34613401
S	4.56138802	3.43449998	-0.493339
O	3.66588593	3.80750489	-1.610196
O	4.77201796	4.43102121	0.58527303
C	6.18879986	3.11352491	-1.211876
H	6.91875601	2.8895359	-0.433638
H	6.12425709	2.31100702	-1.9474911
H	6.45298386	4.05415297	-1.702075

Table S1(47). Optimized parameter of S_0 state of **1**_*6*-**SO_2Me**.

Atoms	X	Y	Z
Ir	-0.217312	0.187314	-0.159817
C	-2.2414391	0.192974	-0.008218
C	-3.185792	0.150777	-1.052726
C	-2.766041	0.233283	1.31824899
C	-4.5601749	0.14814501	-0.808761
H	-2.8366859	0.123935	-2.0807071
C	-4.153297	0.227431	1.561396
C	-5.0519729	0.18548501	0.502491
H	-5.2557569	0.116352	-1.64472
H	-4.5379152	0.25420901	2.5770781
H	-6.1214089	0.180351	0.69291598
C	-1.785316	0.288059	2.40480208
C	-2.095855	0.383468	3.7732501
C	0.50652897	0.31121901	2.92196298
C	-1.064682	0.43692699	4.69722414
H	-3.12411	0.43436399	4.10812807
C	0.26957199	0.404459	4.28715611
H	1.51772106	0.28784099	2.53282094
N	-0.479885	0.252038	2.01033306
H	1.09009802	0.46720901	4.99152088
S	-1.4603699	0.58116001	6.45422983
O	-0.353943	1.31347597	7.09954882
O	-2.8372409	1.10012996	6.56268215
C	-1.443978	-1.1187381	7.04881096
H	-2.2063551	-1.6884741	6.51480198
H	-0.451304	-1.543388	6.88993883
H	-1.675133	-1.076023	8.11626339
C	-0.161154	-0.035579	-2.176136
C	-0.100087	0.97391301	-3.156791
C	-0.176618	-1.380843	-2.652761
C	-0.057548	0.680197	-4.5207691
H	-0.09118	2.01431298	-2.845706
C	-0.131026	-1.6740331	-4.0295172
C	-0.071885	-0.648296	-4.9649539
H	-0.012795	1.49058604	-5.2452712
H	-0.141279	-2.7030411	-4.3779521
H	-0.036448	-0.877644	-6.026145
C	-0.251651	-2.4317119	-1.634989
C	-0.318044	-3.8121121	-1.897349
C	-0.350431	-2.8640051	0.672575
C	-0.396523	-4.6978321	-0.834671
H	-0.324853	-4.1862769	-2.913172
C	-0.420052	-4.2378821	0.48366699
H	-0.368161	-2.4371231	1.66859305
N	-0.264865	-1.98911	-0.344706
H	-0.505678	-4.9114299	1.32769799
S	-0.498389	-6.4707561	-1.167647
O	-1.284465	-7.082118	-0.078935
O	-0.937898	-6.6401162	-2.565815
C	1.20402205	-7.0428772	-1.03134
H	1.81102002	-6.5383511	-1.7851501
H	1.57211006	-6.8347492	-0.02516
H	1.18310201	-8.1201544	-1.214947
C	-0.001188	2.20567703	-0.162284
C	1.34255195	2.68669701	-0.148826
C	-1.014696	3.184273	-0.163156
C	1.63039899	4.06530523	-0.133069
C	-0.726165	4.54995823	-0.151089
H	-2.054142	2.87020898	-0.178124
C	0.60085499	4.99836302	-0.134799
H	2.65823197	4.41701317	-0.11878
H	-1.539528	5.27250719	-0.153914
H	0.82610202	6.06095219	-0.122284
C	2.39818192	1.67123902	-0.161565
C	3.77927208	1.936854	-0.194119
C	2.84031701	-0.636465	-0.178453
C	4.67001581	0.87581599	-0.212919
H	4.15091801	2.95328403	-0.220495
C	4.21509504	-0.444456	-0.211613
H	2.41732597	-1.634265	-0.179532
H	4.89338779	-1.28815	-0.251224
N	1.96012199	0.37945101	-0.148863
S	6.444242	1.21212494	-0.262568
O	7.08415604	0.112997	-1.010507
O	6.62565517	2.60371208	-0.717833
C	6.95612717	1.10300601	1.46066904
H	6.42817783	1.864766	2.03694296
H	6.735744	0.102191	1.83622897
H	8.03312206	1.28855205	1.47616601

Table S1(48). Optimized parameter of T_1 state of **1**_*6*-**SO$_2$Me**.

Atoms	X	Y	Z
Ir	-0.262377	0.193368	-0.200159
C	-2.2743361	0.14942899	-0.019268
C	-3.212451	0.04958	-1.064581
C	-2.787111	0.20495699	1.31007004
C	-4.5844102	0.014033	-0.814561
H	-2.8627639	0.017342	-2.091517
C	-4.17133	0.16034301	1.554353
C	-5.0699568	0.06754	0.497621
H	-5.2809949	-0.054542	-1.646535
H	-4.5548768	0.195159	2.569453
H	-6.1378088	0.036754	0.69241101
C	-1.804578	0.29755199	2.39305401
C	-2.1150739	0.40401399	3.76005507
C	0.48712701	0.40081501	2.89818692
C	-1.081882	0.50464302	4.67749023
H	-3.142983	0.42489699	4.09889603
C	0.250696	0.51182199	4.26211596
H	1.49724996	0.400226	2.5063529
N	-0.502366	0.290299	1.99628401
H	1.071244	0.61314601	4.96189308
S	-1.475997	0.66277802	6.4366622
O	-0.390078	1.440153	7.06249523
O	-2.8677809	1.14004505	6.53884888
C	-1.403229	-1.026902	7.05522203
H	-2.148943	-1.629037	6.53331614
H	-0.397977	-1.422263	6.89997482
H	-1.632383	-0.974384	8.12271309
C	-0.200204	-0.000565	-2.1782451
C	-0.122499	1.04056799	-3.1344621
C	-0.233305	-1.371249	-2.66927
C	-0.111276	0.779778	-4.496335
H	-0.093669	2.06891489	-2.789742
C	-0.218794	-1.6119731	-4.0623078
C	-0.160921	-0.55713	-4.9570322
H	-0.069144	1.59571898	-5.2123389
H	-0.248534	-2.6276231	-4.444324
H	-0.150185	-0.759531	-6.0246038
C	-0.257468	-2.414593	-1.676266
C	-0.304061	-3.7831581	-1.937927
C	-0.339139	-2.855845	0.66579998
C	-0.374966	-4.7043309	-0.892063
H	-0.29956	-4.143456	-2.960367
C	-0.401883	-4.2109251	0.46389899
H	-0.364646	-2.4483049	1.67117095
N	-0.243153	-1.941792	-0.339606
H	-0.493304	-4.8865032	1.30598998
S	-0.38711	-6.4238572	-1.22263
O	-1.2152391	-7.100709	-0.195467
O	-0.713835	-6.6327591	-2.6533091
C	1.30732703	-7.006702	-0.977984
H	1.958673	-6.516912	-1.704264
H	1.62046003	-6.7725272	0.041645
H	1.30564296	-8.0888729	-1.134047
C	0.000028	2.22314596	-0.161345
C	1.35385895	2.66784191	-0.147607
C	-0.995644	3.2143631	-0.128734
C	1.66919196	4.03963804	-0.111204
C	-0.67886	4.57431602	-0.096632
H	-2.040925	2.9195621	-0.138749
C	0.65718597	4.99214983	-0.088467
H	2.70299911	4.37223387	-0.098574
H	-1.476282	5.31360817	-0.07936
H	0.90489799	6.0492692	-0.062218
C	2.39216805	1.63117898	-0.16149
C	3.77828193	1.87238896	-0.186269
C	2.79566002	-0.689729	-0.175208
C	4.647861	0.79437399	-0.201489
H	4.16946077	2.88135505	-0.209321
C	4.17298222	-0.519167	-0.201664
H	2.34471989	-1.675574	-0.177138
H	4.83906317	-1.372617	-0.23807
N	1.94005501	0.345716	-0.152948
S	6.43137598	1.10153699	-0.250004
O	7.04887676	-0.008752	-0.998949
O	6.63208723	2.48974395	-0.705652
C	6.93779182	0.98333001	1.47374904
H	6.42342186	1.75509405	2.0489521
H	6.6994009	-0.013346	1.84923398
H	8.01796246	1.14947402	1.48970199

Table S1(49). Optimized parameter of S_0 state of **1_7-SO$_2$Me**.

Atoms	X	Y	Z
Ir	-0.14817	0.103739	0.031607
C	-2.16786	0.222911	0.177627
C	-3.113658	0.17872401	-0.866787
C	-2.692554	0.381715	1.49708605
C	-4.484221	0.292171	-0.632285
H	-2.7682509	0.061207	-1.8890361
C	-4.0783539	0.49659401	1.72911298
C	-4.9752889	0.45446599	0.67024201
H	-5.1782942	0.25564599	-1.469298
H	-4.462049	0.61927998	2.73770189
H	-6.0423431	0.54325497	0.85288
C	-1.721503	0.40323299	2.58897209
C	-2.0353031	0.51823199	3.95731401
C	0.556023	0.22821499	3.12084103
C	-1.034711	0.464885	4.91309118
H	-3.0647321	0.64025998	4.27025414
C	0.28649601	0.298363	4.48198605
H	1.571998	0.127767	2.75905609
H	-1.2690671	0.552858	5.96874285
N	-0.41613	0.28019601	2.19974494
S	1.63331902	0.154359	5.65179491
O	2.89260507	0.36972001	4.91178083
O	1.31981504	1.00750506	6.81443596
C	1.57300603	-1.5669481	6.18397379
H	0.63658798	-1.7393791	6.71761513
H	1.64737403	-2.218926	5.3112731
H	2.42526603	-1.717267	6.85160589
C	-0.145301	-0.276619	-1.958582
C	-0.109488	0.65535003	-3.0155001
C	-0.214505	-1.654474	-2.332566
C	-0.149719	0.260903	-4.3531189
H	-0.060403	1.71545005	-2.785197
C	-0.257539	-2.0478849	-3.6856821
C	-0.227548	-1.09602	-4.696094
H	-0.123352	1.01422799	-5.1376238
H	-0.315937	-3.0987239	-3.954845
H	-0.261601	-1.4023449	-5.7376609
C	-0.207446	-2.6278889	-1.242141
C	-0.189056	-4.028553	-1.393373
C	-0.054053	-2.87377	1.08585095
C	-0.066804	-4.8519158	-0.28603
H	-0.24941	-4.4680929	-2.381115
C	0.030645	-4.2553978	0.97736901
H	-0.014921	-2.3891799	2.05355191
H	-0.041037	-5.9308872	-0.398339
N	-0.175335	-2.082459	0.011336
S	0.31734499	-5.2246022	2.45478511
O	0.12271	-4.333302	3.61715198
O	-0.474123	-6.4660869	2.35492206
C	2.06467295	-5.6553249	2.35623002
H	2.21986389	-6.3063479	1.49378395
H	2.65979505	-4.7439442	2.26561189
H	2.306463	-6.1863799	3.28046703
C	0.15370101	2.1010561	-0.137643
C	1.51791096	2.5287571	-0.123442
C	-0.816666	3.11517191	-0.265736
C	1.86116099	3.89190292	-0.231254
C	-0.471465	4.46273708	-0.372518
H	-1.867118	2.84124207	-0.28798
C	0.87276101	4.85911417	-0.35568
H	2.90186191	4.20302916	-0.220328
H	-1.25333	5.21289921	-0.470363
H	1.14143801	5.90835285	-0.437834
C	2.53243399	1.48418796	0.008314
C	3.9235301	1.69683194	0.073487
C	2.87189198	-0.826423	0.22950201
C	4.79069901	0.62780499	0.230371
H	4.3215332	2.70218301	0.016819
C	4.24860382	-0.660676	0.308227
H	2.42549109	-1.810396	0.30043101
H	5.86147594	0.78493899	0.305565
N	2.037431	0.211817	0.088184
S	5.28773212	-2.0974491	0.55965298
O	6.47735405	-1.662573	1.31601095
O	4.43358612	-3.1729651	1.10515499
C	5.81555605	-2.5852001	-1.091652
H	4.93527603	-2.8311701	-1.687834
H	6.37874317	-1.766165	-1.5424581
H	6.45315409	-3.464324	-0.96826

Table S1(50). Optimized parameter of T_1 state of **1**_*7*-**SO$_2$Me**.

Atoms	X	Y	Z
Ir	-0.204955	0.112463	0.02901
C	-2.172256	0.234102	0.151563
C	-3.109335	0.166829	-0.908062
C	-2.691855	0.43382901	1.52010798
C	-4.4745188	0.30504501	-0.698058
H	-2.74171	0.019036	-1.9177721
C	-4.0900021	0.57854801	1.70324802
C	-4.9532108	0.51597202	0.620911
H	-5.1729941	0.258205	-1.527831
H	-4.5009351	0.73565799	2.69542599
H	-6.0214381	0.628232	0.78939998
C	-1.746977	0.444765	2.58493996
C	-2.042531	0.57665598	3.97345996
C	0.55141598	0.21456	3.10527802
C	-1.054224	0.51344502	4.92134809
H	-3.0708051	0.72613502	4.28489685
C	0.29545099	0.31290901	4.47709322
H	1.56826794	0.081306	2.75255704
H	-1.277663	0.62370002	5.97656822
N	-0.404296	0.27886099	2.18671203
S	1.61088502	0.080754	5.61667681
O	2.89252996	0.177073	4.87995005
O	1.39308298	0.96289802	6.78618002
C	1.45006096	-1.6203721	6.20590878
H	0.491721	-1.726061	6.71854401
H	1.50611997	-2.30421	5.35614681
H	2.27341008	-1.799974	6.90240002
C	-0.173449	-0.286129	-1.9707201
C	-0.146117	0.63653803	-3.0308759
C	-0.196213	-1.668963	-2.322084
C	-0.154752	0.22509199	-4.365294
H	-0.130595	1.70015097	-2.8111429
C	-0.207762	-2.0792611	-3.670315
C	-0.189921	-1.136811	-4.6906381
H	-0.138157	0.968961	-5.1585379
H	-0.231865	-3.1337199	-3.928951
H	-0.200775	-1.456412	-5.7285328
C	-0.168742	-2.6310051	-1.219301
C	-0.109868	-4.0318551	-1.355165
C	-0.030242	-2.8514471	1.11932898
C	0.02164	-4.8382912	-0.236371
H	-0.1473	-4.4843512	-2.337956
C	0.090219	-4.2299681	1.02398002
H	-0.015003	-2.3493011	2.07901692
H	0.0768	-5.9172549	-0.338836
N	-0.160918	-2.0795541	0.031515
S	0.37500501	-5.1823068	2.516155
O	0.174464	-4.274508	3.66392303
O	-0.415906	-6.4242768	2.42742991
C	2.12301493	-5.6096268	2.42448902
H	2.27961707	-6.2784681	1.57609999
H	2.71490502	-4.6985288	2.31365609
H	2.36659789	-6.1200328	3.35979795
C	0.138273	2.10802007	-0.104952
C	1.50310302	2.51962709	-0.09099
C	-0.832715	3.12249494	-0.199062
C	1.84932196	3.88306308	-0.169764
C	-0.480746	4.47043514	-0.278191
H	-1.883358	2.85170197	-0.222247
C	0.864528	4.85759306	-0.264196
H	2.89093399	4.18903208	-0.159019
H	-1.260489	5.22482109	-0.351809
H	1.13878906	5.90653181	-0.325191
C	2.51518011	1.46839404	0.012587
C	3.90591502	1.67934597	0.075155
C	2.85167789	-0.846906	0.164105
C	4.77153111	0.60438699	0.195272
H	4.30499792	2.68515205	0.046365
C	4.2289629	-0.684969	0.23625299
H	2.40628505	-1.832419	0.210769
H	5.84284496	0.758205	0.26958099
N	2.02006102	0.19756	0.062307
S	5.26936817	-2.1299429	0.44657499
O	6.46012688	-1.712942	1.21005702
O	4.41367388	-3.216073	0.96706003
C	5.79106188	-2.575985	-1.2183141
H	4.90896177	-2.8026309	-1.819328
H	6.35827112	-1.749109	-1.649308
H	6.424191	-3.4612229	-1.117728

Table S1(51). Optimized parameter of S_0 state of **1**_*2*-**NO₂**.

Atoms	X	Y	Z
Ir	-0.02157	-0.007975	0.030208
C	-2.0404739	0.024216	0.209526
C	-2.9927349	0.002517	-0.823478
C	-2.5488	0.067756	1.54320896
C	-4.3579722	0.020603	-0.537559
H	-2.6809759	-0.025142	-1.859319
C	-3.931205	0.082525	1.80595803
C	-4.8519869	0.059721	0.76880401
H	-4.3033738	0.111655	2.82474208
H	-5.9180422	0.07063	0.95339203
C	-1.548934	0.09815	2.62365007
C	-1.8377121	0.16559801	3.99384809
C	0.74406803	0.078783	3.09959793
C	-0.802094	0.187556	4.92193794
H	-2.865835	0.20311201	4.33314514
C	0.51769799	0.142037	4.46982718
H	1.74918997	0.04435	2.69530511
H	-1.022732	0.240436	5.98346615
N	-0.254263	0.055985	2.20005393
H	1.35658205	0.15747499	5.15664482
N	-5.3153801	-0.002775	-1.6457011
O	-6.5210252	0.010011	-1.376823
O	-4.879139	-0.034981	-2.800185
C	0.006436	-0.250149	-1.982025
C	0.056765	0.75183803	-2.965827
C	-0.024887	-1.599455	-2.448699
C	0.076252	0.42278999	-4.3212881
H	0.076693	1.79734302	-2.6875479
C	-0.001739	-1.905856	-3.821974
C	0.048596	-0.898551	-4.7743449
H	-0.022861	-2.9357979	-4.1629338
H	0.066814	-1.117148	-5.833849
C	-0.085666	-2.6477909	-1.416337
C	-0.147254	-4.0263028	-1.663922
C	-0.135076	-3.0513899	0.89009702
C	-0.20163	-4.921392	-0.600778
H	-0.155296	-4.3971548	-2.6816399
C	-0.194418	-4.4279618	0.70497602
H	-0.130291	-2.6156869	1.88266897
H	-0.250164	-5.989275	-0.789377
N	-0.080736	-2.1838141	-0.134908
H	-0.236225	-5.0879631	1.56423998
N	0.12750299	1.50027597	-5.3120179
O	0.14424799	1.19389796	-6.5086622
O	0.152464	2.6677351	-4.911212
C	0.221717	2.00399899	0.003009
C	1.57170606	2.46988201	-0.002201
C	-0.780611	2.98882699	-0.007773
C	1.87820804	3.84334898	-0.011149
C	-0.451333	4.34430599	-0.019462
H	-1.826485	2.71117401	-0.011329
C	0.87055802	4.79660606	-0.02022
H	2.908566	4.18377304	-0.011118
C	2.62078309	1.43653905	-0.000624
C	4.0007062	1.68232596	-0.029872
C	3.0240829	-0.870205	0.035952
C	4.89625216	0.61820298	-0.023698
H	4.3724041	2.69930291	-0.059925
C	4.40193796	-0.686767	0.011343
H	2.58778191	-1.862223	0.060391
H	5.96522522	0.80542099	-0.047024
H	5.06229305	-1.546759	0.016867
N	2.1560719	0.15581501	0.031402
H	1.08926702	5.85621881	-0.027173
N	-1.529123	5.33594084	-0.031351
O	-1.222528	6.53263378	-0.036166
O	-2.697088	4.93583298	-0.035479

Table S1(52). Optimized parameter of T_1 state of **1**_*2*-**NO₂**.

Atoms	X	Y	Z
Ir	-0.0242	0.06557	0.044632
C	-2.052906	0.073788	0.21981201
C	-2.996984	0.087002	-0.815044
C	-2.5506899	0.050175	1.55407596
C	-4.361928	0.077139	-0.525783
H	-2.6855371	0.110629	-1.851
C	-3.9321151	0.039385	1.81949496
C	-4.8521891	0.053399	0.78108698
H	-4.303834	0.021118	2.8382411
H	-5.9180741	0.046183	0.96690398
C	-1.547197	0.037088	2.63198495
C	-1.829833	0.034593	4.00413704
C	0.75540298	0.013506	3.10125995
C	-0.789039	0.020981	4.92680883
H	-2.8561599	0.045266	4.34973478
C	0.53110898	0.009868	4.47189999
H	1.75755298	0.006516	2.68903804
H	-1.0064811	0.019819	5.99014521
N	-0.250801	0.026131	2.20912004
H	1.37053394	0.001408	5.15764713
N	-5.3214798	0.093069	-1.633819
O	-6.5254269	0.082314	-1.361782
O	-4.8841701	0.115255	-2.787318
C	0.028412	-0.232019	-1.97144
C	0.107056	0.76301801	-2.9561639
C	-0.010548	-1.584427	-2.4129
C	0.143536	0.41853499	-4.3073402
H	0.130187	1.81087506	-2.6884511
C	0.029711	-1.9029731	-3.7825589
C	0.106018	-0.906087	-4.7440891
H	0.001904	-2.9355519	-4.1129718
H	0.136114	-1.137048	-5.8007102
C	-0.093273	-2.623472	-1.372528
C	-0.165669	-4.0018158	-1.61323
C	-0.172106	-3.0101719	0.93543702
C	-0.240721	-4.8884692	-0.544193
H	-0.165668	-4.379426	-2.6281049
C	-0.242901	-4.3870578	0.75796998
H	-0.171942	-2.5714071	1.92606199
H	-0.297531	-5.9568791	-0.726361
N	-0.097586	-2.1525359	-0.095997
H	-0.299466	-5.040596	1.62102699
N	0.22228099	1.48711395	-5.3072829
O	0.247675	1.16751397	-6.4991722
O	0.26016799	2.65576696	-4.9137421
C	0.188774	2.01821303	-0.02034
C	1.59278595	2.47953701	-0.074448
C	-0.828461	2.98770308	-0.017487
C	1.86033404	3.86726308	-0.125945
C	-0.532893	4.3578229	-0.066369
H	-1.868039	2.69324994	0.013169
C	0.83766699	4.78961611	-0.124364
H	2.88221097	4.23042679	-0.165187
C	2.61694598	1.47068202	-0.047996
C	4.00922585	1.71018696	-0.097225
C	3.02177095	-0.844667	0.055914
C	4.89594984	0.646613	-0.070186
H	4.38144112	2.72576404	-0.157404
C	4.40044785	-0.662942	0.007332
H	2.58755994	-1.836351	0.118265
H	5.96530485	0.82915699	-0.109407
H	5.06048918	-1.522397	0.029304
N	2.15601802	0.178257	0.034471
H	1.03952706	5.85207987	-0.16535
N	-1.555716	5.30791092	-0.062237
O	-1.243815	6.54246378	-0.106735
O	-2.7655261	4.92094517	-0.013024

Table S1(53). Optimized parameter of S_0 state of **1**_*3*-**NO_2**.

Atoms	X	Y	Z
Ir	-0.028879	-0.005802	0.030951
C	-2.0372679	0.032261	0.21665999
C	-3.000402	0.013626	-0.817445
C	-2.536474	0.070847	1.55826998
C	-4.3656468	0.028522	-0.564122
H	-2.6699641	-0.009552	-1.850484
C	-3.9070449	0.082698	1.82537603
C	-4.8084941	0.061849	0.76329201
H	-4.2991362	0.106547	2.83413291
C	-1.533699	0.097522	2.636796
C	-1.8168531	0.16040701	4.008111
C	0.76193798	0.076083	3.10258007
C	-0.776706	0.178719	4.93058777
H	-2.8436799	0.19672699	4.35184002
C	0.54192001	0.13474301	4.47307396
H	1.76467502	0.042731	2.69252896
H	-0.992554	0.227781	5.99330091
N	-0.242379	0.056297	2.20851707
H	1.38342798	0.14780299	5.1565938
H	-5.089437	0.014281	-1.370213
N	-6.2323012	0.072976	1.043414
O	-6.6020069	0.101888	2.22426009
O	-7.0188422	0.052666	0.088634
C	-0.006071	-0.255762	-1.970525
C	0.041587	0.74628299	-2.9660411
C	-0.033	-1.613029	-2.4266169
C	0.064226	0.448722	-4.3222179
H	0.056438	1.78978395	-2.6696761
C	-0.007521	-1.924485	-3.7876301
C	0.040598	-0.892377	-4.7222862
H	-0.023346	-2.945276	-4.1479831
C	-0.090717	-2.658596	-1.390554
C	-0.148011	-4.0382142	-1.631379
C	-0.138738	-3.050231	0.918567
C	-0.199198	-4.9267492	-0.563113
H	-0.154696	-4.4141288	-2.6473839
C	-0.1937	-4.4270072	0.740915
H	-0.135052	-2.6079631	1.90816605
H	-0.244109	-5.9958038	-0.745969
N	-0.086986	-2.1891651	-0.112988
H	-0.23337	-5.0830379	1.60320997
H	0.099793	1.23098505	-5.0709682
N	0.067658	-1.218698	-6.1359959
O	0.046474	-2.4108911	-6.4678621
O	0.111249	-0.290022	-6.9522462
C	0.22258499	1.99502897	-0.002311
C	1.58042097	2.45044708	-0.003076
C	-0.779729	2.99146605	-0.015807
C	1.89185703	3.81173396	-0.009177
C	-0.482056	4.34774303	-0.025461
H	-1.823413	2.69542408	-0.023496
C	0.85944003	4.74720812	-0.020388
H	2.9128809	4.17181921	-0.004583
C	2.62658	1.41342294	0.001823
C	4.00747585	1.652367	-0.024948
C	3.01762605	-0.895965	0.042386
C	4.89624786	0.58312303	-0.015195
H	4.384305	2.66756701	-0.055554
C	4.39554977	-0.720055	0.020522
H	2.57473993	-1.88498	0.067437
H	5.96627808	0.764548	-0.036413
H	5.05177402	-1.583079	0.028623
N	2.15633798	0.13662	0.034641
H	-1.26451	5.09706116	-0.036229
N	1.18584502	6.16111803	-0.025301
O	0.25691301	6.97817707	-0.035819
O	2.37838197	6.49239588	-0.018396

Table S1(54). Optimized parameter of T_1 state of **1**_*3*-**NO_2**.

Atoms	X	Y	Z
Ir	-0.026316	0.050901	0.046002
C	-2.0444019	0.076815	0.22270501
C	-2.9951041	0.098057	-0.816769
C	-2.5401299	0.059429	1.56174397
C	-4.362689	0.101247	-0.566385
H	-2.660563	0.11798	-1.84828
C	-3.912461	0.05968	1.82526302
C	-4.8068972	0.080841	0.75894397
H	-4.3084741	0.044508	2.83239794
C	-1.539236	0.041791	2.64183593
C	-1.8234561	0.04015	4.01362705
C	0.761733	0.012455	3.11167598
C	-0.783289	0.023199	4.93619204
H	-2.8502989	0.053401	4.35826111
C	0.53755599	0.008764	4.48155499
H	1.76440406	0.003322	2.700881
H	-1.0009919	0.021997	5.99950409
N	-0.244686	0.027259	2.21855998
H	1.37661397	-0.002364	5.16769886
H	-5.0838742	0.119467	-1.374465
N	-6.2364068	0.0809	1.03516698
O	-6.606844	0.059444	2.21406603
O	-7.0162978	0.101925	0.077298
C	0.01538	-0.24939	-1.960042
C	0.088834	0.74553603	-2.9570079
C	-0.02457	-1.608223	-2.3930061
C	0.122515	0.432666	-4.3107662
H	0.112955	1.79063404	-2.668915
C	0.010919	-1.931919	-3.7520461
C	0.083526	-0.910347	-4.6940589
H	-0.015811	-2.955646	-4.1023612
C	-0.103414	-2.644274	-1.348789
C	-0.17497	-4.023777	-1.581914
C	-0.173341	-3.0184209	0.96212798
C	-0.244912	-4.9037709	-0.507546
H	-0.177882	-4.40657	-2.595057
C	-0.243094	-4.3954248	0.79267401
H	-0.170305	-2.5725679	1.94955301
H	-0.301	-5.9733071	-0.683439
N	-0.103699	-2.166616	-0.075826
H	-0.295923	-5.0446811	1.65916002
H	0.17754699	1.207618	-5.0656118
N	0.11993	-1.253244	-6.108922
O	0.08625	-2.447484	-6.4246969
O	0.183797	-0.333275	-6.9306221
C	0.21124899	2.01058507	-0.01197
C	1.59256101	2.48141694	-0.04574
C	-0.803123	3.02162004	-0.011334
C	1.89788401	3.82990098	-0.063377
C	-0.513788	4.36492109	-0.034412
H	-1.843692	2.71487689	-0.000234
C	0.85372901	4.7917552	-0.057129
H	2.91224289	4.20456219	-0.076722
C	2.63520908	1.44646597	-0.040181
C	4.01395798	1.68751097	-0.100589
C	3.02298999	-0.862056	0.034355
C	4.90272713	0.61713803	-0.091539
H	4.38633204	2.70283294	-0.156855
C	4.40198278	-0.683672	-0.022378
H	2.58291698	-1.850979	0.088719
H	5.97189808	0.79669797	-0.139055
H	5.0580039	-1.546662	-0.01403
N	2.16546893	0.170048	0.028556
H	-1.28716	5.12119198	-0.035651
N	1.16531205	6.14983797	-0.072467
O	0.199187	6.99939013	-0.055058
O	2.40271711	6.50307703	-0.103921

Table S1(55). Optimized parameter of S_0 state of **1_*4*-NO$_2$**.

Atoms	X	Y	Z
Ir	0.026297	-0.054942	0.042178
C	-2.001519	-0.070615	0.063467
C	-2.8514471	-0.123345	-1.062023
C	-2.6110859	-0.079372	1.35405695
C	-4.2381959	-0.184006	-0.950682
H	-2.4090519	-0.110571	-2.05302
C	-4.0130229	-0.246611	1.43304598
C	-4.8324971	-0.261967	0.30916101
H	-4.8616781	-0.206714	-1.840138
H	-5.9025679	-0.38274	0.42839301
C	-1.705174	0.17017099	2.49587703
C	-2.079453	0.54786497	3.79491496
C	0.55527002	0.30992699	3.11673903
C	-1.10773	0.77274299	4.76418781
C	0.23926	0.62915099	4.43088722
H	1.58413303	0.228214	2.78542709
N	-0.38159	0.101809	2.17585397
H	1.02869296	0.78622699	5.15729284
H	-3.118876	0.69005501	4.05361319
H	-1.402801	1.06340802	5.76760292
N	-4.706646	-0.553862	2.69172096
O	-5.776485	0.015499	2.91932011
O	-4.2080421	-1.405609	3.43141294
C	0.249795	-0.340811	-1.951566
C	0.372832	0.66378897	-2.934999
C	0.34107199	-1.70008	-2.379103
C	0.57755798	0.372722	-4.2812371
H	0.29939801	1.70343196	-2.6326351
C	0.65212101	-1.957988	-3.7341869
C	0.73487902	-0.951541	-4.6909361
H	0.96722901	-1.206933	-5.7178679
C	0.017958	-2.719568	-1.357625
C	-0.301	-4.0665522	-1.590353
C	-0.345346	-3.040798	0.94020498
C	-0.611845	-4.9049869	-0.525165
C	-0.61271	-4.3932419	0.77257401
H	-0.376954	-2.5743749	1.91808295
N	-0.053663	-2.2264431	-0.088354
H	-0.841556	-5.0128751	1.63236701
H	0.65136802	1.17274702	-5.0126019
H	-0.857098	-5.9460011	-0.710889
H	-0.330912	-4.4640989	-2.594559
N	1.04775	-3.287493	-4.21982
O	0.59813398	-3.66711	-5.3034639
O	1.85146403	-3.933188	-3.542793
C	0.250429	1.96036994	0.028751
C	1.58356798	2.43870711	0.20781
C	-0.778525	2.91961408	-0.085938
C	1.78224301	3.82940006	0.36793599
C	-0.534872	4.28942299	-0.026694
H	-1.7986391	2.57884097	-0.231449
C	0.75648302	4.75697184	0.218522
H	-1.350493	4.99893808	-0.13498
H	0.96696597	5.81249619	0.342399
C	2.64992309	1.41753995	0.127496
C	4.01932192	1.65129995	-0.073268
C	3.04985309	-0.896095	0.09404
C	4.90566683	0.58281898	-0.155965
C	4.42016077	-0.720468	-0.047503
H	2.60762405	-1.884618	0.13878401
H	5.07626915	-1.582147	-0.098172
N	2.18993592	0.13448501	0.165176
H	4.39952183	2.65622997	-0.187199
H	5.96386814	0.76938498	-0.30999
N	3.0517211	4.4020648	0.83731103
O	3.46077991	5.43037224	0.29337299
O	3.61589408	3.85346198	1.78703296

Table S1(56). Optimized parameter of T_1 state of **1**_*4*-**NO$_2$**.

Atoms	X	Y	Z
Ir	0.010117	-0.072143	-0.017551
C	-2.0341311	-0.067281	0.065395
C	-2.900382	-0.141087	-1.043299
C	-2.611779	-0.046581	1.36511397
C	-4.2847829	-0.196581	-0.901662
H	-2.480629	-0.14452	-2.043143
C	-4.0114598	-0.207007	1.47532105
C	-4.8532519	-0.24558	0.369959
H	-4.9234371	-0.236395	-1.7790771
H	-5.921227	-0.358077	0.51315397
C	-1.686757	0.21244	2.48784304
C	-2.0376639	0.634179	3.77854991
C	0.58226001	0.29846999	3.079108
C	-1.048328	0.85306603	4.73121214
C	0.28919101	0.65745902	4.38811493
H	1.60526705	0.17791501	2.74408007
N	-0.373017	0.097659	2.1552031
H	1.09117901	0.80579901	5.1021781
H	-3.0696249	0.81657302	4.04171705
H	-1.322543	1.17868304	5.72958803
N	-4.6767888	-0.480795	2.75811911
O	-5.7326512	0.106851	2.99843693
O	-4.1681371	-1.3240941	3.49911404
C	0.113978	-0.32501	-1.960838
C	0.007395	0.686436	-2.948339
C	0.352171	-1.709074	-2.3843141
C	0.173695	0.38001901	-4.3002291
H	-0.207475	1.70419204	-2.645864
C	0.779387	-1.930966	-3.7365
C	0.56623	-0.898527	-4.6904092
H	0.76078701	-1.131772	-5.7294111
C	0.049878	-2.720993	-1.394264
C	-0.204614	-4.0878782	-1.648984
C	-0.403501	-3.067888	0.891541
C	-0.536603	-4.9345441	-0.601453
C	-0.620301	-4.429327	0.70087999
H	-0.488818	-2.613384	1.87220299
N	-0.091165	-2.241709	-0.115529
H	-0.869509	-5.061687	1.545421
H	0.04825	1.15032196	-5.0558
H	-0.734625	-5.984056	-0.797231
H	-0.141109	-4.4661069	-2.657203
N	1.47949004	-3.0633149	-4.1643262
O	1.65594995	-3.2421279	-5.4153109
O	1.96098495	-3.873347	-3.30551
C	0.26145101	1.95214605	-0.025815
C	1.59647405	2.40009689	0.183099
C	-0.752009	2.91855192	-0.161857
C	1.81704903	3.78776193	0.33657101
C	-0.486406	4.2849741	-0.100927
H	-1.7735519	2.59250998	-0.32801
C	0.80832398	4.72956181	0.16349199
H	-1.287408	5.00764084	-0.22606
H	1.03504503	5.78234386	0.281376
C	2.64368296	1.35428703	0.150447
C	4.02122307	1.55083597	-0.019488
C	2.99509501	-0.975581	0.200471
C	4.88384199	0.45933601	-0.045985
C	4.37093592	-0.830718	0.091575
H	2.52658701	-1.950315	0.266478
H	5.01098394	-1.7053601	0.086484
N	2.16128802	0.079291	0.21098299
H	4.42822981	2.54263496	-0.151095
H	5.94931602	0.61898398	-0.177039
N	3.09020495	4.34357691	0.81892502
O	3.5202589	5.36109018	0.273467
O	3.63144898	3.79054904	1.77874196

Table S1(57). Optimized parameter of S_0 state of **1_5-NO$_2$**.

Atoms	X	Y	Z
Ir	0.001823	-0.016403	-0.003846
C	-2.019614	-0.085314	-0.011299
C	-2.859849	-0.044075	-1.142054
C	-2.6614871	-0.118838	1.26640403
C	-4.2477779	0.010991	-1.031538
H	-2.4120591	-0.032623	-2.131027
C	-4.0656662	-0.017205	1.36963904
C	-4.8557892	0.045886	0.23026
H	-4.8609729	0.050367	-1.929105
H	-5.9348102	0.128105	0.32264301
C	-1.766752	-0.146984	2.43027306
C	-2.088769	-0.321841	3.79595399
C	0.465525	0.24269199	3.10240412
C	-1.159116	-0.099132	4.80671883
C	0.150433	0.20814399	4.4556241
H	1.48241901	0.40714499	2.76552892
N	-0.450098	0.075372	2.13592291
H	0.91396499	0.37670201	5.20541096
H	-1.4610111	-0.20499	5.84235096
H	-4.5565829	0.045049	2.33281398
N	-3.3838041	-0.848134	4.24563503
O	-3.9268839	-0.287052	5.19760609
O	-3.8162749	-1.854309	3.68552089
C	0.25542799	-0.338496	-1.984996
C	0.53781998	0.630597	-2.968919
C	0.21511699	-1.699677	-2.419445
C	0.80423498	0.28611401	-4.292799
H	0.561324	1.67854905	-2.6862249
C	0.52898198	-2.0436189	-3.751658
C	0.81962597	-1.058755	-4.6854539
H	1.06530797	-1.3344541	-5.7068338
C	-0.068699	-2.6952071	-1.379409
C	-0.354429	-4.070951	-1.526807
C	-0.067342	-3.037503	0.956258
C	-0.377826	-4.939158	-0.439501
C	-0.211537	-4.4153128	0.836748
H	-0.001736	-2.5637541	1.92860603
N	0.00489	-2.2162139	-0.10134
H	-0.234299	-5.0465751	1.71680105
H	1.02155805	1.06472194	-5.020668
H	-0.561058	-5.9949751	-0.602769
H	0.57985502	-3.078614	-4.0674772
N	-0.746081	-4.6649232	-2.8113649
O	-0.231425	-5.7403688	-3.12046
O	-1.606757	-4.0850029	-3.470995
C	0.14352	2.00133204	-0.04011
C	1.46608698	2.54570103	-0.016763
C	-0.920132	2.92525411	0.00436
C	1.67445803	3.93294096	0.138248
C	-0.704834	4.29784822	0.111048
H	-1.940482	2.55638099	-0.030988
C	0.59788501	4.80641079	0.20027199
H	-1.553952	4.9768281	0.147258
H	0.76919103	5.8716259	0.32494599
C	2.560426	1.56954801	-0.068009
C	3.94310188	1.79568505	-0.252865
C	3.06886601	-0.714337	0.25567701
C	4.88509703	0.78856599	-0.067767
C	4.44129801	-0.499414	0.208707
H	2.65958691	-1.7081651	0.39573601
H	5.13427782	-1.3212301	0.342931
N	2.17138791	0.27502501	0.133036
H	5.9389019	1.01580703	-0.180471
H	2.67211008	4.34225702	0.241064
N	4.47947216	3.06858802	-0.751499
O	5.476614	3.52349496	-0.189683
O	3.93966508	3.57092404	-1.735657

Table S1(58). Optimized parameter of T_1 state of **1_5-NO₂**.

Atoms	X	Y	Z
Ir	-0.092089	-0.020538	-0.010038
C	-2.055465	-0.067499	-0.032421
C	-2.8786869	-0.026242	-1.189599
C	-2.7029569	-0.066769	1.27509403
C	-4.2530699	0.112948	-1.092312
H	-2.4104199	-0.059205	-2.1666651
C	-4.0938802	0.113821	1.346138
C	-4.8528371	0.20661099	0.17969599
H	-4.8658071	0.172996	-1.9869469
H	-5.9273038	0.34724	0.257875
C	-1.823754	-0.157967	2.43502688
C	-2.1788681	-0.33694	3.82121491
C	0.405545	0.30875301	3.07604909
C	-1.237861	0.054781	4.80528307
C	0.049231	0.37782699	4.4365449
H	1.42182803	0.47664401	2.74160004
N	-0.506897	0.067251	2.13319206
H	0.79537201	0.64667702	5.17631912
H	-1.548221	0.03142	5.84191608
H	-4.59231	0.17111599	2.30213308
N	-3.338855	-0.988435	4.25375891
O	-3.672986	-0.88904	5.48256207
O	-4.0118561	-1.69074	3.42805195
C	0.190418	-0.335137	-2.00193
C	0.42397001	0.64292997	-2.982332
C	0.237234	-1.7002569	-2.413264
C	0.728827	0.30114099	-4.3003678
H	0.37891299	1.69266605	-2.7095289
C	0.59117901	-2.037339	-3.736217
C	0.83329397	-1.043172	-4.675199
H	1.10880196	-1.31366	-5.6900568
C	-0.004106	-2.7003729	-1.363768
C	-0.214091	-4.0900369	-1.496326
C	-0.034821	-3.013911	0.98536998
C	-0.220778	-4.9411311	-0.396023
C	-0.112379	-4.3962941	0.87878901
H	-0.012957	-2.5189619	1.94881296
N	0.02232	-2.2100339	-0.088236
H	-0.12801	-5.0186682	1.76496899
H	0.90644598	1.08361006	-5.0340648
H	-0.344664	-6.007247	-0.548177
H	0.71151203	-3.069272	-4.0414748
N	-0.537896	-4.7224412	-2.783237
O	0.047259	-5.7679739	-3.0630059
O	-1.414327	-4.1997991	-3.4682059
C	0.124274	1.99781406	0.010913
C	1.45741904	2.50206304	0.010681
C	-0.922372	2.9315989	0.115962
C	1.69744301	3.87810493	0.199175
C	-0.673002	4.29661894	0.25094
H	-1.9502029	2.58517599	0.09563
C	0.64118898	4.77198792	0.315148
H	-1.506421	4.99026203	0.32877201
H	0.840253	5.82905579	0.46238101
C	2.53125691	1.504601	-0.090376
C	3.909307	1.71062505	-0.316228
C	3.00349307	-0.792834	0.177564
C	4.83580923	0.68204701	-0.181818
C	4.37669897	-0.602116	0.086925
H	2.58271194	-1.781013	0.31822899
H	5.05753613	-1.4387651	0.185417
N	2.12536812	0.218224	0.103595
H	5.88982821	0.891101	-0.323804
H	2.70569706	4.26249313	0.28848299
N	4.45190001	2.98661709	-0.804002
O	5.47297907	3.41058707	-0.263984
O	3.887187	3.51745009	-1.758

Table S1(59). Optimized parameter of S_0 state of **1_*6*-NO$_2$**.

Atoms	X	Y	Z
Ir	-0.010514	-0.020148	0.052186
C	-2.0355229	-0.003069	0.208086
C	-2.9822919	-0.031068	-0.834112
C	-2.5552399	0.031315	1.53610694
C	-4.355824	-0.02609	-0.58543
H	-2.636827	-0.052616	-1.863291
C	-3.9416361	0.033401	1.78407097
C	-4.8433318	0.005203	0.72758502
H	-4.3226819	0.056807	2.80107999
C	-1.570262	0.072708	2.61840391
C	-1.87686	0.144812	3.9872849
C	0.72491699	0.08349	3.12761211
C	-0.837182	0.182951	4.8993082
H	-2.8988431	0.17479099	4.33805895
C	0.49539	0.15318701	4.49404621
H	1.73481405	0.058845	2.73652697
N	-0.265395	0.042005	2.21799803
H	1.30916297	0.185082	5.20515394
H	-5.0541768	-0.046861	-1.41929
H	-5.9121351	0.006498	0.92112499
N	-1.150141	0.25963601	6.337255
O	-0.205033	0.29483899	7.12373114
O	-2.334605	0.28378201	6.66804218
C	0.03432	-0.245614	-1.9656709
C	0.09045	0.76210701	-2.94783
C	0.014461	-1.59143	-2.4387929
C	0.124901	0.46493599	-4.3113098
H	0.10185	1.80312896	-2.6394529
C	0.051596	-1.8882411	-3.8150041
C	0.106614	-0.864486	-4.7525291
H	0.037789	-2.917984	-4.1609068
C	-0.055528	-2.6386659	-1.417985
C	-0.11922	-4.0174909	-1.678664
C	-0.133532	-3.0672901	0.89231902
C	-0.187982	-4.8926601	-0.60924
H	-0.119741	-4.4032869	-2.688309
C	-0.196909	-4.4409051	0.708655
H	-0.139048	-2.641211	1.88830805
N	-0.062758	-2.1929109	-0.127606
H	-0.252925	-5.123198	1.54546297
H	0.166263	1.27356899	-5.0378189
H	0.135628	-1.096029	-5.813355
N	-0.256874	-6.3406968	-0.873637
O	-0.319272	-7.0936179	0.09708
O	-0.247976	-6.7127729	-2.0460081
C	0.211239	1.99865699	0.037255
C	1.55638301	2.47424698	0.042105
C	-0.799095	2.97989988	0.031771
C	1.84991705	3.85170698	0.045752
C	-0.505044	4.34446192	0.032103
H	-1.839639	2.66977596	0.02193
C	0.823681	4.788064	0.040288
H	2.879107	4.19944286	0.053374
C	2.60679793	1.45438397	0.034291
C	3.98650408	1.715958	-0.001764
C	3.04081011	-0.856202	0.047558
C	4.86477709	0.64687097	-0.009202
H	4.37082911	2.72582793	-0.028264
C	4.41538906	-0.671611	0.015971
H	2.61647201	-1.852783	0.064867
H	5.1001668	-1.508227	0.008116
N	2.16339397	0.163441	0.058903
H	-1.3155921	5.06999493	0.026135
H	1.05273402	5.849823	0.043486
N	6.31378698	0.912139	-0.048198
O	7.06947279	-0.05841	-0.055076
O	6.68387318	2.084939	-0.071301

Table S1(60). Optimized parameter of T_1 state of **1**_*6*-**NO_2**.

Atoms	X	Y	Z
Ir	-0.077085	0.005244	0.047523
C	-2.0498431	0.015594	0.18946201
C	-2.97734	-0.051358	-0.885149
C	-2.5847011	0.089214	1.53271306
C	-4.343029	-0.013068	-0.661726
H	-2.603226	-0.108864	-1.901062
C	-3.971354	0.120051	1.73787606
C	-4.8423948	0.073744	0.654594
H	-4.3790689	0.17591999	2.74154592
C	-1.613445	0.102847	2.62596893
C	-1.936312	0.17498	3.97196293
C	0.682827	0.073845	3.13948202
C	-0.909148	0.195328	4.94013882
H	-2.962683	0.216143	4.30897903
C	0.43852201	0.14684799	4.49276114
H	1.69878495	0.036582	2.76253796
N	-0.298245	0.038463	2.20479012
H	1.24961805	0.168917	5.20738792
H	-5.035203	-0.04652	-1.498196
H	-5.9144521	0.100236	0.825589
N	-1.21187	0.26403001	6.29590797
O	-0.25327	0.281993	7.13750601
O	-2.4350431	0.30661401	6.65643406
C	0.008497	-0.241884	-1.977582
C	0.070161	0.75911897	-2.959774
C	0.017703	-1.5943461	-2.4252291
C	0.12659	0.445876	-4.3199568
H	0.06358	1.80274904	-2.6603839
C	0.078932	-1.905259	-3.7969821
C	0.130924	-0.888643	-4.7430239
H	0.086987	-2.937588	-4.1335421
C	-0.028853	-2.631531	-1.391301
C	-0.048015	-4.0155711	-1.635735
C	-0.09788	-3.03911	0.93136299
C	-0.090711	-4.8762631	-0.554908
H	-0.03244	-4.4144468	-2.640013
C	-0.115663	-4.4149299	0.75948799
H	-0.118831	-2.5947721	1.91915905
N	-0.054815	-2.1810401	-0.103414
H	-0.150956	-5.091969	1.60160398
H	0.165766	1.24553502	-5.0555949
H	0.17588	-1.1316119	-5.8004079
N	-0.112127	-6.333518	-0.804579
O	-0.144547	-7.0738082	0.175202
O	-0.096002	-6.7137709	-1.972652
C	0.202905	2.01269507	0.101978
C	1.55390298	2.46058202	0.066671
C	-0.799763	2.99569798	0.187243
C	1.86149096	3.83240509	0.107478
C	-0.486494	4.35518408	0.21853399
H	-1.8427661	2.69764495	0.20938499
C	0.84661001	4.77921486	0.179763
H	2.89339399	4.16877794	0.086272
C	2.59046888	1.42825794	0.005112
C	3.97076392	1.67803395	-0.065713
C	2.99619293	-0.885953	-0.049967
C	4.83338404	0.59935498	-0.126734
H	4.36596918	2.68381	-0.076228
C	4.37110806	-0.713723	-0.120021
H	2.56434989	-1.878767	-0.040295
H	5.04589891	-1.557091	-0.167346
N	2.13554502	0.14444201	0.014571
H	-1.28692	5.08877611	0.27193001
H	1.09026897	5.83688498	0.207642
N	6.28712988	0.85034502	-0.203448
O	7.02806997	-0.128567	-0.253509
O	6.6661191	2.01914907	-0.212741

Table S1(61). Optimized parameter of S_0 state of **1_7-NO$_2$**.

Atoms	X	Y	Z
Ir	0.001333	-0.025605	0.059028
C	-2.016228	-0.00895	0.23154099
C	-2.969192	-0.039209	-0.806016
C	-2.531687	0.025573	1.56508899
C	-4.3403711	-0.037156	-0.550826
H	-2.629025	-0.060074	-1.836856
C	-3.9204609	0.024587	1.81708097
C	-4.8242798	-0.006591	0.76536202
H	-4.2979388	0.047301	2.83498406
H	-5.8924608	-0.007981	0.96149498
C	-1.552236	0.070788	2.6437161
C	-1.8522201	0.147192	4.01922703
C	0.74309802	0.087221	3.13397598
C	-0.838281	0.192773	4.95762682
H	-2.8826771	0.17507	4.34969997
C	0.48124099	0.16176701	4.4977088
H	1.75951302	0.06488	2.76439095
H	-1.0459009	0.253288	6.0186491
N	-0.243724	0.040425	2.23521805
H	-5.042316	-0.059641	-1.381542
N	1.59073496	0.20930199	5.43373585
O	1.32552803	0.27296901	6.63653708
O	2.73765206	0.183943	4.97771597
C	0.042565	-0.263882	-1.951273
C	0.099175	0.74089098	-2.9375889
C	0.021861	-1.6141911	-2.4223781
C	0.134012	0.43987	-4.2990289
H	0.110468	1.78282905	-2.632637
C	0.059625	-1.912493	-3.801394
C	0.115711	-0.891789	-4.7389679
H	0.045906	-2.9423499	-4.1456199
H	0.145404	-1.123897	-5.7995009
C	-0.049603	-2.6598661	-1.409135
C	-0.117616	-4.044569	-1.665818
C	-0.128855	-3.074297	0.89977598
C	-0.190808	-4.9491859	-0.623178
H	-0.11722	-4.408371	-2.685277
C	-0.196232	-4.445941	0.68083698
H	-0.134731	-2.6717329	1.90388095
H	-0.245371	-6.016561	-0.797197
N	-0.054887	-2.208494	-0.114452
H	0.17569999	1.24659705	-5.0275512
N	-0.274298	-5.3449459	1.81893504
O	-0.281952	-4.8513022	2.95040298
O	-0.329115	-6.5559659	1.59195197
C	0.23463	1.98566401	0.044056
C	1.58392596	2.46024394	0.047594
C	-0.773511	2.97034502	0.04184
C	1.87792695	3.8407321	0.054223
C	-0.476623	4.33310223	0.045114
H	-1.814727	2.66284108	0.032174
C	0.85401797	4.77642298	0.052991
H	2.90700293	4.18748617	0.06145
C	2.63354611	1.44872606	0.034
C	4.01897383	1.70741606	-0.007758
C	3.05525708	-0.860197	0.037357
C	4.92755318	0.66582799	-0.025095
H	4.38036919	2.72746611	-0.031516
C	4.42768002	-0.639278	-0.001756
H	2.65529704	-1.865238	0.052378
H	5.99554014	0.84139401	-0.058617
N	2.18553495	0.15312099	0.057731
H	1.08281898	5.83807421	0.05883
H	-1.285828	5.06006289	0.0417
N	5.33102798	-1.776361	-0.021272
O	6.5423851	-1.54746	-0.057033
O	4.84045982	-2.9090409	-0.001392

Table S1(62). Optimized parameter of T_1 state of **1**_*7*-**NO**$_2$.

Atoms	X	Y	Z
Ir	-0.073019	-0.015109	0.059931
C	-2.039145	0.013484	0.20033801
C	-2.973572	-0.043301	-0.868114
C	-2.5632069	0.092243	1.55829597
C	-4.3402009	-0.00326	-0.641483
H	-2.6027789	-0.100036	-1.885465
C	-3.950933	0.128197	1.76378202
C	-4.825727	0.083907	0.68002701
H	-4.360136	0.187305	2.76676989
H	-5.8967009	0.114136	0.85935301
C	-1.596659	0.109282	2.63726902
C	-1.891572	0.177457	4.0194602
C	0.70675099	0.075188	3.11947989
C	-0.883201	0.19821399	4.95696211
H	-2.924058	0.212495	4.34828377
C	0.46316099	0.149451	4.51038218
H	1.72327304	0.035757	2.75314903
H	-1.088095	0.250788	6.01805305
N	-0.278352	0.051994	2.23393607
H	-5.0378599	-0.035385	-1.472852
N	1.53321505	0.17203601	5.40313816
O	1.28384697	0.238894	6.64862299
O	2.72093797	0.12508699	4.94346809
C	-0.000141	-0.26282	-1.9609391
C	0.036734	0.73780501	-2.944762
C	0.026902	-1.617139	-2.409801
C	0.092007	0.42464101	-4.3046451
H	0.013307	1.78153503	-2.647064
C	0.08539	-1.926531	-3.784472
C	0.116746	-0.910157	-4.7294021
H	0.107158	-2.958405	-4.1208701
H	0.16075701	-1.151052	-5.7872062
C	-0.00551	-2.654614	-1.383311
C	-0.015889	-4.043344	-1.622345
C	-0.063901	-3.0472779	0.93936199
C	-0.052333	-4.9351058	-0.566502
H	0.000703	-4.4203472	-2.636554
C	-0.077692	-4.4197292	0.733073
H	-0.080903	-2.627218	1.936391
H	-0.063075	-6.0059471	-0.727394
N	-0.028425	-2.196306	-0.091691
H	0.114709	1.22486401	-5.0403481
N	-0.120809	-5.3119741	1.88205302
O	-0.154933	-4.805274	3.00580192
O	-0.120203	-6.5246582	1.66391397
C	0.21899199	1.99569905	0.087221
C	1.57234204	2.440938	0.07082
C	-0.781534	2.98219609	0.13785701
C	1.88114202	3.8158071	0.101871
C	-0.465719	4.34142208	0.16289701
H	-1.825608	2.68715692	0.144787
C	0.86871499	4.76434088	0.145789
H	2.91375089	4.14966917	0.095009
C	2.61015701	1.41527605	0.026447
C	3.99570394	1.66158605	-0.031831
C	3.00487709	-0.897172	-0.015665
C	4.89142609	0.609321	-0.081761
H	4.36797619	2.6775651	-0.042014
C	4.37730598	-0.689229	-0.073494
H	2.59724903	-1.898743	-0.009172
H	5.9607172	0.77352101	-0.128597
N	2.14986706	0.127648	0.036703
H	1.11298001	5.82193518	0.168486
H	-1.266151	5.07633591	0.195746
N	5.26877117	-1.838161	-0.127806
O	6.48036385	-1.6201021	-0.177766
O	4.76321077	-2.9632011	-0.122269

Table S1(63). Optimized parameter of S_0 state of **1**_*2*-**CN**.

Atoms	X	Y	Z
Ir	-0.024677	-0.003472	0.028297
C	-2.0446451	0.023509	0.207197
C	-2.9969161	-0.001182	-0.82508
C	-2.5528181	0.063661	1.54053903
C	-4.3774729	0.011355	-0.559196
H	-2.6703551	-0.026984	-1.859169
C	-3.9345939	0.073196	1.80219901
C	-4.8542872	0.047932	0.764054
H	-4.3066111	0.100079	2.82143307
H	-5.920239	0.054914	0.96431601
C	-1.5533921	0.097113	2.62102389
C	-1.841719	0.163296	3.99186206
C	0.74015099	0.087641	3.09801602
C	-0.806388	0.189658	4.91971779
H	-2.870043	0.19659699	4.33126688
C	0.51412898	0.150089	4.46795082
H	1.74519503	0.057897	2.69296193
H	-1.0273221	0.241624	5.98131609
N	-0.258298	0.060111	2.19828701
H	1.35286295	0.169498	5.15489388
C	-5.308435	-0.01473	-1.648492
N	-6.0619378	-0.037047	-2.536459
C	0.008569	-0.246741	-1.984737
C	0.062993	0.75367302	-2.9692841
C	-0.02036	-1.596091	-2.4500229
C	0.088588	0.442911	-4.340282
H	0.08173	1.79807699	-2.6768379
C	0.007351	-1.90257	-3.822283
C	0.061279	-0.89512	-4.7743468
H	-0.01271	-2.933213	-4.1621661
H	0.083158	-1.130338	-5.8329258
C	-0.083825	-2.643743	-1.417416
C	-0.144419	-4.0230598	-1.663516
C	-0.140453	-3.046809	0.88961798
C	-0.201907	-4.917201	-0.600122
H	-0.149305	-4.3947482	-2.6810451
C	-0.199061	-4.423204	0.70585501
H	-0.139101	-2.6097009	1.88166201
H	-0.249659	-5.9852958	-0.788211
N	-0.082747	-2.179744	-0.136025
H	-0.243644	-5.0827618	1.56535399
C	0.14457101	1.50103998	-5.305244
N	0.191513	2.36361289	-6.086597
C	0.21824799	2.00960493	0.005747
C	1.56787097	2.47512102	0.002217
C	-0.783317	2.99461293	-0.000073
C	1.873353	3.84792995	-0.00157
C	-0.473461	4.36601305	-0.006671
H	-1.827855	2.70201612	-0.003943
C	0.86478102	4.80029106	-0.006465
H	2.90414906	4.18799114	-0.000297
C	2.61702108	1.44215906	-0.000244
C	3.99748111	1.68742895	-0.031811
C	3.02165103	-0.865102	0.029489
C	4.89291191	0.62358099	-0.030107
H	4.36915207	2.70455909	-0.060572
C	4.3991251	-0.682069	0.00259
H	2.58479905	-1.857007	0.051774
H	5.96188688	0.81103402	-0.055337
H	5.05971384	-1.541931	0.004382
N	2.15331411	0.16105001	0.029552
H	1.09922397	5.8592658	-0.008981
C	-1.5328341	5.3312192	-0.012464
N	-2.3965581	6.11270523	-0.01614

Table S1(64). Optimized parameter of T_1 state of **1**_*2*-**CN**.

Atoms	X	Y	Z
Ir	-0.071808	-0.008136	0.020118
C	-2.0454099	0.042086	0.16210701
C	-2.986172	0.001738	-0.867389
C	-2.546113	0.128317	1.56802905
C	-4.377336	0.052136	-0.621243
H	-2.646261	-0.058453	-1.895433
C	-3.963454	0.19081201	1.79456401
C	-4.841784	0.15401401	0.75174499
H	-4.3528962	0.266976	2.80441189
H	-5.9106598	0.200874	0.93793499
C	-1.584735	0.123056	2.60129595
C	-1.862141	0.18695299	4.00120592
C	0.72828197	0.061948	3.08520293
C	-0.832685	0.19147301	4.91542578
H	-2.890486	0.231461	4.340693
C	0.50270897	0.131244	4.45824099
H	1.73744702	0.010895	2.6897161
H	-1.046599	0.241219	5.97877121
N	-0.256398	0.047766	2.18044996
H	1.34154701	0.13652501	5.14456081
C	-5.3082681	0.013094	-1.678974
N	-6.0852051	-0.021305	-2.5571859
C	-0.017401	-0.260244	-2.0009201
C	0.025791	0.73590702	-2.9859879
C	-0.011598	-1.6129	-2.450104
C	0.070374	0.415773	-4.3548532
H	0.018353	1.78205299	-2.699199
C	0.03394	-1.928727	-3.819684
C	0.074183	-0.92566	-4.7772908
H	0.038285	-2.961328	-4.15341
H	0.108982	-1.168246	-5.8337879
C	-0.051616	-2.655165	-1.409393
C	-0.072357	-4.0371099	-1.645005
C	-0.099168	-3.0452991	0.90574801
C	-0.10667	-4.9235072	-0.573971
H	-0.064116	-4.4171629	-2.659159
C	-0.119542	-4.4230261	0.72999102
H	-0.110521	-2.596334	1.89239204
H	-0.123921	-5.9936791	-0.754886
N	-0.066366	-2.188159	-0.129083
H	-0.147683	-5.0792532	1.59247506
C	0.112184	1.46717298	-5.327877
N	0.147708	2.32409596	-6.1157332
C	0.200662	2.00569606	0.025021
C	1.55121005	2.45909691	0.021995
C	-0.801066	2.98767591	0.045279
C	1.85839999	3.83121896	0.043488
C	-0.486941	4.35776711	0.063516
H	-1.845191	2.69478703	0.036493
C	0.852476	4.78582716	0.063466
H	2.88970494	4.16831017	0.046073
C	2.59938693	1.42512703	-0.004145
C	3.9788661	1.67192495	-0.047718
C	3.00298905	-0.882169	-0.018296
C	4.87316513	0.607427	-0.074285
H	4.35084581	2.68898392	-0.064415
C	4.37999201	-0.698501	-0.058492
H	2.56753802	-1.874634	-0.006455
H	5.94173384	0.79514903	-0.108592
H	5.04034185	-1.558128	-0.078518
N	2.13746905	0.14507399	0.010958
H	1.09103298	5.84363699	0.079185
C	-1.543568	5.32575703	0.082234
N	-2.404053	6.11027813	0.098135

Table S1(65). Optimized parameter of S_0 state of **1**_*3*-**CN**.

Atoms	X	Y	Z
Ir	-0.02644	-0.005333	0.031865
C	-2.0387461	0.029312	0.21591599
C	-3.0006919	0.009062	-0.817232
C	-2.5400889	0.067697	1.55460405
C	-4.366776	0.023392	-0.564233
H	-2.6690071	-0.014741	-1.85026
C	-3.9130549	0.078691	1.81972396
C	-4.8353691	0.05722	0.76430798
H	-4.2894058	0.103053	2.8367331
C	-1.5384671	0.09702	2.63412809
C	-1.821758	0.160422	4.00608921
C	0.75709897	0.082091	3.10293198
C	-0.782684	0.182218	4.92952919
H	-2.8485031	0.194809	4.35045624
C	0.53658998	0.141404	4.47328186
H	1.76021302	0.051393	2.6932919
H	-0.999735	0.231681	5.99205685
N	-0.245923	0.058596	2.20748997
H	1.37761903	0.157417	5.15742207
H	-5.0793281	0.008197	-1.383803
C	-6.2384172	0.06844	1.03772104
N	-7.3827081	0.07728	1.25811303
C	-0.000377	-0.254041	-1.973469
C	0.04856	0.74693602	-2.967881
C	-0.026345	-1.6085761	-2.4315679
C	0.072307	0.44940001	-4.3248382
H	0.063486	1.79052806	-2.6704531
C	0.00063	-1.91837	-3.794946
C	0.049716	-0.89371	-4.7503619
H	-0.015284	-2.947026	-4.1390529
C	-0.086203	-2.655026	-1.396508
C	-0.143527	-4.0353708	-1.637188
C	-0.139968	-3.0493169	0.91252899
C	-0.197434	-4.9247389	-0.5699
H	-0.148493	-4.4121628	-2.652936
C	-0.194945	-4.4260182	0.73462999
H	-0.138848	-2.6073141	1.90237498
H	-0.242267	-5.9936991	-0.753792
N	-0.085238	-2.1869741	-0.11783
H	-0.236977	-5.0825448	1.59651005
H	0.108493	1.24525106	-5.063035
C	0.077313	-1.212734	-6.1435318
N	0.100229	-1.4702851	-7.2798381
C	0.222657	1.99963903	0.00166
C	1.57750201	2.45772696	0.000571
C	-0.779087	2.99457312	-0.009456
C	1.88663101	3.82155204	-0.004451
C	-0.482077	4.35179806	-0.0181
H	-1.822752	2.69706011	-0.016087
C	0.86122102	4.77734709	-0.014099
H	2.91538906	4.16574621	-0.000299
C	2.62512493	1.42218399	0.00246
C	4.00657701	1.66177905	-0.026154
C	3.0203371	-0.887051	0.037598
C	4.89687395	0.593934	-0.019891
H	4.38360214	2.677001	-0.055917
C	4.39805698	-0.710119	0.013974
H	2.57840204	-1.876654	0.060856
H	5.96667719	0.77699101	-0.042543
H	5.05533791	-1.572436	0.019183
N	2.15706611	0.143932	0.033532
H	-1.278468	5.09023905	-0.02752
C	1.179654	6.17090321	-0.018803
N	1.43673801	7.3075428	-0.022279

Table S1(66). Optimized parameter of T_1 state of **1**_*3*-**CN**.

Atoms	X	Y	Z
Ir	-0.053623	-0.001206	0.022119
C	-2.0345769	0.039615	0.17587
C	-2.995203	0.002467	-0.846281
C	-2.5243981	0.106979	1.58432901
C	-4.3648982	0.044291	-0.599003
H	-2.6600201	-0.047415	-1.877476
C	-3.9375789	0.162222	1.82347095
C	-4.8201208	0.129691	0.76313901
H	-4.3263612	0.229546	2.83299088
C	-1.566192	0.095437	2.61001396
C	-1.833101	0.137723	4.02057505
C	0.759139	0.067532	3.0778749
C	-0.804694	0.151288	4.92623091
H	-2.8609109	0.155975	4.36576891
C	0.53815401	0.122654	4.45310402
H	1.76971602	0.03906	2.68261409
H	-1.009444	0.183101	5.9917798
N	-0.223003	0.039555	2.1731441
H	1.38127697	0.139645	5.13391018
H	-5.0891089	0.02274	-1.405334
C	-6.2267008	0.183239	1.025895
N	-7.3736668	0.225439	1.22567701
C	-0.010797	-0.269784	-1.9917769
C	0.044638	0.72181797	-2.9916029
C	-0.024778	-1.6288281	-2.4302671
C	0.081062	0.409908	-4.3458629
H	0.052378	1.76838601	-2.7038441
C	0.013865	-1.95311	-3.7904561
C	0.065805	-0.937566	-4.755157
H	0.005012	-2.984946	-4.124712
C	-0.075353	-2.6669891	-1.384316
C	-0.116171	-4.0499182	-1.6124001
C	-0.120428	-3.042907	0.93205601
C	-0.159471	-4.9292488	-0.536102
H	-0.116459	-4.4368691	-2.624151
C	-0.160981	-4.4210348	0.76518202
H	-0.121343	-2.5867541	1.91561306
H	-0.192295	-6.0001478	-0.710801
N	-0.078862	-2.1925881	-0.108127
H	-0.19511	-5.0719862	1.63149703
H	0.120121	1.19746006	-5.0925102
C	0.104508	-1.271627	-6.1453371
N	0.136718	-1.541168	-7.2783132
C	0.207937	2.00239706	0.017719
C	1.561602	2.45748901	0.018918
C	-0.798874	2.99022508	0.031422
C	1.86551297	3.82254004	0.040961
C	-0.504507	4.34798002	0.048951
H	-1.840356	2.68615794	0.017738
C	0.83759201	4.77481604	0.055423
H	2.89312601	4.169034	0.048276
C	2.61310291	1.42584503	-0.004484
C	3.9927671	1.672557	-0.044972
C	3.01657891	-0.88187	-0.023999
C	4.88634109	0.60775799	-0.072896
H	4.3660121	2.68940091	-0.0583
C	4.39315701	-0.698719	-0.0622
H	2.57905602	-1.873565	-0.015324
H	5.95504618	0.79539502	-0.104657
H	5.05375719	-1.558113	-0.084177
N	2.15160203	0.146365	0.007529
H	-1.302787	5.08399582	0.05789
C	1.15339506	6.16956186	0.076452
N	1.40790999	7.30628681	0.093988

Table S1(67). Optimized parameter of S_0 state of **1**_*4*-**CN**.

Atoms	X	Y	Z
Ir	0.003011	-0.034926	0.064526
C	-2.016005	-0.004585	0.26598001
C	-2.950172	-0.051397	-0.789082
C	-2.5258441	0.048596	1.59886897
C	-4.3263202	-0.052262	-0.574329
H	-2.5842421	-0.08576	-1.810236
C	-3.9354539	0.033779	1.81026304
C	-4.8248501	-0.013984	0.72443599
H	-5.8930702	-0.024541	0.913149
C	-1.509005	0.120076	2.671278
C	-1.742751	0.240858	4.05051279
C	0.80559099	0.127084	3.08383703
C	-0.676826	0.29761299	4.94312
H	-2.750093	0.294792	4.4344039
C	0.62813598	0.236489	4.45717716
H	1.79578495	0.084243	2.64532495
H	-0.869392	0.39099199	6.00742388
N	-0.220917	0.070066	2.21891308
H	1.48896301	0.27737799	5.11516619
H	-5.0144138	-0.087063	-1.414694
C	-4.5770211	0.050266	3.09166193
N	-5.1727662	0.0563	4.093297
C	0.034382	-0.30573	-1.946148
C	0.106805	0.71619701	-2.9148369
C	-0.004702	-1.655649	-2.4105871
C	0.14542601	0.45387799	-4.282208
H	0.12970801	1.74952602	-2.5838759
C	0.048728	-1.915776	-3.8111479
C	0.121021	-0.861429	-4.736095
H	0.160603	-1.087074	-5.7964611
C	-0.104835	-2.6926539	-1.359702
C	-0.217739	-4.0792451	-1.549469
C	-0.177768	-3.0257659	0.96666199
C	-0.304073	-4.934988	-0.455618
H	-0.242752	-4.4972181	-2.544261
C	-0.280554	-4.4045959	0.83319199
H	-0.163676	-2.5533061	1.94200099
H	-0.391023	-6.005425	-0.614137
N	-0.092462	-2.196548	-0.086878
H	-0.345178	-5.032958	1.71444702
H	0.19860999	1.26991498	-4.9978581
C	0.048956	-3.218473	-4.4084592
N	0.058301	-4.2397542	-4.96982
C	0.27219099	1.97609699	0.038778
C	1.62238896	2.44109511	0.025643
C	-0.75177	2.9453671	0.0526
C	1.88041496	3.8428371	0.050653
C	-0.491177	4.31358194	0.061356
H	-1.785205	2.61393499	0.053829
C	0.82412302	4.76820803	0.065789
C	2.66238093	1.389498	-0.017337
C	4.05060387	1.57790697	-0.110822
C	2.99953389	-0.93725	-0.003336
C	4.90917921	0.48332801	-0.14204
H	4.46760988	2.5720191	-0.164355
C	4.38002014	-0.804847	-0.082843
H	2.52810502	-1.912447	0.034758
H	5.9808588	0.64066899	-0.214661
H	5.01062822	-1.686621	-0.103983
N	2.1675179	0.117017	0.028051
H	1.04807103	5.82949305	0.08519
H	-1.3086931	5.02946901	0.07003
C	3.18252897	4.44071722	0.079821
N	4.20323086	5.00237513	0.110307

Table S1(68). Optimized parameter of T_1 state of **1_*4*-CN**.

Atoms	X	Y	Z
Ir	-0.048892	-0.055857	0.051462
C	-2.0185089	-0.042542	0.232302
C	-2.953965	-0.218132	-0.793958
C	-2.5096641	0.164919	1.62577403
C	-4.3369231	-0.142065	-0.564152
H	-2.5999801	-0.390898	-1.804346
C	-3.947536	0.355955	1.80941296
C	-4.8093019	0.164867	0.71601498
H	-5.8777418	0.27624401	0.87964898
C	-1.535514	0.125275	2.64684892
C	-1.766343	0.165326	4.06102419
C	0.797252	0.016728	3.07722497
C	-0.712875	0.14415	4.94540501
H	-2.7795849	0.203155	4.43539906
C	0.61215401	0.081767	4.45616007
H	1.79446101	-0.048336	2.65404391
H	-0.901754	0.170682	6.01446104
N	-0.209376	0.01911	2.19871998
H	1.46922302	0.074226	5.11942482
H	-5.0420432	-0.276866	-1.3782491
C	-4.5589209	0.75792599	3.02218604
N	-5.1139832	1.09723699	3.99665999
C	-0.001325	-0.32709	-1.96872
C	0.038302	0.698322	-2.930975
C	0.012665	-1.677614	-2.424073
C	0.088231	0.43506601	-4.2983031
H	0.023416	1.73164201	-2.5992191
C	0.073755	-1.937945	-3.824137
C	0.108564	-0.881253	-4.7485008
H	0.153648	-1.10642	-5.8086591
C	-0.038625	-2.7162869	-1.369904
C	-0.08034	-4.106895	-1.5555821
C	-0.096496	-3.0489211	0.96293598
C	-0.127385	-4.9617062	-0.458346
H	-0.079149	-4.529141	-2.5487299
C	-0.133988	-4.4304071	0.83072001
H	-0.105273	-2.568841	1.93452704
H	-0.160319	-6.035367	-0.614726
N	-0.050427	-2.2233291	-0.095593
H	-0.17199	-5.0608831	1.71174395
H	0.112859	1.25059402	-5.0156231
C	0.11404	-3.2408471	-4.4195342
N	0.15298	-4.2628241	-4.9778509
C	0.230051	1.95695996	0.071361
C	1.57576299	2.42395806	0.055784
C	-0.805668	2.90992498	0.122496
C	1.82133901	3.82684708	0.110857
C	-0.554237	4.27931404	0.16052601
H	-1.835215	2.5681839	0.122846
C	0.75737298	4.74189091	0.159474
C	2.62429595	1.38220096	-0.016485
C	4.00934696	1.58783996	-0.113443
C	2.98391294	-0.940515	-0.067357
C	4.87770605	0.50267398	-0.17997
H	4.41646624	2.58683491	-0.14196
C	4.36216784	-0.791819	-0.153057
H	2.5249331	-1.921821	-0.050226
H	5.94717216	0.67266899	-0.254733
H	5.00114584	-1.666232	-0.201649
N	2.14407396	0.105046	0.000182
H	0.97343999	5.80408287	0.199228
H	-1.377148	4.98755121	0.195005
C	3.1186111	4.43501186	0.137844
N	4.13381386	5.00606489	0.167522

Table S1(69). Optimized parameter of S_0 state of **1**_*5*-**CN**.

Atoms	X	Y	Z
Ir	-0.026205	-0.008313	0.033561
C	-2.043555	-0.033051	0.18498001
C	-2.9626291	-0.108925	-0.880212
C	-2.5913429	0.004518	1.50787306
C	-4.3402319	-0.154837	-0.671926
H	-2.586185	-0.131326	-1.898355
C	-3.987385	-0.056236	1.70771801
C	-4.8580418	-0.134014	0.62840801
H	-4.4081931	-0.050333	2.70391202
H	-5.929718	-0.181048	0.79928899
C	-1.619058	0.098801	2.60559201
C	-1.873019	0.24336	3.99942207
C	0.69533598	0.10613	3.0819869
C	-0.806524	0.29716799	4.91197109
C	0.500117	0.21989	4.45258808
H	1.69254804	0.060125	2.65996599
H	-1.019806	0.40522099	5.96996498
N	-0.314407	0.048259	2.20088601
H	1.34531999	0.25830701	5.12931681
H	-5.0148192	-0.211915	-1.523537
C	-3.1752191	0.369091	4.58078384
N	-4.1868339	0.48672399	5.14495182
C	0.057993	-0.223734	-1.9759049
C	0.15121301	0.81087399	-2.9275801
C	0.040003	-1.564371	-2.480643
C	0.229876	0.55792397	-4.2962542
H	0.159898	1.84094703	-2.5846269
C	0.13154601	-1.809166	-3.8677859
C	0.22485	-0.758609	-4.771451
H	0.13564201	-2.8183401	-4.2563472
H	0.29525	-0.964353	-5.8356948
C	-0.073921	-2.630609	-1.4755861
C	-0.190298	-4.03439	-1.685836
C	-0.162601	-3.0274861	0.852539
C	-0.276168	-4.9100428	-0.590778
C	-0.256734	-4.4049878	0.70109302
H	-0.157849	-2.569741	1.83490396
H	-0.362312	-5.9761362	-0.771009
N	-0.074157	-2.18153	-0.184578
H	-0.3219	-5.0524902	1.56727803
H	0.299088	1.38731599	-4.9968619
C	-0.249492	-4.663805	-2.9702931
N	-0.311529	-5.2661638	-3.9646781
C	0.19247	2.00294805	0.067756
C	1.53443897	2.50445604	0.0693
C	-0.841287	2.959198	0.113034
C	1.78095901	3.892874	0.132551
C	-0.586489	4.32886982	0.163344
H	-1.8723741	2.61918211	0.106834
C	0.73107702	4.8009572	0.177808
H	2.79091311	4.27895308	0.15311299
C	2.60055208	1.49483204	0.00986
C	4.00680494	1.69960904	-0.084049
C	2.99503708	-0.835091	0.010166
C	4.88232088	0.60143298	-0.111416
C	4.37466478	-0.688386	-0.056828
H	2.53606606	-1.816456	0.038267
H	5.95034504	0.77736902	-0.180774
H	5.02192497	-1.556991	-0.076039
N	2.14898396	0.20514999	0.042477
H	0.93804902	5.86615992	0.226961
H	-1.4153611	5.03277206	0.195528
C	4.63881683	2.98066711	-0.178712
N	5.24329901	3.97178411	-0.266914

Table S1(70). Optimized parameter of T_1 state of **1**_*5*-**CN**.

Atoms	X	Y	Z
Ir	-0.056428	0.003734	-0.008042
C	-2.0730181	-0.02994	0.178489
C	-2.9902799	-0.127164	-0.884142
C	-2.6062329	0.014031	1.50389397
C	-4.366281	-0.184794	-0.667119
H	-2.616564	-0.149937	-1.9028601
C	-3.999804	-0.059702	1.70939302
C	-4.8752422	-0.156066	0.63510901
H	-4.4150252	-0.050754	2.70750189
H	-5.9451008	-0.211471	0.81306899
C	-1.6296639	0.120252	2.59805393
C	-1.881295	0.27199	3.9910481
C	0.68601	0.153916	3.06371999
C	-0.811002	0.34386301	4.89742613
C	0.49473399	0.27757701	4.43358421
H	1.68227005	0.113418	2.63967299
H	-1.02052	0.4571	5.95555878
N	-0.328771	0.076393	2.19056392
H	1.34196496	0.32991999	5.10662508
H	-5.0435829	-0.256841	-1.51481
C	-3.1828041	0.385934	4.57650995
N	-4.1937928	0.49475199	5.14313316
C	0.007719	-0.172574	-1.973931
C	0.080162	0.87200302	-2.9205611
C	-0.026843	-1.57076	-2.471591
C	0.086015	0.63315898	-4.289578
H	0.109928	1.89534497	-2.560714
C	-0.037927	-1.778307	-3.8801501
C	0.017414	-0.705594	-4.7533441
H	-0.092444	-2.7777929	-4.2893958
H	0.007027	-0.898865	-5.8234038
C	-0.040667	-2.6056581	-1.498587
C	-0.008557	-4.057724	-1.714439
C	-0.215795	-2.99967	0.83978701
C	-0.140252	-4.9121499	-0.609681
C	-0.263333	-4.4041152	0.67614901
H	-0.270303	-2.556798	1.82843697
H	-0.130662	-5.9853201	-0.775412
N	-0.100412	-2.1605711	-0.173337
H	-0.367191	-5.0509229	1.53863597
H	0.12917501	1.45378494	-4.9991169
C	0.187417	-4.6851521	-2.9670081
N	0.34733799	-5.2707119	-3.9693601
C	0.203769	2.01956105	0.04317
C	1.55694699	2.48346901	0.057548
C	-0.809885	2.99180102	0.089605
C	1.83437204	3.8652699	0.127106
C	-0.524054	4.35614014	0.147701
H	-1.8479151	2.67399812	0.074871
C	0.80349302	4.79584408	0.169624
H	2.851969	4.22964716	0.15530901
C	2.60230994	1.448825	0.017553
C	4.01357603	1.622105	-0.059012
C	2.94741797	-0.89485	0.051572
C	4.86297178	0.50325298	-0.065831
C	4.32966423	-0.776368	-0.002847
H	2.461339	-1.862833	0.08963
H	5.93527412	0.65521598	-0.124989
H	4.96041107	-1.657022	-0.006071
N	2.1292181	0.167863	0.057883
H	1.03472698	5.85568714	0.222831
H	-1.336287	5.07871294	0.178468
C	4.67404699	2.8884511	-0.156306
N	5.29901505	3.86639905	-0.246276

Table S1(71). Optimized parameter of S_0 state of **1**_*6*-**CN**.

Atoms	X	Y	Z
Ir	-0.009182	-0.021684	0.050592
C	-2.0330861	-0.003745	0.212256
C	-2.9825399	-0.034035	-0.827971
C	-2.5505281	0.033099	1.54152298
C	-4.3557181	-0.028917	-0.577558
H	-2.638562	-0.05759	-1.857703
C	-3.9366701	0.034917	1.79098499
C	-4.840694	0.0045	0.73620999
H	-4.31739	0.059349	2.80817008
H	-5.9091582	0.005577	0.93204498
C	-1.562724	0.076668	2.62314606
C	-1.864784	0.153725	3.99150395
C	0.73142803	0.085274	3.12627697
C	-0.830911	0.19448601	4.92816782
H	-2.893281	0.18640099	4.32676792
C	0.50394499	0.15881801	4.49170017
H	1.74059904	0.058388	2.73259497
N	-0.259721	0.043045	2.21826911
H	1.33135998	0.190357	5.18974781
H	-5.0554938	-0.051409	-1.410293
C	-1.1342551	0.275318	6.3286562
N	-1.377162	0.340893	7.46386003
C	0.034464	-0.251177	-1.966171
C	0.092723	0.75539398	-2.9499631
C	0.012099	-1.597726	-2.4382551
C	0.12654699	0.45775601	-4.3133068
H	0.106327	1.79664004	-2.6420679
C	0.04887	-1.894768	-3.8144519
C	0.105778	-0.871934	-4.7532511
H	0.033652	-2.9243519	-4.1610069
H	0.13445701	-1.104767	-5.8138819
C	-0.05951	-2.645083	-1.4155999
C	-0.126251	-4.0230932	-1.673074
C	-0.136019	-3.0697851	0.89312702
C	-0.197035	-4.9240999	-0.609524
H	-0.127698	-4.3928909	-2.690093
C	-0.201769	-4.4422569	0.71018499
H	-0.140078	-2.641952	1.88872099
N	-0.06473	-2.1960731	-0.126749
H	-0.257634	-5.1116118	1.55973995
H	0.169441	1.26587796	-5.0404272
C	-0.26743	-6.3342352	-0.867328
N	-0.324613	-7.4771819	-1.073247
C	0.21757001	1.99590695	0.036367
C	1.56372404	2.46974611	0.040432
C	-0.791215	2.97932506	0.032972
C	1.85815597	3.84703898	0.045545
C	-0.49604	4.34359694	0.034439
H	-1.83218	2.67025304	0.023986
C	0.83322698	4.78521395	0.04203
H	2.88739109	4.19490623	0.053025
C	2.61385012	1.44753206	0.03031
C	3.99292493	1.70540798	-0.007289
C	3.04311609	-0.861567	0.040505
C	4.89674282	0.641958	-0.01782
H	4.36151314	2.72252798	-0.032948
C	4.41670418	-0.678171	0.0075
H	2.61668491	-1.857621	0.056916
H	5.08827591	-1.5277621	-0.001619
N	2.16665196	0.158307	0.054239
H	1.06407595	5.84665918	0.046356
H	-1.305781	5.07016993	0.02996
C	6.30800009	0.90025198	-0.057255
N	7.4518609	1.10658205	-0.089031

Table S1(72). Optimized parameter of T_1 state of **1_*6*-CN**.

Atoms	X	Y	Z
Ir	-0.11378	-0.029294	0.045179
C	-2.0925419	-0.008377	0.193627
C	-3.0175979	-0.056839	-0.875794
C	-2.6252301	0.042319	1.526003
C	-4.3909392	-0.056875	-0.648626
H	-2.645751	-0.084549	-1.893759
C	-4.0079269	0.034888	1.74181199
C	-4.888679	-0.012925	0.66031599
H	-4.4156852	0.063935	2.74702907
H	-5.959621	-0.01532	0.83898401
C	-1.653138	0.094376	2.62636089
C	-1.9793381	0.189771	3.98477411
C	0.63191402	0.119222	3.15016198
C	-0.954422	0.25076401	4.93256092
H	-3.011971	0.221073	4.30678797
C	0.383845	0.216518	4.51264191
H	1.64563406	0.087161	2.76842499
N	-0.350349	0.055387	2.23857689
H	1.20207202	0.264485	5.22040081
H	-5.0798821	-0.091016	-1.488009
C	-1.27395	0.35172099	6.32869196
N	-1.530444	0.43418801	7.45931911
C	-0.031631	-0.231169	-1.979443
C	-0.032186	0.79106599	-2.939431
C	0.042094	-1.579659	-2.4353859
C	0.025717	0.50626999	-4.3071499
H	-0.089331	1.82730901	-2.6191831
C	0.108536	-1.856107	-3.8152981
C	0.096642	-0.82212	-4.7452159
H	0.173952	-2.882354	-4.1665292
H	0.14726301	-1.048154	-5.8070011
C	0.042866	-2.6170249	-1.40431
C	-0.027985	-3.975498	-1.628736
C	0.085338	-3.0120721	0.95754802
C	-0.038835	-4.906105	-0.552085
H	-0.076422	-4.34727	-2.6461179
C	0.019484	-4.3618612	0.78580201
H	0.135362	-2.5780461	1.94956398
N	0.098832	-2.11221	-0.087908
H	0.013204	-5.0183272	1.64883602
H	0.016331	1.31808496	-5.0305638
C	-0.103497	-6.2903771	-0.781759
N	-0.156384	-7.4493499	-0.968345
C	0.247007	1.99889505	0.091884
C	1.59934604	2.42692494	0.050913
C	-0.742156	2.99581504	0.17492101
C	1.92769206	3.79486108	0.08168
C	-0.41012	4.35240602	0.19929001
H	-1.789659	2.7148459	0.207882
C	0.92766798	4.75750399	0.152784
H	2.9643259	4.11654711	0.052685
C	2.62091494	1.37700105	-0.015475
C	4.00314379	1.60286605	-0.097602
C	2.98312092	-0.939761	-0.055939
C	4.87460804	0.51451498	-0.158037
H	4.39761782	2.61005092	-0.118512
C	4.35977221	-0.791268	-0.135683
H	2.52342892	-1.919974	-0.038543
H	5.0071311	-1.658199	-0.180568
N	2.14170504	0.104836	0.005467
H	1.18699706	5.81176519	0.175074
H	-1.200998	5.09652996	0.254684
C	6.29114008	0.73454398	-0.24451
N	7.4380641	0.91003603	-0.314198

Table S1(73). Optimized parameter of S_0 state of **1_7-CN**.

Atoms	X	Y	Z
Ir	0.000571	-0.025651	0.05739
C	-2.018199	-0.00633	0.231056
C	-2.9722409	-0.037618	-0.805552
C	-2.532115	0.030888	1.56400204
C	-4.3438439	-0.033653	-0.549947
H	-2.632473	-0.061021	-1.8365951
C	-3.91942	0.031815	1.81713295
C	-4.8256741	-0.000118	0.76602602
H	-4.29596	0.056719	2.83557105
H	-5.89362	0.000054	0.96419102
C	-1.54896	0.076567	2.64350891
C	-1.8459181	0.154569	4.01779413
C	0.74504602	0.091038	3.13350296
C	-0.829585	0.200027	4.95395994
H	-2.8760099	0.183706	4.35006714
C	0.50625098	0.168014	4.51004887
H	1.75657594	0.066077	2.74551702
H	-1.053298	0.261718	6.01332712
N	-0.243167	0.044215	2.23529792
H	-5.046402	-0.05701	-1.3802691
C	1.60279405	0.214701	5.42449188
N	2.49602294	0.25284901	6.16979694
C	0.039454	-0.265223	-1.954097
C	0.09852	0.738572	-2.9413309
C	0.013884	-1.61481	-2.423856
C	0.130413	0.437197	-4.3032718
H	0.114174	1.78064704	-2.6366041
C	0.048549	-1.914122	-3.801461
C	0.106621	-0.894105	-4.7413468
H	0.030852	-2.9444339	-4.144855
H	0.133825	-1.12816	-5.8016591
C	-0.058377	-2.661227	-1.406898
C	-0.128987	-4.04461	-1.660407
C	-0.132333	-3.0752871	0.90089899
C	-0.200483	-4.946805	-0.615222
H	-0.131564	-4.4102788	-2.6794491
C	-0.202596	-4.4590049	0.70568299
H	-0.13414	-2.653996	1.89935505
H	-0.256371	-6.0130739	-0.805241
N	-0.060173	-2.210228	-0.114903
H	0.17404599	1.24351203	-5.0323048
C	-0.276064	-5.336977	1.83026004
N	-0.335719	-6.0528131	2.74612594
C	0.23666701	1.98655701	0.040734
C	1.58576405	2.4585979	0.041056
C	-0.769813	2.97299004	0.0404
C	1.88191903	3.8373549	0.045811
C	-0.471447	4.33593702	0.041848
H	-1.811517	2.66661406	0.033829
C	0.85933799	4.77619696	0.045994
H	2.9118309	4.18239117	0.050323
C	2.63535094	1.44250202	0.026849
C	4.01977491	1.69673705	-0.016446
C	3.05448794	-0.86551	0.032796
C	4.92499399	0.651847	-0.033022
H	4.3840189	2.71598911	-0.041704
C	4.43928385	-0.669587	-0.007833
H	2.63476205	-1.86445	0.050084
H	5.99206114	0.842475	-0.067457
N	2.18646193	0.150077	0.052622
H	1.09097195	5.83738184	0.050305
H	-1.279734	5.06409311	0.039942
C	5.32018805	-1.794139	-0.025621
N	6.03816509	-2.7101631	-0.039951

Table S1(74). Optimized parameter of T_1 state of **1_7-CN**.

Atoms	X	Y	Z
Ir	-0.05172	-0.008617	0.054978
C	-2.025615	0.021138	0.193627
C	-2.964638	-0.02251	-0.862222
C	-2.5421071	0.086074	1.57332098
C	-4.3335762	0.016	-0.63254
H	-2.5988309	-0.06998	-1.882686
C	-3.946456	0.12754001	1.77802205
C	-4.8140101	0.093529	0.70060098
H	-4.3530731	0.183593	2.78265691
H	-5.8856511	0.125119	0.88072199
C	-1.5895129	0.085825	2.62923908
C	-1.8745101	0.130768	4.02852488
C	0.72736901	0.070856	3.11164904
C	-0.872207	0.152	4.95828676
H	-2.907506	0.145365	4.35878897
C	0.49841601	0.12958901	4.50691605
H	1.74351597	0.046575	2.73132801
H	-1.090925	0.184717	6.01985121
N	-0.243311	0.039122	2.21675706
H	-5.0360851	-0.00703	-1.460179
C	1.58912504	0.159541	5.39877415
N	2.49330807	0.18485799	6.14408588
C	0.013852	-0.272603	-1.9650691
C	0.07261	0.71951997	-2.95873
C	0.013842	-1.62923	-2.4079659
C	0.122237	0.39863899	-4.3168469
H	0.070837	1.76559603	-2.667203
C	0.065729	-1.94762	-3.7802291
C	0.118565	-0.938712	-4.7332411
H	0.06569	-2.982095	-4.1103921
H	0.157607	-1.188067	-5.7895169
C	-0.036844	-2.664129	-1.375072
C	-0.071064	-4.0522552	-1.608756
C	-0.096384	-3.050313	0.94469601
C	-0.119685	-4.9392552	-0.549297
H	-0.062758	-4.433516	-2.621778
C	-0.133001	-4.4362249	0.766671
H	-0.105063	-2.6078999	1.93414104
H	-0.148399	-6.0090518	-0.724863
N	-0.049831	-2.203006	-0.087544
H	0.162315	1.19413304	-5.0573382
C	-0.183571	-5.3036251	1.90122497
N	-0.223796	-6.0114198	2.823982
C	0.220478	1.99981701	0.071659
C	1.57207799	2.45508504	0.059557
C	-0.783806	2.98477602	0.11472
C	1.87372601	3.83112788	0.090701
C	-0.476342	4.34555912	0.14043
H	-1.826087	2.68187308	0.115717
C	0.85631698	4.77541685	0.129462
H	2.90497708	4.17040491	0.087608
C	2.6175139	1.43387699	0.016644
C	4.00104189	1.68555999	-0.043635
C	3.02690792	-0.875063	-0.025176
C	4.90085316	0.63694	-0.094064
H	4.36914587	2.70340991	-0.055289
C	4.41094398	-0.682786	-0.085778
H	2.60463309	-1.872762	-0.017095
H	5.96789694	0.82428098	-0.141904
N	2.16630507	0.144972	0.028939
H	1.09547997	5.83441591	0.15219299
H	-1.2803921	5.07707691	0.16897701
C	5.28798819	-1.8097791	-0.13854
N	6.00287724	-2.7269239	-0.182212

Table S2. Excitation characters of **1**.

state	λ_{ex} (nm)	f	Excitation characters.
S_1	446.2	0.041	H → L(86%)
S_2	432.7	0.004	H → L+1(86%)
S_3	425.3	0.007	H → L+2(91%)
S_4	399.0	0.013	H-1 → L(85%), H-1 → L+1(12%)
S_5	392.1	0.050	H-1 → L+1(85%), H-1 → L(11%)
S_6	384.4	0.004	H → L+3(97%)
S_7	373.5	0.059	H-1 → L+2(86%)
S_8	360.4	0.006	H → L+4(92%)
S_9	354.2	0.035	H-2 → L(80%)
S_{10}	351.2	0.001	H → L+5(90%)
S_{11}	349.7	0.025	H-1 → L+3(85%)
S_{12}	348.2	0.024	H-2 → L+1(20%), H-2 → L+2(60%)
S_{13}	340.3	0.034	H-3 → L(29%), H-2 → L+1(36%), H-2 → L+2(18%)
S_{14}	334.4	0.045	H-3 → L(51%), H-2 → L+1(25%)
S_{15}	332.3	0.015	H-3 → L+1(78%)
S_{16}	327.8	0.009	H-1 → L+4(84%)
S_{17}	323.4	0.009	H-3 → L+2(14%), H-1 → L+5(75%)
S_{18}	320.9	0.050	H-3 → L+2(60%), H-1 → L+5(18%)
S_{19}	317.0	0.027	H-2 → L+3(80%)
S_{20}	308.3	0.010	H-4 → L(57%)
S_{21}	303.2	0.001	H-4 → L(18%), H-4 → L+1(30%), H-3 → L+3(20%), H-2 → L+4(14%)
S_{22}	302.0	0.012	H-4 → L+1(33%), H-3 → L+3(43%)
S_{23}	299.9	0.013	H-5 → L(18%), H-3 → L+3(22%), H-2 → L+4(30%)
S_{24}	298.2	0.033	H-5 → L(33%), H-4 → L+2(17%), H-2 → L+4(16%), H-2 → L+5(20%)
S_{25}	297.0	0.084	H-4 → L+2(64%)
S_{26}	294.0	0.035	H-5 → L+1(37%), H-2 → L+5(22%)
S_{27}	290.6	0.034	H-5 → L+1(29%), H-2 → L+5(22%)
S_{28}	287.7	0.055	H-6 → L(16%), H-5 → L+2(56%)
S_{29}	287.3	0.238	H-6 → L(42%), H-3 → L+4(13%)
S_{30}	285.7	0.012	H-7 → L(21%), H-6 → L+1(19%), H-4 → L+3(12%), H-3 → L+4(22%)

Table S3(1). Excitation characters of **1**_*2*-NH_2

state	λ_{ex} (nm)	*f*	Excitation characters.
S_1	414.8	0.033	H → L(92%)
S_2	407.8	0.011	H → L+1(71%), H → L+2(21%)
S_3	407.7	0.011	H → L+1(21%), H → L+2(71%)
S_4	386.9	0.061	H-1 → L(79%)
S_5	386.8	0.061	H-2 → L(79%)
S_6	384.6	0.025	H-2 → L+2(34%), H-1 → L+2(34%)
S_7	377.7	0.026	H-2 → L+1(37%), H-1 → L+2(38%)
S_8	377.6	0.027	H-2 → L+1(37%), H-1 → L+1(38%)
S_9	376.7	0.008	H → L+2(65%)
S_{10}	363.7	0.069	H-2 → L+2(34%), H-1 → L+1(34%), H → L+3(19%)
S_{11}	358.3	0.011	H-1 → L+3(82%)
S_{12}	358.2	0.011	H-2 → L+3(82%)
S_{13}	348.3	0.027	H → L+4(71%)
S_{14}	348.2	0.027	H → L+5(71%)
S_{15}	342.5	0.008	H-4 → L(59%)
S_{16}	342.5	0.007	H-3 → L(59%)
S_{17}	335.6	0.016	H-5 → L(19%), H-4 → L+2(13%), H-3 → L+1(14%), H-2 → L+4(23%), H-1 → L+5(23%)
S_{18}	335.2	0.006	H-4 → L+2(12%), H-3 → L+1(16%), H-3 → L+2(11%), H-2 → L+5(11%), H-1 → L+4(13%), H-1 → L+5(11%)
S_{19}	334.6	0.055	H-4 → L+2(15%), H-3 → L+1(14%), H-3 → L+2(11%), H-2 → L+5(10%), H-1 → L+4(10%)
S_{20}	334.6	0.054	H-4 → L+1(16%), H-4 → L+2(11%), H-3 → L+2(13%), H-2 → L+4(11%)
S_{21}	333.5	0.050	H-5 → L(17%), H-4 → L+1(24%), H-3 → L+2(23%)
S_{22}	331.8	0.033	H-2 → L+5(25%), H-1 → L+4(31%)
S_{23}	331.8	0.033	H-2 → L+4(28%), H-1 → L+5(27%)
S_{24}	331.0	0.049	H-5 → L(15%), H-4 → L+2(14%), H-3 → L+1(12%), H-2 → L+5(26%), H-1 → L+4(20%)
S_{25}	324.7	0.058	H-5 → L+1(46%), H-5 → L+2(11%), H-4 → L+3(19%)
S_{26}	324.7	0.058	H-5 → L+1(11%), H-5 → L+2(46%), H-3 → L+3(19%)
S_{27}	321.5	0.563	H-5 → L(35%), H-2 → L+4(12%), H-1 → L+5(12%)
S_{28}	318.8	0.086	H-5 → L+2(16%), H-3 → L+3(65%)
S_{29}	318.8	0.086	H-5 → L+1(16%), H-4 → L+3(65%)
S_{30}	311.0	0.025	H-5 → L+3(91%)

Table S3(2). Excitation characters of **1**_*3*-NH_2

state	λ_{ex} (nm)	f	Excitation characters.
S_1	479.6	0.011	H → L(96%)
S_2	465.6	0.012	H → L+1(87%)
S_3	465.5	0.013	H → L+2(86%)
S_4	456.1	0.036	H-1 → L(80%)
S_5	456.0	0.037	H-2 → L(80%)
S_6	446.9	0.003	H-2 → L+1(35%), H-2 → L+2(12%), H-1 → L+1(13%), H-1 → L+2(34%)
S_7	439.7	0.046	H-2 → L+1(28%), H-2 → L+2(19%), H-1 → L+1(19%), H-1 → L+2(29%)
S_8	439.6	0.048	H-2 → L+1(19%), H-2 → L+2(28%), H-1 → L+1(29%), H-1 → L+2(18%)
S_9	428.8	0.016	H-2 → L+1(12%), H-2 → L+2(26%), H-1 → L+1(25%), H-1 → L+2(12%), H → L+3(22%)
S_{10}	405.2	0.074	H → L+3(74%)
S_{11}	402.6	0.002	H-1 → L+3(90%)
S_{12}	402.6	0.002	H-2 → L+3(90%)
S_{13}	379.9	0.037	H → L+4(80%), H → L+5(12%)
S_{14}	379.8	0.036	H → L+4(12%), H → L+5(79%)
S_{15}	373.3	0.000	H-2 → L+5(39%), H-1 → L+4(41%)
S_{16}	370.8	0.006	H-2 → L+4(43%), H-1 → L+5(43%)
S_{17}	370.7	0.005	H-2 → L+5(44%), H-1 → L+4(42%)
S_{18}	367.7	0.017	H-2 → L+4(40%), H-1 → L+5(40%)
S_{19}	308.8	0.009	H-5 → L(91%)
S_{20}	307.2	0.004	H-5 → L+2(10%), H-4 → L(54%), H-3 → L(21%)
S_{21}	307.2	0.004	H-5 → L+1(11%), H-4 → L(20%), H-3 → L(54%)
S_{22}	302.8	0.035	H-5 → L+1(61%), H-5 → L+2(14%), H-4 → L(11%)
S_{23}	302.8	0.035	H-5 → L+1(15%), H-5 → L+2(60%), H-3 → L(11%)
S_{24}	300.2	0.001	H-4 → L+1(13%), H-4 → L+2(29%), H-3 → L+1(31%), H-3 → L+2(13%)
S_{25}	298.9	0.028	H-4 → L+1(30%), H-4 → L+2(12%), H-3 → L+1(11%), H-3 → L+2(29%)
S_{26}	298.9	0.027	H-4 → L+1(12%), H-4 → L+2(28%), H-3 → L+1(30%)
S_{27}	296.5	0.044	H-4 → L+1(26%), H-4 → L+2(15%), H-3 → L+1(13%), H-3 → L+2(28%)
S_{28}	292.3	0.001	H-2 → L+8(11%), H-1 → L+7(12%), H → L+6(70%)
S_{29}	291.8	0.047	H-7 → L(40%)
S_{30}	291.8	0.047	H-8 → L(40%)

Table S3(4). Excitation characters of **1**_*4*-**NH$_2$**

state	λ_{ex} (nm)	f	Excitation characters.
S_1	441.9	0.001	H → L(98%)
S_2	421.3	0.013	H-2 → L(11%), H → L+1(65%), H → L+2(20%)
S_3	421.2	0.014	H-1 → L(12%), H → L+1(20%), H → L+2(64%)
S_4	413.4	0.062	H-1 → L(83%), H → L+2(13%)
S_5	413.3	0.063	H-2 → L(84%), H → L+1(12%)
S_6	398.8	0.000	H-2 → L+1(34%), H-2 → L+2(12%), H-1 → L+1(13%), H-1 → L+2(35%)
S_7	393.2	0.068	H-2 → L+1(23%), H-2 → L+2(24%), H-1 → L+1(25%), H-1 → L+2(23%)
S_8	393.2	0.069	H-2 → L+1(26%), H-2 → L+2(23%), H-1 → L+1(23%), H-1 → L+2(24%)
S_9	379.9	0.000	H-2 → L+2(29%), H-1 → L+1(28%), H → L+3(22%)
S_{10}	366.4	0.003	H → L+3(73%)
S_{11}	356.7	0.005	H-1 → L+3(86%)
S_{12}	356.6	0.005	H-2 → L+3(86%)
S_{13}	347.7	0.048	H-3 → L(54%), H → L+4(21%)
S_{14}	347.7	0.048	H-4 → L(52%), H → L+5(22%)
S_{15}	344.1	0.124	H-5 → L(73%)
S_{16}	341.2	0.026	H-3 → L(25%), H → L+4(68%)
S_{17}	341.1	0.027	H-4 → L(24%), H → L+5(67%)
S_{18}	333.8	0.000	H-5 → L(15%), H-2 → L+5(20%), H-1 → L+4(25%)
S_{19}	332.6	0.010	H-4 → L+1(27%), H-3 → L+2(22%), H-2 → L+4(12%), H-1 → L+5(12%)
S_{20}	332.5	0.010	H-5 → L+2(23%), H-4 → L+2(14%), H-3 → L+1(18%), H-2 → L+5(14%)
S_{21}	329.6	0.017	H-2 → L+4(23%), H-2 → L+5(15%), H-1 → L+4(19%), H-1 → L+5(26%)
S_{22}	328.4	0.004	H-2 → L+5(29%), H-1 → L+4(24%)
S_{23}	328.4	0.004	H-4 → L+1(13%), H-2 → L+4(27%), H-1 → L+5(27%)
S_{24}	327.6	0.003	H-4 → L+1(14%), H-4 → L+2(30%), H-3 → L+1(30%), H-3 → L+2(17%)
S_{25}	325.4	0.004	H-5 → L+1(21%), H-5 → L+2(30%), H-4 → L+2(26%), H-3 → L+2(17%)
S_{26}	325.3	0.004	H-5 → L+1(51%), H-5 → L+2(25%), H-3 → L+2(11%)
S_{27}	322.3	0.202	H-4 → L+1(15%), H-3 → L+2(15%), H-2 → L+4(19%), H-1 → L+5(19%)
S_{28}	306.5	0.005	H-3 → L+3(86%)
S_{29}	306.5	0.005	H-4 → L+3(84%)
S_{30}	304.3	0.091	H-5 → L+3(79%)

Table S3(5). Excitation characters of **1**_*5*-NH_2

state	λ_{ex} (nm)	*f*	Excitation characters.
S_1	427.5	0.005	H → L+1(80%), H → L+2(13%)
S_2	427.4	0.005	H → L+1(15%), H → L+2(80%)
S_3	425.3	0.015	H → L(91%)
S_4	406.8	0.007	H-2 → L+1(28%), H-2 → L+2(15%), H-1 → L+1(17%), H-1 → L+2(33%)
S_5	403.8	0.016	H-2 → L+2(15%), H-1 → L(67%)
S_6	403.3	0.016	H-2 > L(62%), H-2 → L+2(15%), H-1 → L+1(14%)
S_7	397.6	0.108	H-2 → L(24%), H-2 → L+2(28%), H-1 → L+1(29%)
S_8	397.3	0.109	H-2 → L+1(32%), H-1 → L(19%), H-1 → L+2(31%)
S_9	382.8	0.061	H-2 → L+1(16%), H-2 → L+2(32%), H-1 → L+1(30%), H-1 → L+2(16%)
S_{10}	337.4	0.005	H → L+3(86%)
S_{11}	328.8	0.001	H-1 → L+3(84%)
S_{12}	328.6	0.001	H-2 → L+3(83%)
S_{13}	320.6	0.076	H-4 → L+2(24%), H-3 → L+1(29%), H → L+4(13%)
S_{14}	320.6	0.074	H-5 → L(26%), H-3 → L+2(26%), H → L+5(14%)
S_{15}	319.1	0.263	H-3 → L(59%)
S_{16}	316.0	0.017	H-4 → L(60%), H-3 → L+1(19%)
S_{17}	315.8	0.018	H-5 → L(55%), H-3 → L+2(23%)
S_{18}	315.1	0.030	H-5 → L+2(37%), H-4 → L+1(17%), H-3 → L(20%)
S_{19}	314.9	0.003	H-5 → L+2(21%), H-4 → L+1(31%), H-3 → L+2(25%)
S_{20}	314.8	0.004	H-5 → L+1(23%), H-4 → L+2(26%), H-3 → L+1(27%)
S_{21}	314.1	0.002	H-5 → L+1(42%), H-4 → L+2(36%)
S_{22}	311.3	0.105	H → L+4(16%), H → L+5(56%)
S_{23}	311.1	0.106	H → L+4(55%), H → L+5(16%)
S_{24}	304.1	0.001	H-2 → L+4(22%), H-1 → L+4(29%), H-1 → L+5(38%)
S_{25}	303.6	0.004	H-2 → L+5(44%), H-1 → L+4(34%), H-1 → L+5(13%)
S_{26}	303.6	0.003	H-2 → L+4(53%), H-1 → L+5(29%)
S_{27}	301.6	0.128	H-2 → L+4(17%), H-2 → L+5(31%), H-1 → L+4(25%), H-1 → L+5(14%)
S_{28}	277.7	0.003	H → L+6(49%), H → L+7(33%)
S_{29}	274.1	0.061	H-6 → L+2(13%), H-1 → L+6(15%), H → L+9(33%)
S_{30}	274.1	0.056	H-6 → L+1(12%), H → L+8(40%)

Table S3(6). Excitation characters of **1**_*6*-**NH₂**

state	λ_{ex} (nm)	f	Excitation characters.
S_1	418.5	0.015	H → L(98%)
S_2	402.4	0.031	H-1 → L(24%), H → L+1(73%)
S_3	402.2	0.033	H-2 → L(25%), H → L+2(71%)
S_4	399.7	0.029	H-1 → L(73%), H → L+1(22%)
S_5	399.6	0.028	H-2 → L(71%), H → L+2(24%)
S_6	385.6	0.006	H-2 → L+1(33%), H-2 → L+2(15%), H-1 → L+1(18%), H-1 → L+2(32%)
S_7	381.4	0.048	H-2 → L+1(11%), H-2 → L+2(37%), H-1 → L+1(37%), H-1 → L+2(13%)
S_8	381.4	0.050	H-2 → L+1(38%), H-2 → L+2(11%), H-1 → L+1(12%), H-1 → L+2(36%)
S_9	373.3	0.029	H-2 → L+1(16%), H-2 → L+2(31%), H-1 → L+1(28%), H-1 → L+2(16%)
S_{10}	351.0	0.027	H → L+3(91%)
S_{11}	339.9	0.028	H-1 → L+3(91%)
S_{12}	339.8	0.028	H-2 → L+3(90%)
S_{13}	332.8	0.035	H → L+4(85%)
S_{14}	332.7	0.036	H → L+5(86%)
S_{15}	322.5	0.002	H-2 → L+4(36%), H-2 → L+5(11%), H-1 → L+4(14%), H-1 → L+5(35%)
S_{16}	320.7	0.038	H-2 → L+4(37%), H-2 → L+5(12%), H-1 → L+4(13%), H-1 → L+5(35%)
S_{17}	320.7	0.037	H-2 → L+4(11%), H-2 → L+5(37%), H-1 → L+4(35%), H-1 → L+5(14%)
S_{18}	314.8	0.038	H-2 → L+4(12%), H-2 → L+5(34%), H-1 → L+4(33%), H-1 → L+5(12%)
S_{19}	287.9	0.008	H-3 → L(89%)
S_{20}	287.9	0.008	H-4 → L(89%)
S_{21}	283.2	0.091	H-5 → L(66%)
S_{22}	280.7	0.069	H → L+6(22%), H → L+7(52%)
S_{23}	280.0	0.074	H-6 → L(23%), H-5 → L+1(10%), H-2 → L+7(17%)
S_{24}	280.0	0.072	H-7 → L(22%), H-1 → L+7(18%)
S_{25}	278.0	0.000	H-5 → L(15%), H-4 → L+1(17%), H-4 → L+2(15%), H-3 → L+1(24%), H-3 → L+2(20%)
S_{26}	277.6	0.005	H-7 → L(16%), H-4 → L+1(12%), H-4 → L+2(22%), H-3 → L+1(17%)
S_{27}	277.5	0.006	H-6 → L(15%), H-4 → L+1(19%), H-3 → L+2(22%)
S_{28}	276.7	0.199	H-8 → L(11%), H-4 → L+1(18%), H-4 → L+2(20%), H-3 → L+1(17%), H-3 → L+2(18%)
S_{29}	274.8	0.021	H-1 → L+7(36%), H → L+11(25%)
S_{30}	274.7	0.023	H-2 → L+7(36%), H → L+12(24%)

Table S3(7). Excitation characters of **1_*7*-NH$_2$**

state	λ_{ex} (nm)	*f*	Excitation characters.
S_1	418.4	0.008	H → L(97%)
S_2	410.2	0.005	H → L+1(95%)
S_3	410.2	0.005	H → L+2(95%)
S_4	390.7	0.040	H-2 → L(12%), H-1 → L(78%)
S_5	390.6	0.041	H-2 → L(78%), H-1 → L(12%)
S_6	387.1	0.007	H-2 → L+2(46%), H-1 → L+1(47%)
S_7	379.7	0.041	H-2 → L+1(11%), H-2 → L+2(35%), H-1 → L+1(33%), H-1 → L+2(12%)
S_8	379.6	0.043	H-2 → L+1(34%), H-2 → L+2(11%), H-1 → L+1(11%), H-1 → L+2(34%)
S_9	376.3	0.007	H-2 → L+1(11%), H-1 → L+2(11%), H → L+3(76%)
S_{10}	360.1	0.112	H-2 → L+1(35%), H-1 → L+2(35%), H → L+3(20%)
S_{11}	356.4	0.021	H-1 → L+3(96%)
S_{12}	356.3	0.021	H-2 → L+3(96%)
S_{13}	345.6	0.040	H → L+4(95%)
S_{14}	345.5	0.040	H → L+5(95%)
S_{15}	331.3	0.017	H-2 → L+4(22%), H-2 → L+5(23%), H-1 → L+4(29%), H-1 → L+5(21%)
S_{16}	330.2	0.029	H-2 → L+5(40%), H-1 → L+4(40%)
S_{17}	330.2	0.029	H-2 → L+4(38%), H-1 → L+5(43%)
S_{18}	329.3	0.021	H-2 → L+4(27%), H-2 → L+5(23%), H-1 → L+4(17%), H-1 → L+5(26%)
S_{19}	320.5	0.011	H-3 → L(91%)
S_{20}	320.4	0.012	H-4 → L(91%)
S_{21}	313.8	0.023	H-4 → L+2(33%), H-3 → L+1(51%)
S_{22}	313.2	0.080	H-4 → L+1(45%), H-3 → L+2(45%)
S_{23}	313.1	0.080	H-4 → L+2(54%), H-3 → L+1(36%)
S_{24}	311.4	0.046	H-4 → L+1(46%), H-3 → L+2(45%)
S_{25}	306.5	0.209	H-5 → L(77%)
S_{26}	304.7	0.011	H-5 → L+1(72%), H-4 → L+3(15%)
S_{27}	304.7	0.010	H-5 → L+2(72%), H-3 → L+3(15%)
S_{28}	296.6	0.091	H-5 → L+2(14%), H-3 → L+3(70%)
S_{29}	296.6	0.091	H-5 → L+1(14%), H-4 → L+3(71%)
S_{30}	288.7	0.112	H-5 → L+3(93%)

Table S3(8). Excitation characters of **1_*2*-SO₂Me**

state	λ_{ex} (nm)	f	Excitation characters.
S_1	433.7	0.013	H → L(98%)
S_2	419.2	0.005	H → L+1(96%)
S_3	418.2	0.006	H → L+2(96%)
S_4	409.7	0.041	H-2 → L(16%), H-1 → L(79%)
S_5	408.9	0.049	H-2 → L(79%), H-1 → L(16%)
S_6	398.9	0.008	H-2 → L+1(25%), H-2 → L+2(22%), H-1 → L+1(27%), H-1 → L+2(24%)
S_7	391.5	0.051	H-2 → L+2(48%), H-1 → L+1(45%)
S_8	391.0	0.060	H-2 → L+1(47%), H-1 → L+2(46%)
S_9	378.9	0.042	H-2 → L+1(22%), H-2 → L+2(25%), H-1 → L+1(22%), H-1 → L+2(23%)
S_{10}	349.9	0.036	H → L+3(91%)
S_{11}	338.1	0.009	H-1 → L+3(96%)
S_{12}	337.9	0.008	H-2 → L+3(96%)
S_{13}	329.7	0.030	H → L+4(94%)
S_{14}	328.9	0.029	H → L+5(95%)
S_{15}	317.7	0.004	H-2 → L+4(51%), H-1 → L+4(27%, H-1 > L+5(16%)
S_{16}	316.7	0.015	H-2 → L+4(33%), H-2 → L+5(13%), H-1 → L+4(24%), H-1 → L+5(27%)
S_{17}	316.6	0.012	H-2 → L+5(36%), H-1 → L+4(19%), H-1 → L+5(39%)
S_{18}	313.3	0.006	H-2 → L+5(43%), H-1 → L+4(25%), H-1 → L+5(14%)
S_{19}	294.9	0.031	H-3 → L(95%)
S_{20}	294.1	0.039	H-4 → L(95%)
S_{21}	288.3	0.051	H-5 → L(78%)
S_{22}	286.7	0.039	H-3 → L+1(89%)
S_{23}	286.3	0.099	H-4 → L+1(37%), H-3 → L+2(53%)
S_{24}	285.8	0.064	H-4 → L+2(87%)
S_{25}	284.1	0.702	H-5 → L(14%), H-4 → L+1(36%), H-3 → L+2(27%)
S_{26}	281.5	0.057	H-5 → L+1(91%)
S_{27}	281.1	0.044	H-5 → L+2(92%)
S_{28}	273.9	0.048	H-6 → L(62%), H → L+6(23%)
S_{29}	270.8	0.005	H-6 → L(20%), H → L+7(52%)
S_{30}	269.0	0.031	H-6 → L+1(26%), H → L+7(36%)

Table S3(9). Excitation characters of **1_*3*-SO_2Me**

state	λ_{ex} (nm)	*f*	Excitation characters.
S_1	400.4	0.007	H → L(98%)
S_2	392.0	0.004	H → L+1(96%)
S_3	391.3	0.005	H → L+2(96%)
S_4	377.2	0.046	H-1 → L(90%)
S_5	376.5	0.053	H-2 → L(91%)
S_6	371.5	0.004	H-2 → L+1(21%), H-2 → L+2(26%), H-1 → L+1(29%), H-1 → L+2(20%)
S_7	364.6	0.062	H-2 → L+1(26%), H-2 → L+2(20%), H-1 → L+1(18%), H-1 → L+2(30%)
S_8	364.3	0.070	H-2 → L+1(21%), H-2 → L+2(28%), H-1 → L+1(28%), H-1 → L+2(17%)
S_9	353.9	0.030	H-2 → L+1(22%), H-2 → L+2(20%), H-1 → L+1(17%), H-1 → L+2(23%), H → L+3(13%)
S_{10}	342.5	0.065	H → L+3(84%)
S_{11}	329.3	0.007	H-1 → L+3(95%)
S_{12}	329.1	0.007	H-2 → L+3(95%)
S_{13}	320.6	0.014	H → L+4(94%)
S_{14}	319.9	0.014	H → L+5(94%)
S_{15}	307.7	0.009	H-2 → L+4(12%), H-1 → L+4(71%)
S_{16}	306.7	0.019	H-2 → L+4(59%), H-1 → L+5(23%)
S_{17}	306.5	0.019	H-2 → L+5(65%), H-1 → L+5(19%)
S_{18}	305.4	0.004	H-2 → L+4(22%), H-2 → L+5(15%), H-1 → L+5(48%)
S_{19}	293.7	0.090	H → L+7(81%)
S_{20}	293.3	0.091	H → L+8(73%)
S_{21}	292.1	0.014	H → L+6(59%)
S_{22}	289.3	0.020	H-4 → L(28%), H-2 → L+6(39%)
S_{23}	289.2	0.015	H-3 → L(27%), H-1 → L+6(37%)
S_{24}	286.1	0.037	H-3 → L(40%), H-1 → L+6(35%)
S_{25}	285.5	0.038	H-4 → L(39%), H-2 → L+6(31%)
S_{26}	284.2	0.063	H-2 → L+8(25%), H-1 → L+7(36%)
S_{27}	282.2	0.035	H-4 → L+2(14%), H-3 → L+1(14%), H-3 → L+2(18%), H-1 → L+8(30%)
S_{28}	282.2	0.018	H-3 → L+1(28%), H-2 → L+7(23%), H-2 → L+8(15%)
S_{29}	281.8	0.039	H-4 → L+2(22%), H-2 → L+8(25%), H-1 → L+7(24%)
S_{30}	280.8	0.009	H-5 → L(57%), H-4 → L+1(21%)

Table S3(10). Excitation characters of **1_*4*-SO_2Me**

state	λ_{ex} (nm)	*f*	Excitation characters.
S_1	428.9	0.004	H → L+1(97%)
S_2	428.0	0.004	H → L+2(97%)
S_3	420.2	0.021	H → L(97%)
S_4	407.5	0.006	H-2 → L+2(33%), H-1 → L+1(43%)
S_5	402.3	0.001	H-2 → L(15%), H-2 → L+1(19%), H-1 → L(33%), H-1 → L+2(17%)
S_6	402.2	0.000	H-2 → L(32%), H-2 → L+2(20%), H-1 → L(12%), H-1 → L+1(16%)
S_7	397.2	0.088	H-2 → L+1(23%), H-1 → L(52%), H-1 → L+2(21%)
S_8	396.9	0.089	H-2 → L(49%), H-2 → L+2(25%), H-1 → L+1(22%)
S_9	390.0	0.041	H-2 → L+1(35%), H-1 → L+2(40%)
S_{10}	341.4	0.024	H → L+3(93%)
S_{11}	331.1	0.012	H-1 → L+3(93%)
S_{12}	330.9	0.012	H-2 → L+3(93%)
S_{13}	321.4	0.034	H → L+4(91%)
S_{14}	321.3	0.033	H → L+5(91%)
S_{15}	311.3	0.003	H-2 → L+5(33%), H-1 → L+4(58%)
S_{16}	310.4	0.011	H-2 → L+5(53%), H-1 → L+4(31%)
S_{17}	310.3	0.011	H-2 → L+4(43%), H-1 → L+5(41%)
S_{18}	308.1	0.000	H-2 → L+4(42%), H-1 → L+5(41%)
S_{19}	295.5	0.035	H-4 → L+1(52%), H-3 → L+1(16%), H-3 → L+2(16%)
S_{20}	295.2	0.045	H-4 → L(12%), H-4 → L+2(12%), H-3 → L(17%), H-3 → L+2(45%)
S_{21}	295.1	0.040	H-4 → L(16%), H-4 → L+1(12%), H-3 → L(11%), H-3 → L+2(47%)
S_{22}	294.5	0.115	H-4 → L+2(41%), H-3 → L+1(23%), H-3 → L+2(11%)
S_{23}	293.8	0.026	H-5 → L+1(34%), H-5 → L+2(29%), H-4 → L+2(13%)
S_{24}	293.6	0.040	H-5 → L+2(20%), H-4 → L+2(11%), H-3 → L(39%)
S_{25}	292.7	0.109	H-5 → L+1(51%), H-4 → L(30%)
S_{26}	292.5	0.088	H-5 → L+2(66%), H-3 → L(18%)
S_{27}	291.3	0.174	H-5 → L(88%)
S_{28}	284.5	0.000	H → L+6(77%)
S_{29}	281.5	0.004	H-1 → L+6(18%), H → L+7(73%)
S_{30}	281.4	0.004	H-2 → L+6(21%), H → L+8(70%)

Table S3(11). Excitation characters of **1_*5*-SO_2Me**

state	λ_{ex} (nm)	*f*	Excitation characters.
S_1	451.7	0.008	H → L+1(93%)
S_2	450.7	0.008	H → L+2(93%)
S_3	440.3	0.013	H → L(79%), H → L+3(14%)
S_4	427.0	0.002	H-2 → L+2(25%), H-1 → L+1(31%), H → L+3(28%)
S_5	421.8	0.002	H-2 → L+2(18%), H-1 → L+1(16%), H-1 → L+2(12%), H → L+3(38%)
S_6	418.8	0.045	H-2 → L(28%), H-2 → L+1(12%), H-2 → L+2(18%), H-1 → L+1(17%), H-1 → L+2(18%)
S_7	418.4	0.035	H-2 → L+1(25%), H-2 → L+2(24%), H-1 → L+1(22%), H-1 → L+2(23%)
S_8	417.0	0.032	H-1 → L(92%)
S_9	416.5	0.021	H-2 → L(65%)
S_{10}	401.4	0.019	H-2 → L+1(26%), H-1 → L+2(31%), H → L+3(15%)
S_{11}	400.7	0.011	H-2 → L+3(24%), H-1 → L+3(33%), H → L+4(29%)
S_{12}	400.6	0.010	H-2 → L+3(31%), H-1 → L+3(26%), H → L+5(29%)
S_{13}	392.6	0.093	H-2 → L+3(37%), H → L+4(46%)
S_{14}	392.4	0.094	H-1 → L+3(36%), H → L+5(48%)
S_{15}	377.5	0.003	H-2 → L+4(19%), H-1 → L+5(70%)
S_{16}	377.3	0.010	H-2 → L+4(33%), H-2 → L+5(31%), H-1 → L+4(11%), H-1 → L+5(20%)
S_{17}	377.1	0.008	H-2 → L+5(49%), H-1 → L+5(38%)
S_{18}	370.1	0.030	H-2 → L+4(32%), H-2 → L+5(12%), H-1 → L+4(12%), H-1 → L+5(32%)
S_{19}	308.7	0.014	H-3 → L(32%), H-3 → L+2(31%)
S_{20}	308.5	0.025	H-4 → L+1(28%), H-3 → L(12%), H-3 → L+1(46%)
S_{21}	308.2	0.020	H-4 → L(26%), H-4 → L+2(45%)
S_{22}	304.3	0.132	H-4 → L(29%), H-4 → L+2(13%), H-3 → L+1(27%)
S_{23}	304.2	0.115	H-4 → L+1(42%), H-3 → L(19%), H-3 → L+3(11%)
S_{24}	304.0	0.170	H-4 → L+2(20%), H-3 → L+2(50%)
S_{25}	303.0	0.056	H-5 → L(31%), H-5 → L+1(36%)
S_{26}	302.8	0.060	H-5 → L(35%), H-5 → L+1(29%)
S_{27}	302.5	0.048	H-5 → L(16%), H-5 → L+2(51%)
S_{28}	299.9	0.012	H-4 → L+3(61%)
S_{29}	299.8	0.007	H-5 → L+2(14%), H-3 → L+5(58%)
S_{30}	297.7	0.028	H-5 → L(11%), H-5 → L+3(61%)

Table S3(12). Excitation characters of **1_*6*-SO_2Me**

state	λ_{ex} (nm)	*f*	Excitation characters.
S_1	481.9	0.002	H → L(97%)
S_2	473.4	0.004	H → L+1(95%)
S_3	473.3	0.004	H → L+2(96%)
S_4	445.2	0.043	H-1 → L(89%)
S_5	444.9	0.043	H-2 → L(88%)
S_6	442.8	0.003	H-2 → L+1(22%), H-2 → L+2(27%), H-1 → L+1(26%), H-1 → L+2(22%)
S_7	431.8	0.088	H-2 → L+1(38%), H-1 → L+2(37%)
S_8	431.8	0.088	H-2 → L+2(38%), H-1 → L+1(38%)
S_9	412.9	0.020	H-2 → L+1(22%), H-2 → L+2(17%), H-1 → L+1(16%), H-1 → L+2(22%), H → L+3(20%)
S_{10}	386.0	0.069	H → L+3(78%)
S_{11}	375.5	0.018	H-1 → L+3(97%)
S_{12}	375.3	0.019	H-2 → L+3(97%)
S_{13}	365.3	0.044	H → L+4(95%)
S_{14}	365.1	0.044	H → L+5(95%)
S_{15}	351.8	0.006	H-2 → L+4(44%), H-1 → L+5(48%)
S_{16}	349.4	0.014	H-2 → L+4(28%), H-2 → L+5(20%), H-1 → L+4(25%), H-1 → L+5(23%)
S_{17}	349.3	0.014	H-2 → L+4(22%), H-2 → L+5(23%), H-1 → L+4(29%), H-1 → L+5(23%)
S_{18}	347.1	0.011	H-2 → L+5(50%), H-1 → L+4(40%)
S_{19}	327.2	0.016	H-3 → L(88%)
S_{20}	327.1	0.016	H-4 → L(88%)
S_{21}	321.2	0.018	H-4 → L+1(12%), H-4 → L+2(33%), H-3 → L+1(36%), H-3 → L+2(13%)
S_{22}	319.9	0.057	H-4 → L+1(42%), H-3 → L+2(44%)
S_{23}	319.8	0.058	H-4 → L+2(44%), H-3 → L+1(42%)
S_{24}	318.3	0.244	H-5 → L(17%), H-4 → L+1(32%), H-3 → L+2(29%)
S_{25}	315.4	0.039	H-5 → L(74%)
S_{26}	311.2	0.014	H-5 → L+1(91%)
S_{27}	311.1	0.013	H-5 → L+2(91%)
S_{28}	288.8	0.019	H-6 → L(85%)
S_{29}	287.9	0.009	H-6 → L+1(13%), H-4 → L+3(58%)
S_{30}	287.9	0.009	H-6 → L+2(12%), H-3 → L+3(59%)

Table S3(13). Excitation characters of **1_*7*-SO_2Me**

state	λ_{ex} (nm)	*f*	Excitation characters.
S_1	467.2	0.021	H → L(96%)
S_2	455.2	0.013	H → L+1(88%)
S_3	451.2	0.023	H → L+2(88%)
S_4	446.0	0.035	H-1 → L(86%)
S_5	443.1	0.044	H-2 → L(86%)
S_6	436.1	0.021	H-2 → L+2(23%), H-1 → L+1(65%)
S_7	427.5	0.044	H-2 → L+1(50%), H-1 → L+2(43%)
S_8	426.4	0.045	H-2 → L+2(69%), H-1 → L+1(26%)
S_9	414.3	0.024	H-2 → L+1(33%), H-1 → L+2(44%), H → L+3(13%)
S_{10}	387.6	0.039	H → L+3(83%)
S_{11}	380.8	0.012	H-1 → L+3(96%)
S_{12}	379.5	0.010	H-2 → L+3(96%)
S_{13}	361.4	0.024	H → L+4(86%)
S_{14}	360.8	0.026	H → L+5(87%)
S_{15}	352.4	0.006	H-1 → L+4(90%)
S_{16}	350.5	0.018	H-2 → L+4(45%), H-1 → L+5(48%)
S_{17}	349.4	0.008	H-2 → L+5(86%)
S_{18}	344.3	0.014	H-2 → L+4(40%), H-1 → L+5(46%)
S_{19}	318.6	0.020	H-3 → L(93%)
S_{20}	316.9	0.028	H-4 → L(94%)
S_{21}	311.7	0.022	H-5 → L(12%), H-3 → L+1(66%)
S_{22}	309.7	0.078	H-4 → L+1(51%), H-3 → L+2(39%)
S_{23}	309.3	0.058	H-5 → L(24%), H-4 → L+2(31%), H-3 → L+1(25%), H-3 → L+2(11%)
S_{24}	307.6	0.027	H-5 → L(28%), H-4 → L+2(57%)
S_{25}	304.5	0.845	H-5 → L(31%), H-4 → L+1(25%), H-3 → L+2(31%)
S_{26}	302.5	0.046	H-5 → L+1(92%)
S_{27}	300.7	0.034	H-5 → L+2(92%)
S_{28}	287.4	0.015	H-3 → L+3(78%)
S_{29}	286.8	0.013	H-4 → L+3(74%)
S_{30}	284.9	0.045	H-6 → L(79%)

Table S4. Calculated emission wavelengths of the chloro-, methoxy-, nitro- and cyano-substituted *fac*-Ir(ppy)$_3$.

Complexes	Emission wavelength (λ_{em}) (nm)	$\Delta\lambda_{em}$ (nm)[1)]
**1**	532	
**1**_*2*-**Cl**	530	-2
**1**_*3*-**Cl**	539	7
**1**_*4*-**Cl**	529	-3
**1**_*5*-**Cl**	548	16
**1**_*6*-**Cl**	538	6
**1**_*7*-**Cl**	546	14
**1**_*2*-**OMe**	520	-12
**1**_*3*-**OMe**	592	60
**1**_*4*-**OMe**	522	-10
**1**_*5*-**OMe**	562	30
**1**_*6*-**OMe**	517	-15
**1**_*7*-**OMe**	542	10
**1**_*2*-**NO**$_2$	730	198
**1**_*3*-**NO**$_2$	563	31
**1**_*4*-**NO**$_2$	803	271
**1**_*5*-**NO**$_2$	823	291
**1**_*6*-**NO**$_2$	931	399
**1**_*7*-**NO**$_2$	754	222
**1**_*2*-**CN**	587	55
**1**_*3*-**CN**	508	-24
**1**_*4*-**CN**	575	43
**1**_*5*-**CN**	568	36
**1**_*6*-**CN**	698	166
**1**_*7*-**CN**	589	57

1) A difference in λ_{em} in comparison with the complex **1**. The positive and negative values mean red and blue shifts, respectively.
